# Characterization of the mechanisms underlying sulfasalazine-induced ferroptotic cell death: role of protein disulfide isomerase-mediated NOS activation and NO accumulation: Mechanism of SAS-induced cell death

**DOI:** 10.3724/abbs.2025100

**Published:** 2025-08-21

**Authors:** Yi-Chen Jia, Jia-Ling Zhong, Xiangyu Hao, Bao Ting Zhu

**Affiliations:** 1 Shenzhen Key Laboratory of Steroid Drug Discovery and Development School of Medicine The Chinese University of Hong Kong Shenzhen 518172 China; 2 Shenzhen Bay Laboratory Shenzhen 518055 China

**Keywords:** sulfasalazine, ferroptosis, protein disulfide isomerise, nitric oxide synthase, nitric oxide, reactive oxygen species, lipid reactive oxygen species

## Abstract

Sulfasalazine (SAS), a clinically utilized anti-inflammatory drug, has been shown to induce ferroptosis by inhibiting system Xc ^−^ activity, thereby causing cellular glutathione depletion. Recently, protein disulfide isomerase (PDI) was shown to be an upstream mediator of the oxidative cell death (oxytosis/ferroptosis) induced by glutamate, erastin, RSL3 and SAS. The present study aims to further characterize the detailed biochemical and cellular mechanisms of SAS-induced ferroptosis in two cell lines, *i* . *e*., H9C2 rat cardiomyocytes and BRL-3A rat hepatocytes, with a focus on elucidating the critical role of PDI in mediating SAS-induced toxicity. We find that SAS can induce ferroptosis in H9C2 and BRL-3A cells, which is accompanied by a sequential increase in the buildup of cellular nitric oxide (NO), reactive oxygen species (ROS) and lipid-ROS. SAS activates PDI-mediated dimerization of inducible NO synthase (iNOS) and cellular accumulation of NO, and these effects are followed by ROS and lipid-ROS accumulation. Furthermore, SAS markedly upregulates the iNOS protein levels in these cells. Knockdown of *PDI* or pharmacological inhibition of PDI catalytic activity effectively suppresses SAS-induced iNOS dimerization, abrogates SAS-induced accumulation of NO, ROS and lipid-ROS, and prevents ferroptosis. On the other hand, PDI activation through the use of TrxR1 inhibitors sensitizes these cells to SAS-induced ferroptosis. These findings provide further experimental support for a pivotal role of PDI in SAS-induced cytotoxicity through the activation of the PDI-NOS-NO axis, which then leads to the accumulation of cellular ROS and lipid-ROS and ultimately the induction of oxidative cell death.

## Introduction

Sulfasalazine (SAS), a well-established drug for treating inflammatory disorders such as Crohn’s disease and ulcerative colitis [ 1– 3], has recently been identified as a potent inducer of ferroptosis [4]. SAS inhibits the cystine/glutamate antiporter system Xc ^−^ [ 5, 6], which is essential for cystine uptake and intracellular glutathione (GSH) synthesis [7]. By depleting GSH, SAS disrupts the cellular redox balance and triggers oxidative stress, culminating in ferroptotic cell death. Studies have shown that SAS can induce ferroptosis in several cell lines [ 8– 15], and SAS can increase the efficacy of chemotherapeutic agents by increasing oxidative damage [5].

Ferroptosis is a form of regulated cell death often associated with the accumulation of reactive oxygen species (ROS) [16]. Unlike apoptosis or necrosis, ferroptosis has notable morphological and biochemical features, such as reduced mitochondrial size, increased membrane density, and GSH deficiency-associated oxidative damage to cellular lipids [17]. Central to this process is the depletion of cellular GSH and inactivation of glutathione peroxidase 4 (GPX4), and these changes disrupt a cell’s ability to neutralize reactive lipid peroxides (lipid-ROS), subsequently leading to cell death [18] .

Protein disulfide isomerase (PDI or PDIA1) is a well-known member of the thioredoxin superfamily and is primarily localized in the endoplasmic reticulum [19]. Functionally, PDI serves as a dithiol/disulfide oxidoreductase and facilitates protein folding by catalyzing the isomerization of the intra- and intermolecular disulfide bonds [20]. Recent studies from our laboratory have shown that PDI is involved in mediating chemically-induced, glutathione (GSH) depletion-associated ferroptosis [ 21– 23]. Specifically, GSH depletion leads to PDI oxidation and activates its catalytic activity for nitric oxide synthase (NOS) dimerization via disulfide bond formation, which subsequently results in the buildup of cellular nitric oxide (NO) and ROS/lipid-ROS and mitochondrial ROS and ultimately ferroptotic cell death [ 21– 23]. Notably, the inhibition of PDI function has been shown to block NOS dimerization and NO accumulation and thus effectively rescues cells from chemically induced ferroptosis [ 21– 23]. These findings position PDI as a pivotal upstream mediator of ferroptosis, linking GSH depletion to oxidative cell death pathways.

Recently, we showed that PDI is involved in mediating SAS-induced ferroptosis in immortalized HT22 mouse hippocampal neurons as an *in vitro* model [15]. In the present study, we sought to further characterize the detailed cellular and biochemical mechanisms of SAS-induced ferroptotic cell death in two rat cell lines, namely, H9C2 cardiomyocytes and BRL-3A hepatocytes. These two cell lines were selected as *in vitro* models for study because earlier studies have shown that they are sensitive to erastin- and RSL3-induced ferroptosis in culture [ 22– 27]. Moreover, we recently showed that PDI is involved in mediating erastin-induced ferroptotic cell death in BRL-3A rat hepatocytes [25]. Confirming our recent observations [15], the results of our present study further demonstrate that PDI plays a pivotal role in mediating SAS-induced ferroptosis in both H9C2 cardiomyocytes and BRL-3A hepatocytes, which involves PDI-mediated NOS dimerization, accumulation of NO, ROS and lipid-ROS, and ultimately ferroptotic cell death.

## Materials and Methods

### Chemicals and reagents

Sulfasalazine (SAS, #HY-14655), BAZ and ferrostatin-1 (Fer-1, #HY-100579) were obtained from MedChemExpress (Monmouth Junction, USA); *N* -acetyl- *L*-cysteine (NAC, #A8199), cystamine dihydrochloride (cystamine, #C121509), and thiazolyl blue tetrazolium bromide (MTT, #T818538) were obtained from MACKLIN (Shanghai, China); carboxy-PTIO (cPTIO, #S1547), diaminofluorescein-FM diacetate (DAF-FM-DA, #S0019) and 2′,7′-dichlorodihydrofluorescein diacetate (DCFH-DA, #S0033S) were obtained from Beyotime Biotechnology (Shanghai, China); BODIPY-581/591-C11 (#D3861) was obtained from Thermo Fisher Scientific (Waltham, USA); and EN460 (#M07515) was obtained from BioLab (Beijing, China).

The anti-iNOS antibody (#ab15323) was obtained from Abcam (Cambridge, UK), the anti-PDI antibody (#3501S) and anti-β-actin (#4970) antibodies were purchased from Cell Signaling Technology (Boston, USA), and the goat anti-mouse IgG and goat anti-rabbit IgG conjugated to horseradish peroxidase (#7074S) were obtained from Santa Cruz Biotechnology (Santa Cruz, USA) and used as secondary antibodies. Most of the other chemicals were obtained from Sigma-Aldrich (St Louis, USA).

### Cell culture and viability assay

Rat H9C2 cardiomyocytes and BRL-3A hepatocytes were obtained from the Cell Bank of the Chinese Academy of Sciences (Shanghai, China) and were maintained in DMEM supplemented with 10% ( *v*/ *v*) fetal bovine serum (FBS; Thermo Fisher Scientific) and antibiotics (100 U/mL penicillin+100 μg/mL streptomycin; Sigma-Aldrich) at 37°C under 5% CO _2_. The cells were passaged or used in experiments upon reaching approximately 80% confluence, and they were usually passaged under 25 passages. Authentication was performed by STR profiling and routine mycoplasma testing.

The MTT cell viability assay was described in our recent study [15]. Briefly, cells seeded in 96-well plates (2000 cells/well) were treated with drugs as indicated, and MTT (0.5 mg/mL) was added to each well and incubated for 3 h at 37°C under 5% CO _2_. DMSO was subsequently added to dissolve the MTT formazan, and the absorbance was measured at 560 nm wavelength.

### siRNA transfection

The procedures of siRNA transfection of cultured cells were described in our earlier study [ 24, 25 ]. Briefly, 24 h after seeding, siRNAs (60 nM) for targeted genes ( *PDI* or *iNOS*) were transfected using Lipofectamine RNA iMAX (Invitrogen, Carlsbad, USA). Forty-eight hours after siRNA transfection, the cells were treated with the selected drugs and subsequently processed for cell viability determination, immunoblotting and fluorescence imaging. The sequences of iNOS-siRNAs and PDI-siRNAs are shown in Supplementary Table S1.

### Measurement of cellular NO and ROS levels by fluorescence microscopy

The measurement procedures were described in our earlier studies [ 15, 24, 25]. The cells were plated in 24-well plates at a density of 5 × 10 ^4^ per well and then given different drug treatments as indicated. For fluorescence microscopy of cellular NO and total ROS, the cells were incubated with 5 μM DAF-FM-DA and DCFH-DA, respectively, in 200 μL of DMEM (free of serum and phenol red) for 20 min at 37°C. After washing with HBSS three times, fluorescence images were captured with an AXIO fluorescence microscope (Carl Zeiss, Wetzlar, Germany).

### Confocal microscopy

For visualization of the subcellular distribution of cellular lipid-ROS, cells were seeded at a density of 5 × 10 ^4^ per well on coverslips placed inside 24-well plates. Twenty-four hours later, the cells were treated with drugs as indicated. Coverslips were washed in HBSS, incubated in HBSS containing 5 μM BODIPY-581/591-C11 for 20 min at 37°C, and then mounted on microscope slides for visualization as described earlier [ 15, 24, 25].

### Flow cytometry

For flow cytometric analysis of the cellular levels of NO, ROS and lipid-ROS, the cells were plated in 6-well plates at a density of 1.5 × 10 ^5^ cells/well for 24 h before treatment with different drugs. Afterwards, the cells were trypsinized, collected and suspended in phosphate-buffered saline (PBS). The cells were then centrifuged, and the resulting cell pellets were resuspended in DMEM (free of phenol red and serum) containing 5 μM DAF-FM-DA, DCFH-DA or BODIPY-581/591-C11. After a 20-min incubation at 37°C, the cells were washed three times with HBSS and measured via a flow cytometer (Beckman Coulter, Brea, USA). The data were analyzed using the FlowJo software (FlowJo, LLC, Ashland, USA).

### Western blot analysis

Following drug treatment, the cells were collected by trypsinization and centrifugation and then lysed on ice for 30 min in RIPA buffer. For total iNOS protein analysis, samples were heated at 95°C for 5 min with reducing buffer before being loaded onto the electrophoresis gel. To analyze the monomeric and dimeric forms of iNOS, the samples were prepared in a non-reducing buffer and were not heated, and the temperature of the gel was maintained below 15°C during electrophoresis. The proteins were separated using 10% agarose gel (for total iNOS) or 6% agarose gel (for monomeric and dimeric iNOS) and then transferred to PVDF membranes. Other procedures for western blot analysis were the same as those described in our recent studies [ 15, 24, 25 ]. The experiments were repeated to confirm the observations, and representative blots from one representative experiment are shown.

### Statistical analysis

In this study, the quantitative experiments were repeated multiple times to confirm the experimental observations. Statistical analyses were carried out using one-way ANOVA followed by Dunnett′s *post hoc* tests for multiple comparisons (GraphPad Prism 7.0 software; GraphPad Software, La Jolla, USA). Data are presented as the mean ± standard deviation (SD), which are typically obtained from a representative experiment with multiple replicates. *P* < 0.05 (* or ^#^) and *P* < 0.01 (** or ^##^) indicate statistically significant and very significant differences, respectively. In most cases, * and ** denote comparisons for statistical significance between the control group (cells treated with vehicle only) and the cells treated with SAS, whereas ^#^ and ^##^ denote comparisons between the cells treated with SAS and the cells treated with SAS + another compound. For western blot quantification, one representative dataset is shown.

## Results

### Induction of ferroptotic cell death by SAS in H9C2 and BRL-3A cells

**Figure 1:**
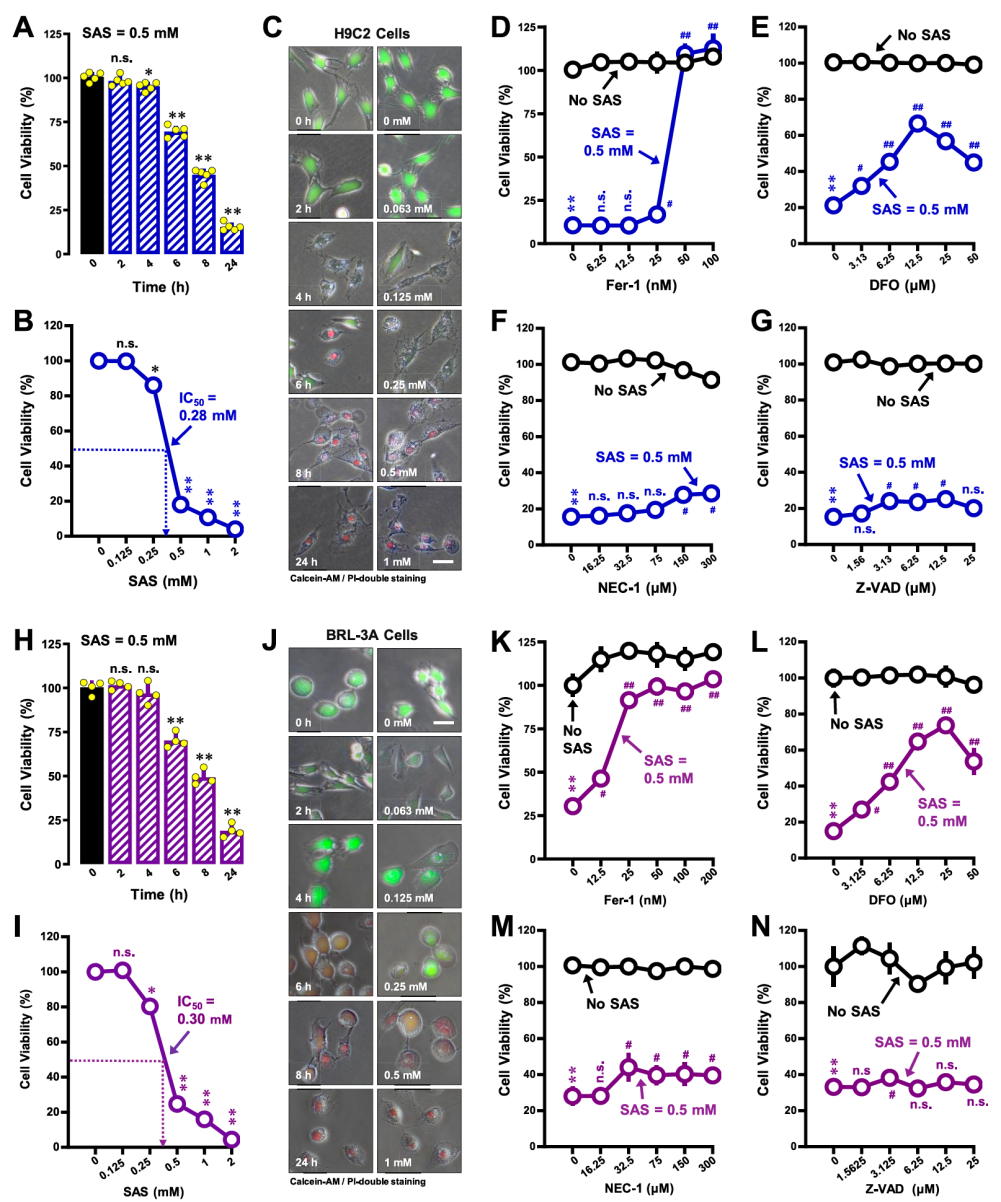
Figure 1 Induction of ferroptotic cell death by SAS in H9C2 and BRL-3A cells (A–C,H–J) Time- and dose-dependent induction of cell death by SAS in H9C2 (A–C) and BRL-3A (H–J) cells. The cells were treated with different concentrations of SAS for 24 h or 0.4 mM SAS for 0, 2, 4, 6, 8 or 24 h. Cell viability was determined by MTT assay (A, B, H, I; n=5) or fluorescence microscopy (C,J) following calcein-AM/PI staining (20×; scale bar=100 μm). (D–G) Effects of Fer-1 (D), DFO (E), NEC-1 (F) and z-VAD-FMK (G) on SAS-induced death in H9C2 cells. The cells were treated with SAS (0.5 mM) ± Fer-1, DFO, z-VAD-FMK or NEC-1 for 24 h and then subjected to MTT assay (n = 5). (K–N) Effects of Fer-1 (K), DFO (L), NEC-1 (M) and z-VAD-FMK (N) on SAS-induced death in BRL-3A cells. The cells were treated with SAS (0.5 mM) ± Fer-1, DFO, z-VAD-FMK or NEC-1 for 24 h and then subjected to MTT assay (n = 5). The quantitative data are presented as the mean ± SD. * or #P < 0.05; ** or ## P < 0.01; n.s., not significant.

The cytotoxicity of SAS in H9C2 rat cardiomyocytes was evaluated by exposing the cells to increasing concentrations (0.125, 0.25, 0.5, 1, and 2 mM) of SAS over a 24-h period, and cell viability was assessed using the MTT assay. SAS elicited cytotoxicity in H9C2 cells in a time- and concentration-dependent manner ( Figure 1A,B), with an IC _50_ of ~0.28 mM. SAS-induced cell death was further verified with calcein-AM/PI double staining ( Figure 1C). Morphological changes in cells resembling ferroptosis were noted at 6-8 h after SAS exposure ( Supplementary Figure S1A). At 0.5 mM SAS, the cell survival rate decreased to ~20%, making this concentration ideal for further testing the protective effects of selected compounds against SAS-induced cytotoxicity.

To ascertain whether SAS-induced cell death in H9C2 cells qualifies as ferroptosis, we investigated the protective effects of Fer-1, deferoxamine (DFO) and necrostatin-1 (Nec-1) on SAS-induced cytotoxicity. Fer-1, a known antioxidant for lipid-ROS and a prototypical ferroptosis inhibitor [28], completely rescued SAS-induced cell death when it was present at 50-100 μM ( Figure 1D). Similarly, DFO, an iron chelator known for its ferroptosis-rescuing activity [ 29, 30] also provided substantial protection ( Figure 1E). In comparison, necrostatin-1 (NEC-1), an inhibitor of necrosis [ 31, 32], and z-VAD-FMK, a pancaspase inhibitor [ 33, 34], did not exhibit similar protection against SAS-induced cell death ( Figure 1F,G).

SAS-induced ferroptotic cell death was also evaluated in BRL-3A rat hepatocytes. SAS exhibited time- and concentration-dependent cytotoxicity in these cells ( Figure 1H,I for the MTT assay; Figure 1J for calcein-AM/PI staining), with an IC _50_ of ~0.30 mM. Morphological changes consistent with ferroptosis were observed approximately 8 h after SAS exposure ( Supplementary Figure S1B). Consistent with the induction of ferroptosis by SAS in BRL-3A cells, Fer-1 and DFO mitigated SAS-induced cell death ( Figure 1K,L), but NEC-1 and z-VAD-FMK did not exert similar protection against SAS-induced cell death ( Figure 1M,N). Collectively, these findings strongly indicate that SAS triggers a form of cell death that closely aligns with ferroptosis in both H9C2 and BRL-3A cells.

### Effects of SAS on cellular NO, ROS and lipid-ROS accumulation

**Figure 2:**
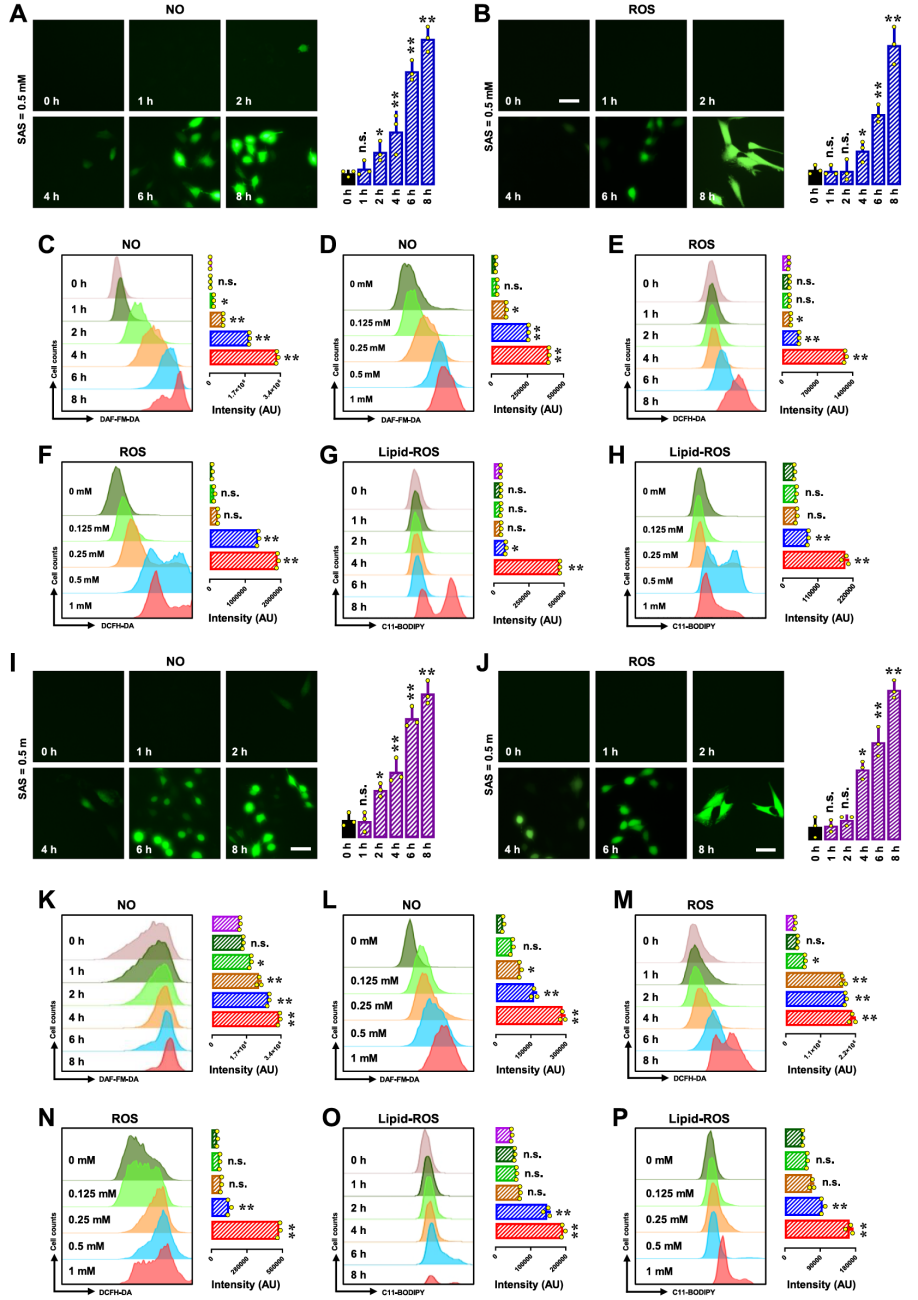
Figure 2 Time- and concentration-dependent changes in the cellular NO, ROS and lipid-ROS levels in SAS-treated H9C2 and BRL-3A cells (A‒H) Time- and concentration-dependent SAS-induced accumulation of NO (A,C,D), ROS (B,E,F) and lipid-ROS (G,H) in H9C2 cells. The cells were treated with 0.5 mM SAS for varying durations as specified (for time dependency) or with different concentrations of SAS as indicated for 8 h (for concentration dependency). Then, the cells were subjected to fluorescence microscopy (scale bar = 100 μm) or analytical flow cytometry. For the fluorescence microscopy data (A,B), the quantitative intensity values are shown in the right panels; similarly, the intensity values for the flow cytometry data (C–H) are also shown in the right panels (n = 3). (I‒P) Time- and concentration-dependent SAS-induced accumulation of NO (I,K,L), ROS (J,M,N) and lipid-ROS (O,P) in BRL-3A cells. The treatment conditions, measurements and figure annotations are the same as those described above for panels (A‒H). The quantitative data are presented as the mean ± SD. *P < 0.05; **P < 0.01 vs the control group; n.s., not significant.

Our recent research revealed that the accumulation of NO, ROS and lipid-ROS plays a pivotal role in the induction of ferroptotic cell death by erastin [ 22, 23] and RSL3 [24]. To explore whether similar accumulations also contribute to SAS-induced ferroptosis, we first investigated the time- and concentration-dependent changes in the cellular NO, ROS and lipid-ROS levels in H9C2 cells. A small increase in the cellular NO level (using DAF-FM-DA as a probe) was observed between 2 and 4 h following SAS exposure, with a more pronounced increase occurring between 6 and 8 h ( Figure 2A,C). As expected, SAS also elicited a concentration-dependent increase in the cellular NO level ( Figure 2D).

Similarly, SAS exposure caused a time-dependent increase in cellular ROS levels (using DCFH-DA as a probe; Figure 2B,E). An initial small increase in cellular ROS was detected at 4–6 h after exposure, peaking at 8 h ( Figure 2B,E). Additionally, there was a concentration-dependent increase in ROS levels in SAS-treated cells ( Figure 2F). The increase in the cellular ROS level appeared to be behind the increase in the cellular NO level.

Lipid-ROS accumulation is a defining feature of chemically-induced ferroptosis [ 35, 36]. In this study, we examined changes in the lipid-ROS levels in SAS-treated H9C2 cells (using C11-BODIPY as a probe). We observed that SAS elicited time- and concentration-dependent increases in the cellular lipid-ROS levels in these cells ( Figure 2G,H).

We also investigated the time- and concentration-dependent changes in cellular NO, ROS and lipid-ROS levels in BRL-3A cells ( Figure 2I–P). SAS caused a modest increase in NO levels between 2 and 4 h following SAS exposure, and a more pronounced increase occurred between 6 and 8 h ( Figure 2I,K). As expected, SAS elicited a concentration-dependent increase in the cellular NO level ( Figure 2L). Similarly, SAS exposure also elicited time- and concentration-dependent increases in cellular ROS levels ( Figure 2J,M for time dependency; Figure 2N for concentration dependency) and cellular lipid-ROS levels ( Figure 2O,P) in BRL-3A cells.

Collectively, these results demonstrate that SAS elicits sequential and time-dependent increases in the cellular NO, ROS and lipid-ROS levels in both H9C2 and BRL-3A cells. It is evident that SAS-induced NO accumulation precedes the accumulation of ROS and lipid-ROS in these cells.

### Effects of NO, ROS and lipid-ROS scavengers on SAS-induced cell death

To determine the role of cellular NO, ROS and lipid-ROS accumulation in SAS-induced ferroptosis in H9C2 cells, representative scavengers of NO, ROS and lipid-ROS were used to evaluate their effects on SAS-induced ferroptosis by measuring changes in cell viability and cellular NO, ROS and lipid-ROS levels.

#### cPTIO

**Figure 3:**
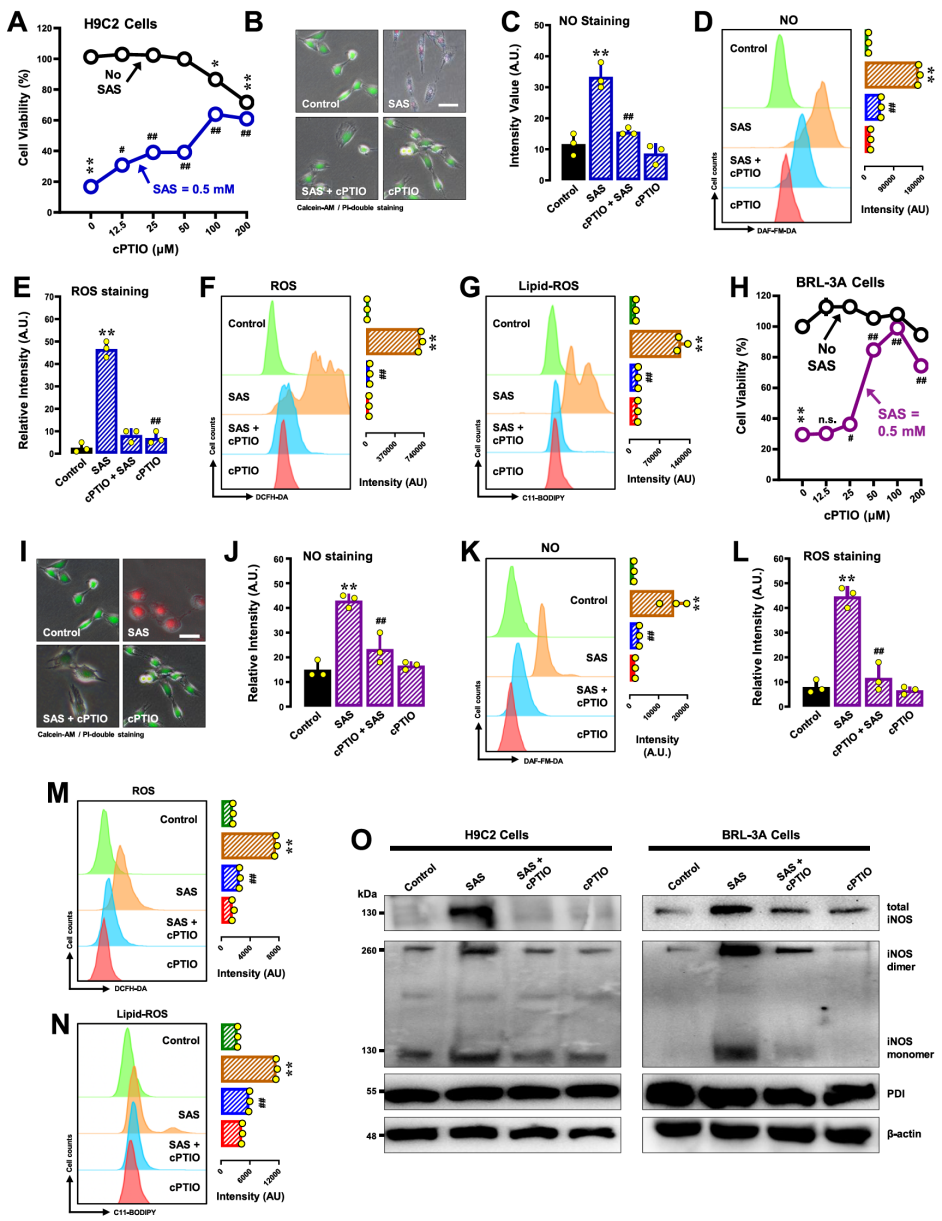
Figure 3 Effects of cPTIO on SAS-induced cytotoxicity; NO, ROS and lipid-ROS accumulation; and iNOS dimerization in H9C2 and BRL-3A cells (A,B,H,I) Protective effect of cPTIO on SAS-induced cytotoxicity in H9C2 (A,B) and BRL-3A (H,I) cells. In (A and H), the cells were treated with SAS (0.4 mM) ± cPTIO (12.5, 25, 50, 100, and 200 μM) for 24 h and then subjected to MTT assay (n = 4). In (B and I), cells were treated with SAS (0.5 mM) ± cPTIO (50 μM) for 24 h and then subjected to fluorescence microscopy following calcein-AM/PI staining (green for live cells and red for dead cells; scale bar = 100 μm). (C–G) Abrogation by cPTIO of SAS-induced accumulation of cellular NO (C,D), ROS (E,F) and lipid-ROS (G) in H9C2 cells. The cells were treated with SAS (0.5 mM) ± cPTIO (100 μM) for 8 h and then subjected to fluorescence microscopy (C,E) and analytical flow cytometry (D–G). For the fluorescence microscopy data in (C,E), only the quantitative intensity values are shown (n = 3). For flow cytometry data (D,F,G), the intensity values are shown in the right panels ( n = 3). (J–N) Abrogation by cPTIO of SAS-induced accumulation of cellular NO (J, K), ROS (L, M) and lipid-ROS (N) in BRL-3A cells. The cells were treated with SAS (0.5 mM) ± cPTIO (100 μM) for 8 h and then subjected to fluorescence microscopy (J,L) and analytical flow cytometry (K,M,N). For the fluorescence microscopy data in (J and L), only the quantitative intensity values are shown (n = 3). For the flow cytometry data (K,M,N), the corresponding intensity values are shown in the right panels (n = 3). (O) Effects of cPTIO on SAS-induced changes in total, monomeric and dimeric iNOS protein levels in H9C2 and BRL-3A cells (western blot analysis). The cells were treated with SAS (0.5 mM) ± cPTIO (100 μM) for 8 h, after which the levels of total, monomeric and dimeric iNOS proteins and PDI were determined by western blot analysis. β-Actin was used as a loading control. The quantitative data are presented as the mean ± SD. * or # P < 0.05; ** or ## P < 0.01; n.s., not significant.

cPTIO is an NO scavenger commonly used in cell culture studies [37]. To explore whether NO accumulation leads to ROS/lipid-ROS buildup and subsequent cell death, we examined the impact of cPTIO on SAS-induced ferroptosis in H9C2 cells. Our findings revealed that cPTIO partially mitigated SAS-induced cell death ( Figure 3A for the MTT assay; Figure 3B for calcein-AM/PI double staining). The modest cytoprotective effect of cPTIO may be related to its own cytotoxicity, as this chemical can produce cytotoxic NO _2_ during its scavenging of NO [38]. We also investigated the effects of cPTIO on the accumulation of cellular NO, ROS and lipid-ROS. SAS-induced NO accumulation was partially reduced by co-treatment with 100 μM cPTIO ( Figure 3C,D); in comparison, the SAS-induced accumulation of cellular ROS ( Figure 3E,F) and lipid-induced ROS ( Figure 3G) was more strongly reduced by cPTIO. Joint treatment of cells with cPTIO also abrogated the SAS-induced increases in the levels of the total, dimeric and monomeric forms of the NOS protein ( Figure 3O, left panel).

Similar analyses were performed in BRL-3A cells. We found that cPTIO partially mitigated SAS-induced cell death ( Figure 3H for the MTT assay; Figure 3I for calcein-AM/PI staining). SAS-induced NO accumulation was partially reduced by cotreatment with 100 μM cPTIO ( Figure 3J,K). Interestingly, SAS-induced increases in cellular ROS ( Figure 3L,M) and lipid-ROS ( Figure 3N) were more significantly reduced by cPTIO. cPTIO also reduced SAS-induced increases in the total, dimeric and monomeric forms of the iNOS protein ( Figure 3O, right panel).

#### NAC

**Figure 4:**
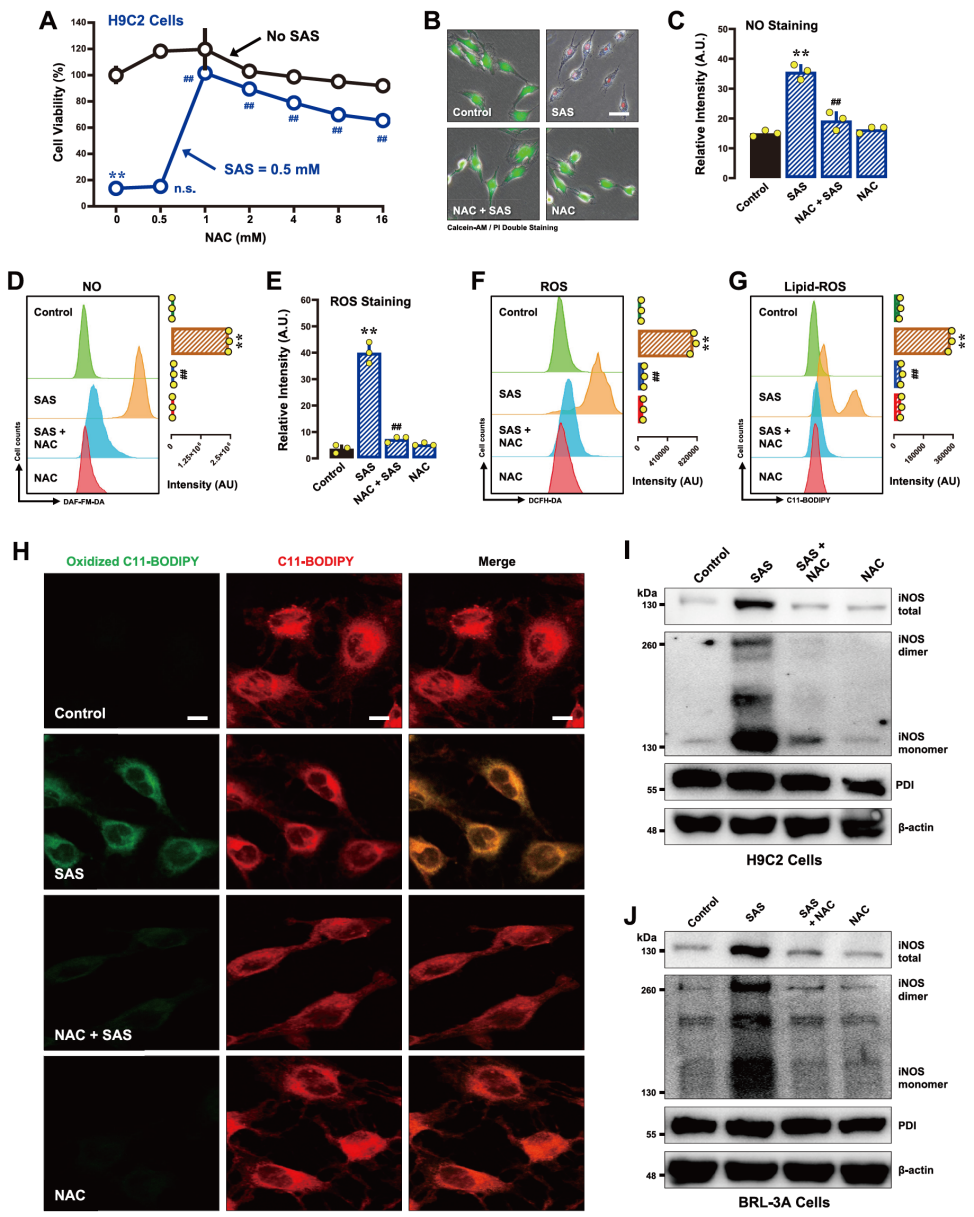
Figure 4 Effect of NAC on SAS-induced ferroptosis and the accumulation of NO, ROS and lipid-ROS in H9C2 and BRL-3A cells (A,B) Protective effect of NAC against SAS-induced cytotoxicity in H9C2 cells. In (A), the cells were treated with SAS (0.5 mM) ± NAC (0.5, 1, 2, 4, 8 or 16 mM) for 24 h and then subjected to MTT assay (n = 4). In (B), cells were treated with SAS (0.5 mM) ± NAC (1 mM) for 24 h and then subjected to fluorescence microscopy following calcein-AM/PI staining (green for live cells and red for dead cells; scale bar = 100 μm). (C–H) Abrogation by NAC of SAS-induced accumulation of cellular NO (C,D), ROS (E,F) and lipid-ROS (G,H) in H9C2 cells. The cells were treated with SAS (0.5 mM) ± NAC (1 mM) for 8 h and then subjected to fluorescence microscopy (C,E), flow cytometry (D,F,G) and confocal microscopy (H; scale bar=100 μm). For the fluorescence microscopy data in (C,E), only the quantitative intensity values are shown ( n = 3). For the flow cytometry data (D,F,G), the left panels are the histograms, and the right panels are the quantitative values (n = 3). (I,J) Effects of NAC on SAS-induced changes in total, monomeric and dimeric iNOS protein levels in H9C2 (I) and BRL-3A (J) cells. The cells were treated with SAS (0.5 mM) ± NAC (1 mM) for 8 h, after which the levels of total, monomeric and dimeric iNOS proteins and PDI were determined by western blot analysis. β-Actin was used as a loading control. The quantitative data are presented as the mean ± SD. ** or ## P < 0.01; n.s., not significant.

*n*-Acetyl- *l*-cysteine (NAC) is an antioxidant known for its robust protective effect against oxidative stress-associated cytotoxicity [ 39, 40 ]. In H9C2 cells, joint treatment with 1 mM NAC strongly prevented SAS-induced cell death, achieving nearly complete cytoprotection ( Figure 4A,B). NAC also abrogated SAS-induced accumulation of NO ( Figure 4C,D), ROS ( Figure 4E) and lipid-ROS ( Figure 4G,H) in these cells, illustrating its broad-spectrum antioxidant capacity.

In BRL-3A cells, NAC also prevented SAS-induced cell death ( Supplementary Figure S2A for the MTT assay; Supplementary Figure S2B for calcein-AM/PI staining). Moreover, NAC strongly mitigated the SAS-induced accumulation of cellular NO ( Supplementary Figure S2C), ROS ( Supplementary Figure S2D) and lipid-ROS ( Supplementary Figure S2E,F).

In addition to its effects on oxidative markers, joint treatment with NAC suppressed SAS-induced increases in the levels of total iNOS and its monomeric and dimeric forms ( Figure 4I for H9C2 cells; Figure 4J for BRL-3A cells).

#### Fer-1

**Figure 5:**
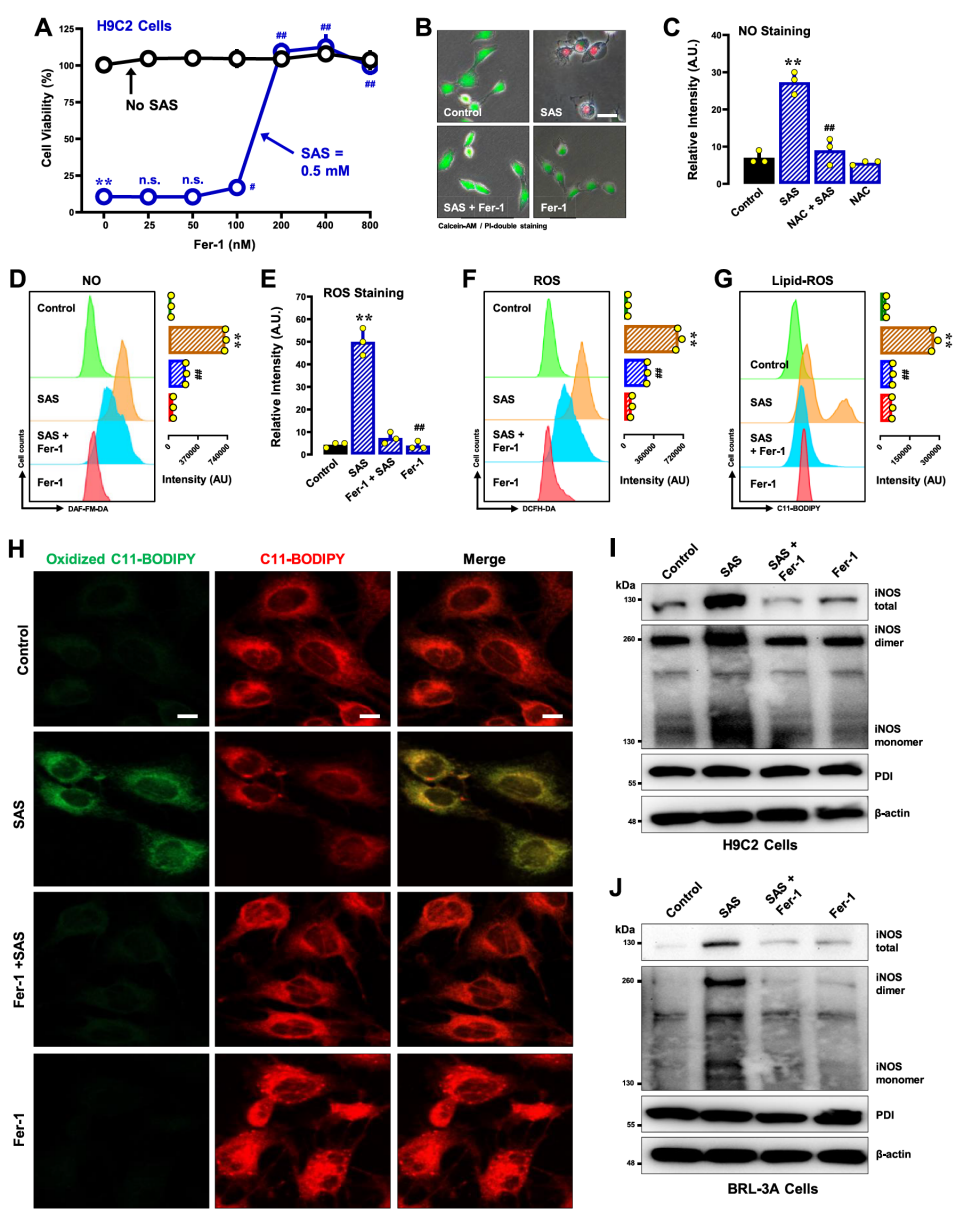
Figure 5 Effect of Fer-1 on SAS-induced ferroptosis and NO, ROS and lipid-ROS accumulation in H9C2 and BRL-3A cells (A,B) Protective effect of NAC against SAS-induced cytotoxicity. In (A), H9C2 cells were treated with SAS (0.5 mM) ± Fer-1 (25, 50, 100, 200, 400 and 800 nM) for 24 h, and then, cell viability was determined by MTT assay (n = 4). In (B), cells were treated with SAS (0.5 mM) ± Fer-1 (200 nM) for 24 h, and then, fluorescence images of calcein-AM/PI-stained cells were captured (green for live cells and red for dead cells; scale bar = 100 μm). (C–H) Abrogation by Fer-1 of SAS-induced accumulation of cellular NO (C,D), ROS (E,F) and lipid-ROS (G,H) in H9C2 cells. The cells were treated with SAS (0.5 mM) ± Fer-1 (200 nM) for 8 h and then subjected to fluorescence microscopy (C,E), flow cytometry (D,F,G) and confocal microscopy (H; scale bar = 100 μm). For the fluorescence microscopy data in (C and E), only the quantitative intensity values are shown (n=3). For the flow cytometry data (D,F,G), the left panels are the histograms, and the right panels are the quantitative values (n = 3). (I,J) Effects of Fer-1 on SAS-induced changes in total, monomeric and dimeric iNOS protein levels in H9C2 (I) and BRL-3A (J) cells. The cells were treated with SAS (0.5 mM) ± Fer-1 (200 nM) for 8 h, after which the levels of total, monomeric and dimeric iNOS proteins and PDI were determined by western blot analysis. β-Actin was used as a loading control. The quantitative data are presented as the mean ± SD. * or #P < 0.05; ** or ##P < 0.01; n.s., not significant.

Fer-1 exhibited robust protection against SAS-induced ferroptosis at a low concentration (50 nM), exhibiting nearly 100% protection in H9C2 cells ( Figure 5A for the MTT assay; Figure 5B for calcein-AM/PI double staining). Fer-1 effectively abrogated SAS-induced accumulation of cellular NO, ROS and lipid-ROS ( Figure 5C,D for NO; Figure 5E,F for ROS; Figure 5G,H for lipid-ROS).

Similarly, Fer-1 strongly protected BRL-3A cells against SAS-induced ferroptosis ( Supplementary Figure S3A for the MTT assay; Supplementary Figure S3B for calcein-AM/PI staining). Fer-1 abrogated SAS-induced accumulation of cellular NO, ROS and lipid-ROS ( Supplementary Figure S3C for NO; Supplementary Figure S3D for ROS; Supplementary Figure S3E,F for lipid-ROS). Additionally, Fer-1 abrogated SAS-induced increases in total cellular iNOS protein as well as its mononer and dimer ( Figure 5I for H9C2 cells; Figure 5J for BRL-3A cells).

#### DFO

Deferoxamine (DFO) is an iron chelator with known ferroptosis-rescuing properties [ 29, 30 ]. DFO was shown to significantly protect against SAS-induced cytotoxicity, as shown by the MTT assay ( Supplementary Figure S4A for H9C2 cells; Supplementary Figure S4F for BRL-3A cells) and calcein-AM double staining ( Supplementary Figure S4B for H9C2 cells; Supplementary Figure S4G for BRL-3A cells). Furthermore, DFO effectively abrogated SAS-induced accumulation of cellular NO, ROS and lipid-ROS ( Supplementary Figure S4C–E for H9C2 cells; Supplementary Figure S4H–J for BRL-3A cells).

Trolox, a compound known for its potent ROS-scavenging activity and limited NO-scavenging ability [41], provided nearly complete protection against SAS-induced cytotoxicity in H9C2 and BRL-3A cells ( Supplementary Figure S5A for H9C2 cells; Supplementary Figure S5B for BRL-3A cells). In contrast, MitoTEMPO, a mitochondria-targeting antioxidant and superoxide dismutase mimetic that converts mitochondrial superoxide anions to H _2_O _2_ [42], did not show any protective effect against SAS-induced cytotoxicity in these two cell lines ( Supplementary Figure S5C for H9C2 cells; Supplementary Figure S5D for BRL-3A cells). In contrast, TEMPO partially protected against SAS-induced cytotoxicity ( Supplementary Figure S5E for H9C2 cells; Supplementary Figure S5F for BRL-3A cells).

Together, these findings indicate that SAS induces the accumulation of cellular NO, ROS and lipid-ROS, which collectively mediate the induction of oxidative ferroptosis in both H9C2 and BRL-3A cells.

### Effect of SNP on SAS-induced ferroptotic cell death

To provide additional support for the hypothesis that SAS-induced NO accumulation is a key event that subsequently leads to ROS/lipid-ROS accumulation, we investigated the effects of sodium nitroprusside (SNP), a known NO donor [43], on ROS and lipid-ROS accumulation as well as on ferroptotic cell death in H9C2 and BRL-3A cells that were jointly treated with SAS and SNP.

**Figure 6:**
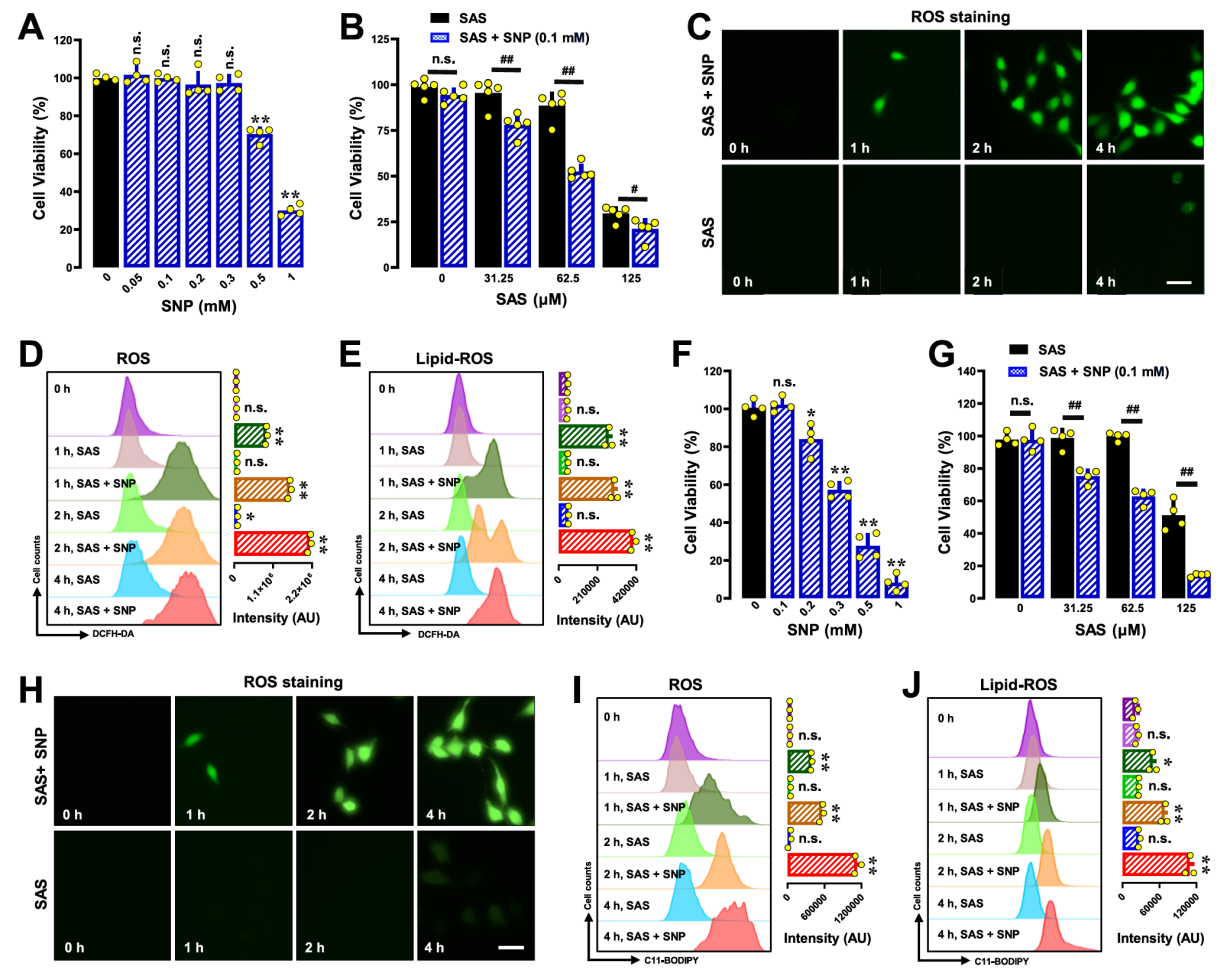
Figure 6 Effect of SNP on SAS-induced ferroptotic H9C2 and BRL-3A cell death (A,C) Concentration-dependent cytotoxicity of SNP in H9C2 (A) and BRL-3A (C) cells (MTT assay, n = 4). (F,G) Enhancement of SAS-induced ferroptosis by SNP in H9C2 (F) and BRL-3A (G) cells. The cells were exposed to increasing concentrations of SAS ± SNP (0.1 mM) for 24 h, and cell viability was assessed using MTT assay (n = 4). (C–E,H–J) Time-dependent accumulation of cellular ROS (C,D,H,I) and lipid-ROS (E,J) in H9C2 and BRL-3A cells. The cells were treated with SAS (0.5 mM) ± SNP (0.1 mM) for 1, 2 or 4 h and then subjected to fluorescence microscopy (C,H) or flow cytometry analysis (D,E,I,J). For the flow cytometry data, the left panels are histograms, and the right panels are the corresponding quantitative values (n = 3). The quantitative data are presented as the mean ± SD. ** or ## P < 0.01; n.s., not significant.

Using the MTT assay, we found that 300 μM SNP alone produced minimal cytotoxicity in H9C2 cells ( Figure 6A). However, co-treatment with 100 μM SNP significantly enhanced SAS-induced cytotoxicity ( Figure 6B). Our earlier studies revealed that SNP alone can lead to the accumulation of ROS and lipid-ROS in cells [ 22– 24 ]. In this study, we found that combined treatment of H9C2 cells with SNP (100 μM) + SAS markedly accelerated the accumulation of cellular ROS ( Figure 6C,D) and lipid-ROS ( Figure 6E). Similar observations were also made in BRL-3A cells. While SNP at 200 μM produced minimal cytotoxicity in these cells ( Figure 6F), combined treatment with 100 μM SNP significantly enhanced SAS-induced cytotoxicity ( Figure 6G). In addition, combined treatment of cells with SNP (100 μM) markedly accelerated the SAS-induced accumulation of cellular ROS ( Figure 6H,I) and lipid-ROS ( Figure 6J). These findings suggest that elevated cellular NO levels can amplify SAS-induced ferroptosis by accelerating the accumulation of cellular ROS/lipid-ROS.

### Effect of SAS on iNOS protein levels and dimerization

**Figure 7:**
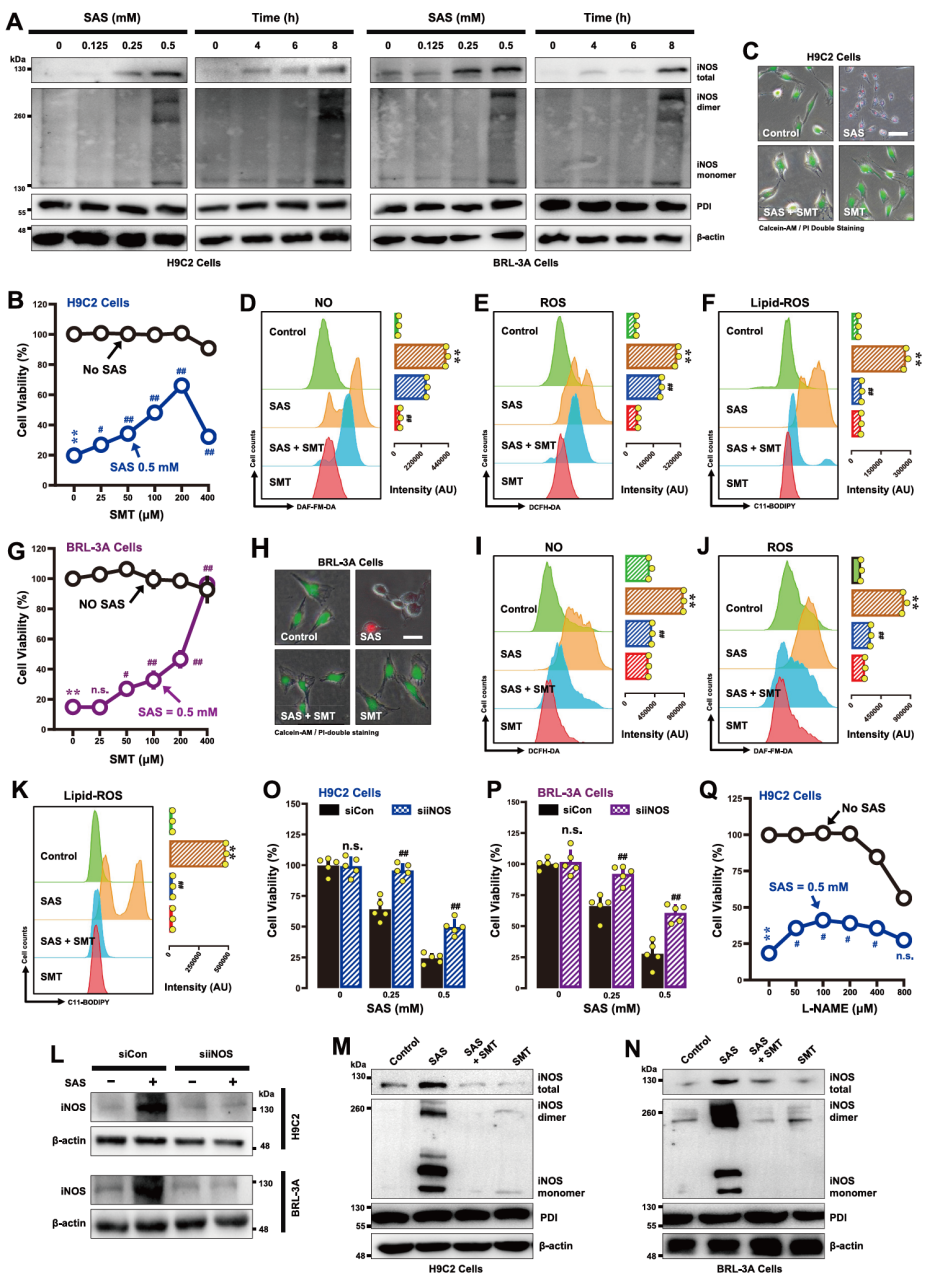
Figure 7 Effect of SAS on iNOS upregulation and dimer formation in H9C2 and BRL-3A cells (A) Concentration- and time-dependent effects of SAS on the total, monomeric and dimeric iNOS protein levels (western blot analysis) in H9C2 (A) and BRL-3A (A) cells. The cells were treated with different concentrations of SAS for 8 h or with 0.5 mM SAS for different durations, after which the total, monomeric and dimeric iNOS protein levels were determined by western blot analysis. Cellular PDI and β-actin levels were also determined for comparison. (B,C,G,H) Protective effect of SMT against SAS-induced cytotoxicity in H9C2 (B,C) and BRL-3A cells (G,H). In (B,G), cells were exposed to SAS (0.5 mM) ± SMT (25, 50, 100, 200 and 400 μM) for 24 h and then subjected to MTT assay (n = 5). In (C,H), the cells were treated with SAS (0.5 mM) ± SMT (200 μM for H9C2 cells/400 μM for BRL-3A cells) for 24 h and then subjected to fluorescence microscopy following calcein-AM/PI staining (C,H; green color for live cells and red color for dead cells; scale bar = 100 μm). (D-F,I-K) Abrogation by SMT of SAS-induced accumulation of cellular NO (D,I), ROS (E,J) and lipid-ROS (F,K) in H9C2 and BRL-3A cells. The cells were treated with SAS (0.5 mM) ± SMT (200 μM for H9C2 cells/400 μM for BRL-3A cells) for 8 h and then subjected to flow cytometry. For the flow cytometry data, the histograms are shown in the left panels, and the quantitative values are shown in the right panels (n = 3). (L) Effectiveness of iNOS-siRNAs in reducing cellular iNOS protein levels in H9C2 and BRL-3A cells. The cells were transfected with iNOS-siRNAs for 48 h prior to treatment with SAS (0.5 mM) for an additional 8 h. (Q) Effect of L-NAME on SAS-induced cytotoxicity in H9C2 cells. The cells were treated with SAS (0.5 mM) ± L-NAME (25, 50, 100, 200, 400 and 800 μM) for 24 h and then subjected to MTT assay (n = 5). (O,P) Effect of iNOS knockdown on SAS-induced cytotoxicity in H9C2 (O) and BRL-3A (P) cells. The cells were transfected with iNOS-siRNAs 48 h prior to treatment with SAS (0.5 mM) for an additional 24 h, and cell viability was determined by MTT assay (n = 5). (M,N) Effects of SMT on SAS-induced changes in total, monomeric and dimeric iNOS protein levels (western blot analysis). H9C2 (M) and BRL-3A (N) cells were treated with SAS (0.5 mM) ± SMT (200 μM for H9C2 cells/400 μM for BRL-3A cells) for 8 h, and then the total, monomeric and dimeric iNOS proteins in the cells were determined by western blot analysis. The quantitative data are presented as the mean ± SD. * or #P < 0.05; ** or ## P < 0.01; n.s., not significant.

The formation of cellular NO is catalyzed by NOS using *L*-arginine as a substrate [44]. In this study, we evaluated the effects of SAS on protein levels, particularly their dimers, in H9C2 and BRL-3A cells. We found that iNOS and eNOS were expressed in H9C2 cells (iNOS data shown in Figure 7A; eNOS data not shown), but nNOS was essentially not detected (data not shown). The iNOS protein levels were increased in a concentration- and time-dependent manner, with a marked increase observed at 6 h after SAS exposure ( Figure 7A). In comparison, the cellular levels of PDI were not significantly affected by SAS ( Figure 7A).

In BRL-3A cells, only iNOS was detected, and its protein levels were similarly upregulated in a concentration- and time-dependent manner ( Figure 7A). The nNOS and eNOS proteins were largely undetectable in these cells (data not shown). The level of the PDI protein in these cells was not significantly affected by SAS treatment ( Figure 7A).

#### NOS inhibitors

*S*-Methylisothiourea sulfate (SMT) is an iNOS inhibitor [45]. We found that joint treatment of H9C2 cells with SAS+SMT for 24 h resulted in partial protection against SAS-induced ferroptosis ( Figure 7B for the MTT assay; Figure 7C for calcein-AM/PI double staining). SMT partially reduced SAS-induced accumulation of cellular NO ( Figure 7D) and ROS ( Figure 7E) but strongly abrogated SAS-induced lipid-ROS accumulation ( Figure 7F). Similar to the effects observed in H9C2 cells, joint treatment of BRL-3A cells with SMT strongly reversed SAS-induced ferroptosis ( Figure 7G for the MTT assay; Figure 7H for calcein-AM/PI double staining). SMT reduced SAS-induced accumulation of NO ( Figure 7I) and ROS ( Figure 7J) and strongly abrogated SAS-induced lipid-ROS accumulation ( Figure 7K). In addition, SMT abrogated the SAS-induced increase in total iNOS protein and dimer levels ( Figure 7M for H9C2 cells; Figure 7N for BRL-3A cells). For comparison, we also examined the effect of *L*-NAME, an inhibitor of eNOS [ 46, 47 ], on SAS-induced ferroptotic cell death. We found that *L*-NAME elicited very small protection against SAS-induced ferroptosis ( Figure 7Q). Collectively, these results collectively indicate that NO accumulation resulted from the induction and activation (dimerization) of iNOS in SAS-treated cells is involved in mediating SAS-induced ferroptosis in H9C2 and BRL-3A cells.

#### iNOS knockdown

On the basis of the above studies with NOS inhibitors, we next employed the siRNA approach to selectively downregulate iNOS expression to further evaluate its role in mediating SAS-induced ferroptosis in H9C2 and BRL-3A cells. The effectiveness of *iNOS* knockdown was assessed via western blot analysis of total iNOS protein levels ( Figure 7L). Notably, knockdown of *iNOS* resulted in significant protection against SAS-induced cytotoxicity in both H9C2 ( Figure 7O) and BRL-3A cells ( Figure 7P). Notably, while eNOS was also expressed in H9C2 cells, we did not use the siRNA approach to examine its role in SAS-induced ferroptosis, as *L*-NACK, an eNOS inhibitor [ 46, 47 ], elicits only very small protection ( Figure 7Q).

### PDI is an upstream mediator of SAS-induced ferroptosis

#### PDI knockdown

Our earlier studies revealed that the activated PDI ( *i*. *e*., the disulfide bonds in PDI’s catalytic sites are oxidized) mediates chemically induced oxidative ferroptosis through its catalysis of NOS dimerization [ 23, 48 ]. To determine whether activated PDI also mediates NOS dimerization in SAS-treated cells, we first determined whether SAS can directly activate the catalytic activity of PDI via *in vitro* enzymatic assays. As shown in Supplementary Figure S6A,B, the presence of SAS at 250 and 500 μM had little or no effect on the oxidative catalytic activity of PDI. In addition, the presence of SAS at these two concentrations did not affect the reductive activity of PDI ( Supplementary Figure S6C,D).

**Figure 8:**
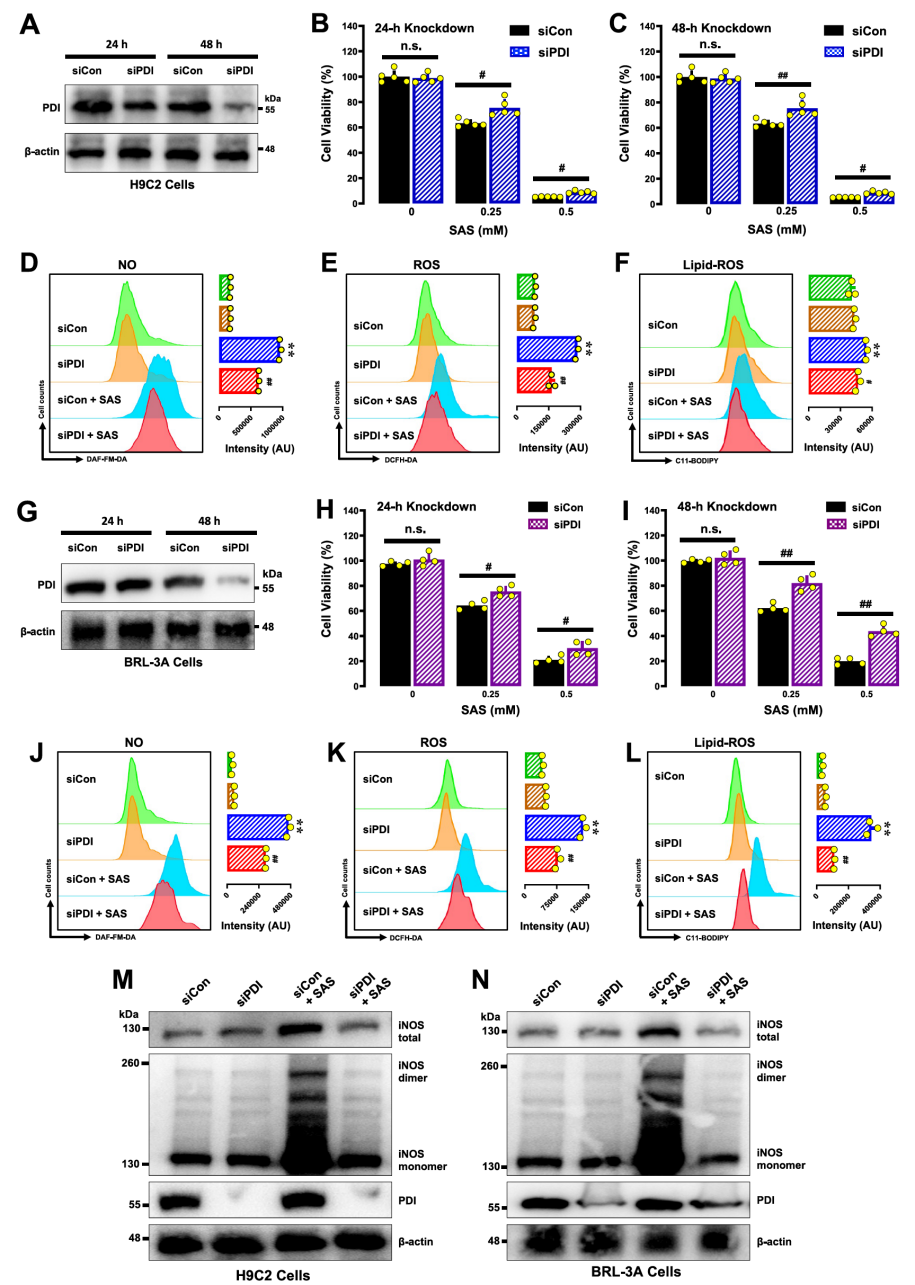
Figure 8 Role of PDI in SAS-induced ferroptosis and the accumulation of NO, ROS and lipid-ROS in H9C2 and BRL-3A cells (A,G) Effectiveness of PDI-siRNAs in reducing the protein level of PDI. H9C2 (A) and BRL-3A (G) cells were transfected with PDI-siRNAs for 24 and 48 h, respectively, and the PDI protein levels were determined by western blot analysis. (B,C,H,I) Protective effect of PDI knockdown on SAS-induced cytotoxicity in H9C2 (B,C) and BRL-3A (H,I) cells. Cells were transfected with PDI-siRNAs for 48 h (C,I) prior to treatment with SAS (0.5 mM) for an additional 24 h (B,H), and cell viability was determined by MTT assay (n = 5). (D–F,J–L) Effects of PDI knockdown on SAS-induced NO (D,J), ROS (E,K) and lipid-ROS (F,L) accumulation in H9C2 and BRL-3A cells. The cells were transfected with siPDIs for 48 h prior to treatment with SAS (0.5 mM), and the cellular levels of ROS after 8 h of SAS exposure were assessed by flow cytometry (the corresponding intensity values are shown on the right; n = 3). (M,N) Effects of PDI knockdown on total iNOS and monomeric and dimeric iNOS levels in SAS-treated H9C2 (M) and BRL-3A (N) cells. The cells were transfected with siCon or PDI siRNAs for 48 h and then treated with 0.5 mM SAS for an additional 6 h. The quantitative data are presented as the mean ± SD. * or #P < 0.05; ** or ## P < 0.01; n.s., not significant.

Next, we selectively downregulate PDI expression in H9C2 cells via PDI-siRNAs for 24 and 48 h. Western blot analysis confirmed the effectiveness of *PDI* knockdown at the protein level ( Figure 8A). Next, we assessed the cytotoxicity of SAS at two different concentrations (0.25 and 0.5 mM) in H9C2 cells. *PDI* knockdown significantly reduced the sensitivity of H9C2 cells to SAS-induced cytotoxicity ( Figure 8B for 24-h *PDI* knockdown; Figure 8C for 48-h *PDI* knockdown). Furthermore, the SAS-induced accumulation of NO ( Figure 8D), ROS ( Figure 8E) and lipid-ROS ( Figure 8F) was diminished by *PDI* knockdown for 48 h.

Similar observations on the role of PDI in mediating SAS-induced cell death were also made in BRL-3A cells. We found that *PDI* knockdown (verified by western blot analysis, Figure 8G) significantly reduced the sensitivity of BRL-3A cells to SAS-induced cytotoxicity ( Figure 8H for 24 h; Figure 8I for 48 h). Furthermore, the SAS-induced accumulation of NO ( Figure 8J), ROS ( Figure 8K) and lipid-ROS ( Figure 8L) was partially reduced by *PDI* knockdown.

To investigate whether PDI catalyzes the dimerization of iNOS in SAS-treated cells, we determined the levels of NOS dimer proteins in H9C2 and BRL-3A cells with or without *PDI* knockdown. We found that *PDI* knockdown abrogated SAS-induced iNOS dimer levels in these cells ( Figure 8M for H9C2 cells; Figure 8N for BRL-3A cells). Notably, *PDI* knockdown also abrogated the SAS-induced increase in total iNOS protein levels ( Figure 8M,N). To provide additional support for the notion that PDI is involved in mediating SAS-induced cell death in both H9C2 and BRL-3A cells, we also examined the protective effects of several known PDI inhibitors, including SNAP, cystamine, 4-OH-E _1_ and BAZ. The results are summarized below.

#### SNAP

**Figure 9:**
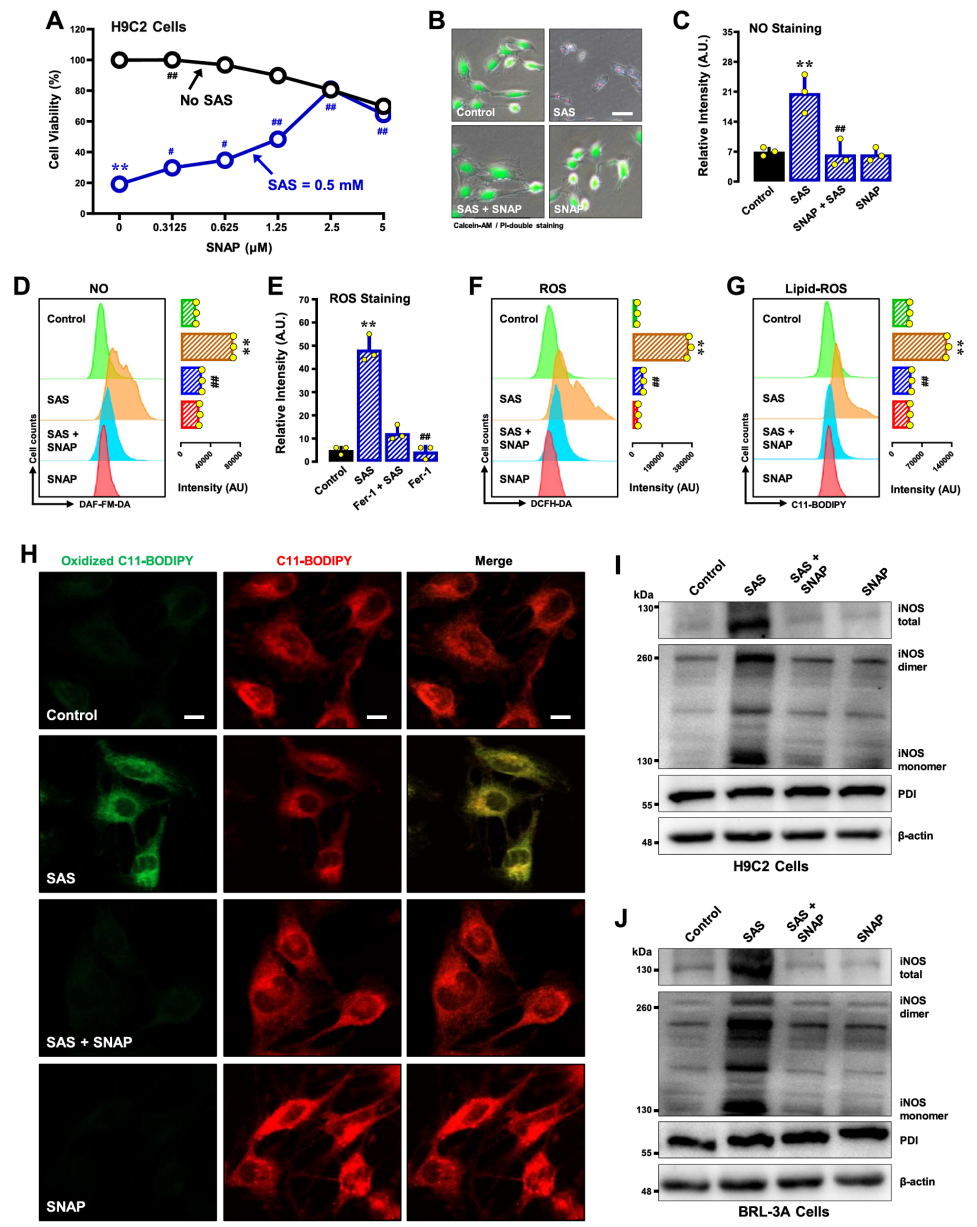
Figure 9 Effect of SNAP on SAS-induced ferroptosis and the accumulation of NO, ROS and lipid-ROS in H9C2 and BRL-3A cells (A,B) Protective effect of SNAP against SAS-induced cytotoxicity in H9C2 cells. In (A), cells were treated with SAS (0.5 mM) ± SNAP (0.15625, 0.3125, 0.625, 1.25, 2.5 and 5 μM) for 24 h, and cell viability was determined by MTT assay (n = 5). In (B), cells were treated with SAS (0.5 mM) ± SNAP (2.5 μM) for 24 h, and then, fluorescence images of calcein-AM/PI-stained cells were captured (green for live cells and red for dead cells; scale bar = 100 μm). (C-G) Abrogation by BAZ of SAS-induced accumulation of cellular NO (C,D), ROS (E,F) and lipid-ROS (G) in H9C2 cells. The cells were treated with SAS (0.5 mM) ± SNAP (2.5 μM) for 8 h and then subjected to flow cytometry (D,F,G) and fluorescence microscopy (C,E; scale bar = 100 μm). For the fluorescence microscopy data in (C,E), only the quantitative intensity values are shown (n = 3). For the flow cytometry data (D,F,G), the corresponding intensity values are shown on the right (n = 3). (H) Abrogation by SNAP of SAS-induced accumulation of cellular lipid-ROS (confocal microscopy, scale bar = 100 μm) in H9C2 cells. (I,J) Effect of SNAP on SAS-induced changes in total, monomeric and dimeric iNOS protein levels in H9C2 (I) and BRL-3A (J) cells. The cells were treated with SAS (0.5 mM) ± SNAP (2.5 μM) for 8 h, after which the levels of total, monomeric and dimeric iNOS proteins and PDI were determined by western blot analysis. β-Actin was used as a loading control. The quantitative data are presented as the mean±SDs. * or #P < 0.05; ** or ## P < 0.01; n.s., not significant.

SNAP is an *S*-nitrosylating agent that can inhibit the function of PDI by increasing its *S*-nitrosylation [49]. In this study, we found that SNAP (2.5 μM) elicited modest protection against SAS-induced ferroptosis in H9C2 cells ( Figure 9A,B). SNAP also abrogated SAS-induced accumulation of NO ( Figure 9C,D), ROS ( Figure 9E,F) and lipid-ROS ( Figure 9G,H). In BRL-3A cells, SNAP exhibited a similar protective effect against SAS-induced ferroptosis ( Supplementary Figure S7A,B) and eliminated the accumulation of NO ( Supplementary Figure S7C), ROS ( Supplementary Figure S7D) and lipid-ROS ( Supplementary Figure S7E,F). As expected, SNAP also abrogated the SAS-induced increase in iNOS dimer and total iNOS protein levels ( Figure 9I for H9C2 cells; Figure 9J for BRL-3A cells).

#### Cystamine

**Figure 10:**
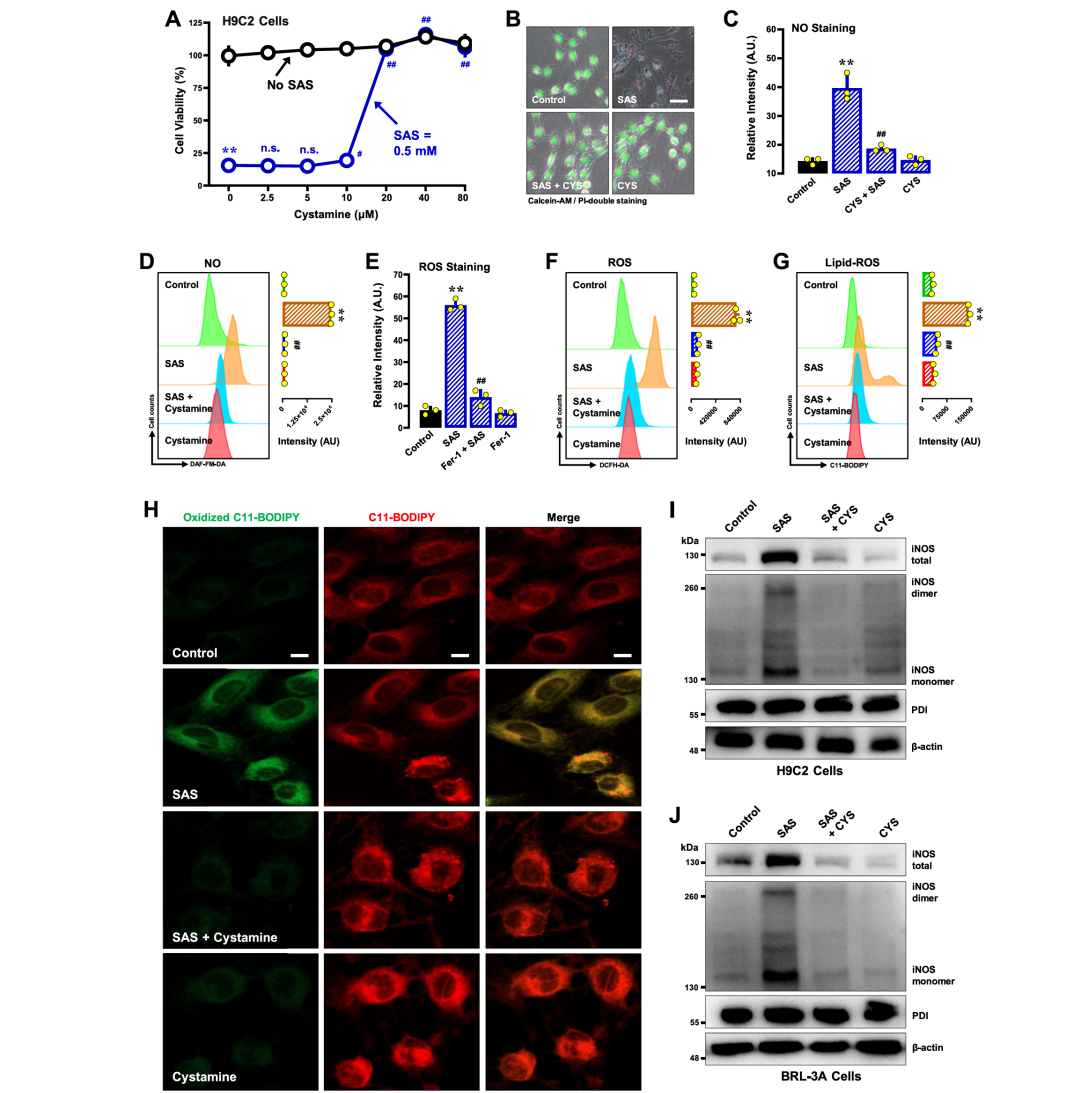
Figure 10 Effect of cystamine on SAS-induced ferroptosis and the accumulation of NO, ROS and lipid-ROS in H9C2 and BRL-3A cells (A,B) Protective effect of cystamine against SAS-induced cytotoxicity in H9C2 cells. In (A), the cells were treated with SAS (0.5 mM)±cystamine (2.5, 5, 10, 20, 40 or 80 μM) for 24 h and then subjected to MTT assay ( n = 5). In (B), cells were treated with SAS (0.5 mM) ± cystamine (40 μM) for 24 h, after which fluorescence images of calcein-AM/PI-stained cells were captured (green for live cells and red for dead cells; scale bar = 100 μm). (C–G) Abrogation by cystamine of SAS-induced accumulation of cellular NO (C,D), ROS (E,F) and lipid-ROS (G) in H9C2 cells. H9C2 cells were treated with SAS (0.5 mM) ± cystamine (40 μM) for 8 h and then subjected to flow cytometry (D,F,G) and fluorescence microscopy (C,E; scale bar = 100 μm). For the fluorescence microscopy data in (C and E), only the quantitative intensity values are shown (n = 3). For the flow cytometry data (D, F, G), the corresponding intensity values are shown on the right ( n = 3). (H) Abrogation by cystamine of SAS-induced accumulation of cellular lipid-ROS (confocal microscopy, scale bar = 100 μm) in H9C2 cells. (I,J) Effects of cystamine on SAS-induced changes in total, monomeric and dimeric iNOS protein levels in H9C2 (I) and BRL-3A (J) cells. The cells were treated with SAS (0.5 mM) ± cystamine (40 μM) for 8 h, after which the levels of total, monomeric and dimeric iNOS proteins and PDI were determined by western blot analysis. β-Actin was used as a loading control. The quantitative data are presented as the mean ± SD. ** or ##P<0.01; n.s., not significant.

Cystamine is a PDI inhibitor that can covalently modify the cysteine residue(s) in the catalytic region of PDI [50], thereby inhibiting its enzymatic activity [ 23, 25, 51]. We found that combined treatment of H9C2 cells with cystamine effectively prevented SAS-induced ferroptotic cell death when cystamine was present at ≥ 20 μM ( Figure 10A for the MTT assay; Figure 10B for calcein-AM/PI double staining). Cystamine also effectively abrogated SAS-induced accumulation of cellular NO ( Figure 10C,D), ROS ( Figure 10E,F) and lipid-ROS ( Figure 10G,H). Similarly, cystamine prevented SAS-induced ferroptotic cell death in BRL-3A cells ( Supplementary Figure S8A,B) and reduced the accumulation of NO ( Supplementary Figure S8C), ROS ( Supplementary Figure S8D) and lipid-ROS ( Supplementary Figure S8E,F). In addition, cystamine abrogated the SAS-induced increase in total iNOS protein levels and their respective dimers ( Figure 10I for H9C2 cells; Figure 10J for BRL-3A cells).

#### BAZ

**Figure 11:**
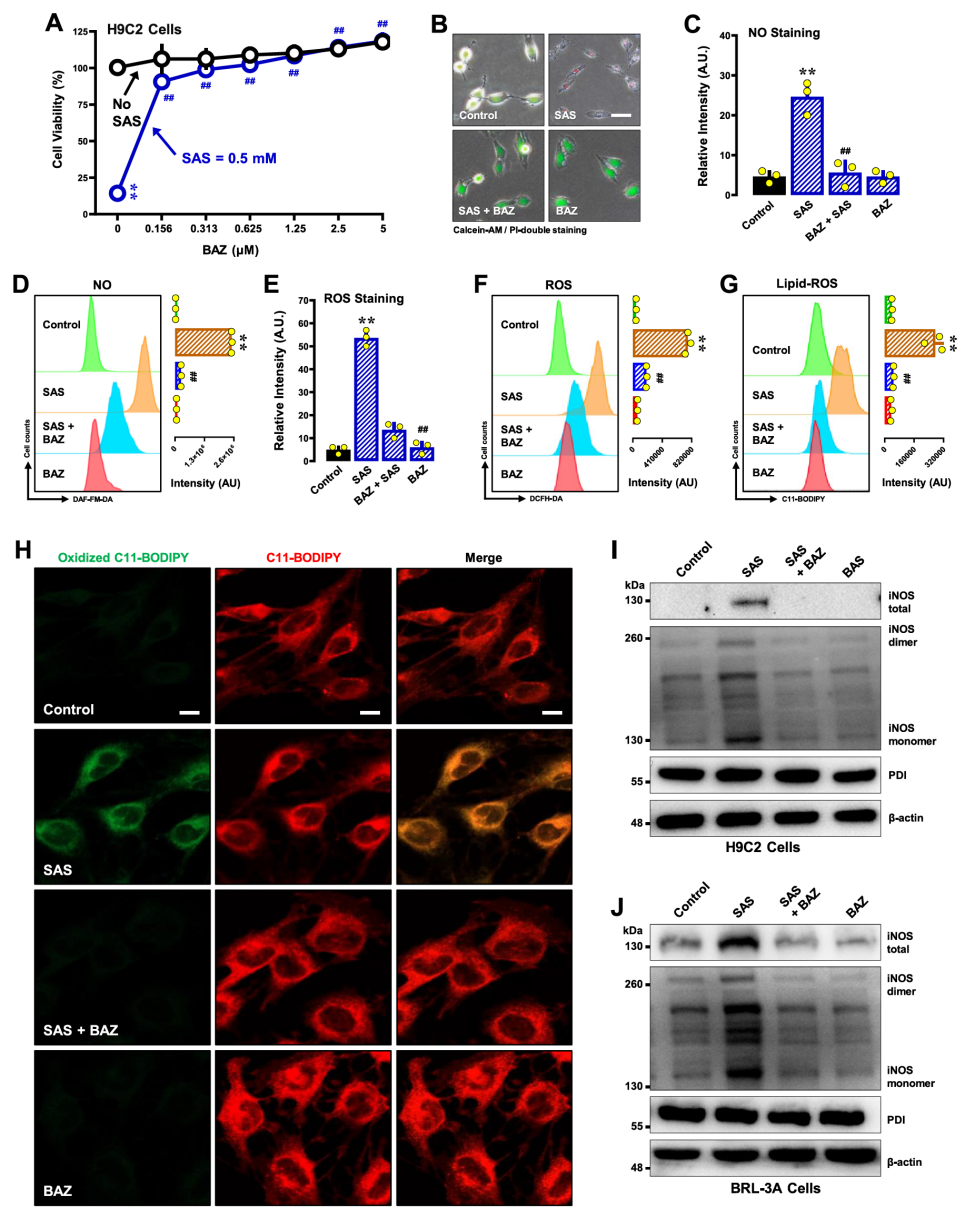
Figure 11 Effect of BAZ on SAS-induced ferroptosis and the accumulation of NO, ROS and lipid-ROS in H9C2 and BRL-3A cells (A,B) Protective effect of BAZ against SAS-induced cytotoxicity in H9C2 cells. In A, cells were treated with SAS (0.5 mM) ± BAZ (0.15625, 0.3125, 0.625, 1.25, 2.5 and 5 μM) for 24 h, and cell viability was determined by MTT assay (n = 5). In B, cells were treated with SAS (0.5 mM) ± BAZ (2.5 μM) for 24 h, and then, fluorescence images of calcein-AM/PI-stained cells were captured (green for live cells and red for dead cells; scale bar = 100 μm). (C–G) Abrogation by BAZ of SAS-induced accumulation of cellular NO (C,D), ROS (E,F) and lipid-ROS (G) in H9C2 cells. The cells were treated with SAS (0.5 mM) ± BAZ (2.5 μM) for 8 h and then subjected to flow cytometry (D,F,G) and fluorescence microscopy (C,E; scale bar = 100 μm). For the fluorescence microscopy data in (C,E), only the quantitative intensity values are shown (n = 3). For the flow cytometry data (D,F,G), the corresponding intensity values are shown on the right ( n = 3). (H) Abrogation by BAZ of SAS-induced accumulation of cellular lipid-ROS (confocal microscopy, scale bar=100 μm) in H9C2 cells. (I,J) Effect of BAZ on SAS-induced changes in total, monomeric and dimeric iNOS protein levels in H9C2 (I) and BRL-3A (J) cells. The cells were treated with SAS (0.5 mM) ± BAZ (2.5 μM) for 8 h, after which the levels of total, monomeric and dimeric iNOS proteins and PDI were determined by western blot analysis. β-Actin was used as a loading control. The quantitative data are presented as the mean± SD. ** or ## P < 0.01; n.s., not significant.

BAZ, a synthetic selective estrogen receptor modulator [52], was recently found to be a noncovalent inhibitor of PDI with very high potency and can strongly protect cells against chemically induced ferroptosis [53]. We found that BAZ strongly protects against SAS-induced cytotoxicity in H9C2 cells at very low concentrations (at ≥ 156 nM) ( Figure 11A,B). BAZ effectively abrogated the SAS-induced accumulation of cellular NO ( Figure 11C,D), ROS ( Figure 11E,F) and lipid-ROS ( Figure 11G,H). Similar observations were made in BRL-3A cells. BAZ protected against SAS-induced ferroptosis at low concentrations ( Supplementary Figure S9A,B), along with abrogation of SAS-induced accumulation of cellular NO ( Supplementary Figure S9C), ROS ( Supplementary Figure S9D) and lipid-ROS ( Supplementary Figure S9E,F). Consistent with its strong ability to inhibit PDI, BAZ strongly abrogated the SAS-induced increase in iNOS dimer levels, along with a reduction in total iNOS protein levels ( Figure 11I for H9C2 cells; Figure 11J for BRL-3A cells).

#### 4-OH-E _1_

**Figure 12:**
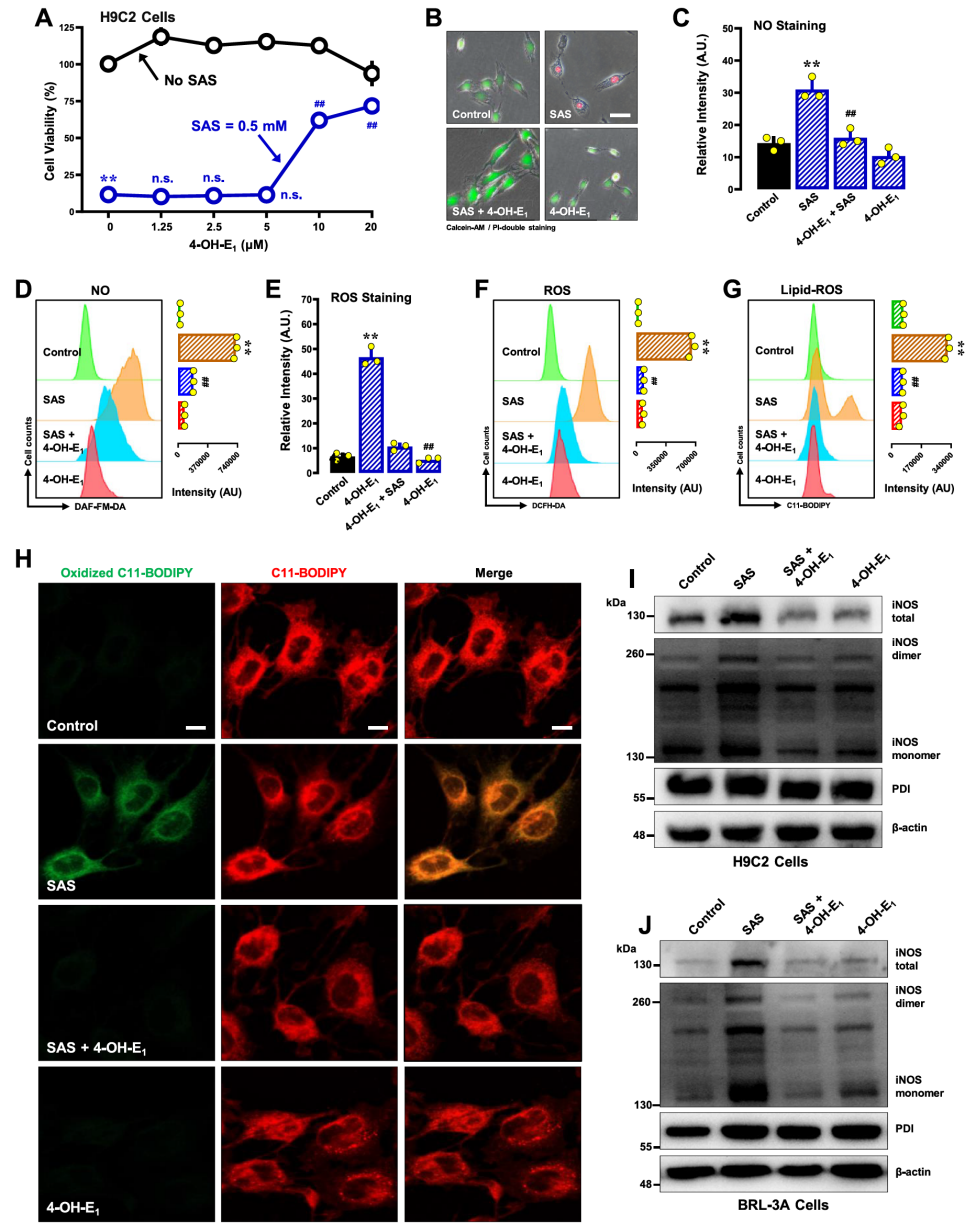
Figure 12 Effect of 4-OH-E _1_ on SAS-induced ferroptosis and the accumulation of NO, ROS and lipid-ROS in H9C2 and BRL-3A cells (A,B) The protective effect of 4-OH-E1 against SAS-induced toxicity was investigated in H9C2 cells. In A, cells were exposed to SAS (0.5 mM) ± 4-OH-E 1 (1.25, 2.5, 5, 10, and 20 μM) for a duration of 24 h, and the subsequent assessment of cell viability was conducted through MTT assay (n = 5). In B, cells were treated with SAS (0.5 mM) ± 4-OH-E1 (20 μM) for 24 h, and then fluorescence images of calcein-AM/PI-stained cells were captured (green for live cells and red for dead cells; scale bar = 100 μm). (C–G) Abrogation by 4-OH-E1 of SAS-induced accumulation of cellular NO (C,D), ROS (E,F) and lipid-ROS (G) in H9C2 cells. The cells were treated with SAS (0.5 mM) ± 4-OH-E1 (20 μM) for 8 h and then subjected to flow cytometry (D,F,G) and fluorescence microscopy (C,E; scale bar = 100 μm). For the fluorescence microscopy data in (C and E), only the quantitative intensity values are shown (n = 3). For the flow cytometry data (D,F,G), the corresponding intensity values are shown on the right (n = 3). (H) Abrogation by 4-OH-E 1 of SAS-induced accumulation of cellular lipid-ROS (confocal microscopy, scale bar = 100 μm) in H9C2 cells. (I,J) Effects of 4-OH-E1 on SAS-induced changes in total, monomeric and dimeric iNOS protein levels in H9C2 (I) and BRL-3A (J) cells. The cells were treated with SAS (0.5 mM) ± 4-OH-E1 (20 μM) for 8 h, after which the levels of total, monomeric and dimeric iNOS proteins and PDI were determined by western blot analysis. β-Actin was used as a loading control. The quantitative data are presented as the mean±SD. ** or ## P < 0.01; n.s., not significant.

4-OH-E _1_ is an endogenous estrone metabolite that can noncovalently inhibit PDI’s catalytic function [54]. Recently, it was shown that 4-OH-E _1_ can provide strong protection against chemically induced ferroptosis [48]. In this study, 4-OH-E _1_ was found to have modest protection against SAS-induced cytotoxicity in H9C2 cells when it was present at 10–20 μM ( Figure 12A,B). In a parallel manner, 4-OH-E _1_ also reduced the SAS-induced accumulation of NO ( Figure 12C,D), ROS ( Figure 12E,F) and lipid-ROS ( Figure 12G,H). Similar observations were made in BRL-3A cells. We found that 4-OH-E _1_ partially prevented SAS-induced cytotoxicity in BRL-3A cells ( Supplementary Figure S10A,B). 4-OH-E _1_ also reduced the accumulation of NO ( Supplementary Figure S10C), ROS ( Supplementary Figure S10D) and lipid-ROS ( Supplementary Figure S10E,F). In addition, joint treatment of cells with 4-OH-E _1_ also abrogated SAS-induced increases in total iNOS protein levels and their respective dimers ( Figure 12I for H9C2 cells; Figure 12J for BRL-3A cells).

Collectively, the above observations with PDI inhibitors underscore the pivotal role of PDI in mediating SAS-induced activation of NOS, the subsequent buildup of cellular NO and ROS/lipid-ROS, and ultimately ferroptotic cell death.

## Discussion

In the past several years, several studies have investigated the ferroptosis-inducing ability of SAS in different cell lines, mostly cancer cells, in culture [ 10– 12]. Recently, we reported the involvement of PDI in SAS-induced ferroptosis in immortalized HT22 mouse hippocampal neurons [15]. The present study sought to characterize the detailed cellular and biochemical mechanisms of SAS-induced ferroptotic cell death in two additional cell lines, namely, H9C2 cardiomyocytes and BRL-3A hepatocytes. The experimental evidence presented in this study jointly supports the notion that the PDI–NOS–NO–ROS/lipid–ROS pathway plays a pivotal role in mediating SAS-induced oxidative cytotoxicity.

First, this study confirmed that SAS-induced cell death closely resembles oxidative ferroptosis on the basis of the observations that SAS-induced cell death can be effectively rescued by Fer-1 and DFO but not by Nec-1 and z-VAD-FMK. Next, a series of experimental evidence is presented in this study for the hypothesis that NO accumulation in SAS-treated cells is an early event which then results in the accumulation of cellular ROS and lipid-ROS, and ultimately ferroptotic cell death. (i) A modest increase in NO levels is observed in H9C2 and BRL-3A cells at 1–2 h following SAS exposure, and a significant buildup of cellular ROS and lipid-ROS is evident at ~4 h. This observation suggests that in SAS-treated cells, cellular NO buildup takes place first, followed by the accumulation of cellular ROS and lipid-ROS. (ii) SNP, which can directly release NO intracellularly [43], strongly increases the sensitivity of H9C2 and BRL-3A cells to SAS-induced cytotoxicity. This heightened sensitivity is attributed to an accelerated buildup of cellular ROS and lipid-ROS, resulting in a swift escalation in cellular NO levels. (iii) cPTIO, an NO scavenger [37], not only mitigates SAS-induced NO accumulation but also decreases the accumulation of ROS and lipid-ROS triggered by SAS. The protective effect of cPTIO is associated with its ability to counteract SAS-induced cell death. Collectively, these observations provide substantial evidence for the hypothesis that NO is a more upstream mediator than ROS and lipid-ROS in SAS-induced ferroptosis in cultured cells.

The accumulation of cellular ROS, particularly lipid-ROS, is pivotal for chemically induced oxidative ferroptosis [ 16, 18, 55, 56]. In this study, we demonstrated that the accumulation of ROS and lipid-ROS in SAS-treated H9C2 and BRL-3A cells also plays an important role in ferroptosis progression. For example, NAC, a well-known antioxidant [ 39 , 40], can effectively eliminate the accumulation of cellular NO, ROS and lipid-ROS in SAS-treated cells, thereby providing robust cytoprotection. Similarly, Fer-1, an aromatic primary amine with the capacity to suppress lipid peroxidation [28], can also effectively rescue SAS-induced ferroptosis in H9C2 and BRL-3A cells. Notably, while Fer-1 potently suppresses SAS-induced lipid-ROS accumulation, its effect on SAS-induced NO and ROS accumulation is relatively modest. This is not unexpected, given that Fer-1 is predominantly a scavenger of cellular lipid-ROS, which are downstream of the cellular NO and ROS in the proposed iNOS→NO→ROS→lipid-ROS cascade. Overall, this study revealed that SAS-induced cellular NO accumulation occurs prior to cellular ROS and lipid-ROS accumulation and that cellular NO, ROS and lipid-ROS jointly drive oxidative ferroptosis in SAS-treated H9C2 and BRL-3A cells.

NOS catalyzes the production of NO by oxidizing *l*-arginine to *l*-citrulline [ 57, 58]. Notably, iNOS exists in two structural forms: dimeric iNOS, which is catalytically active and essential for NO synthesis, and monomeric iNOS, which is catalytically inactive. In this study, we found that SAS treatment increased iNOS dimerization in a concentration- and time-dependent manner in H9C2 and BRL-3A cells. In addition, we showed that treatment of these cells with SAS resulted in a marked increase in total cellular iNOS protein levels. The upregulation of iNOS, particularly increased iNOS dimerization, can result in increased NO production, which, under certain conditions, can be highly cytotoxic [ 59, 60]. Interestingly, despite these changes in iNOS levels and activity, the cellular levels of PDI, which is the enzyme that catalyzes iNOS dimerization, remain largely unaffected.

To provide experimental support for the suggestion that iNOS dimerization leads to NO and ROS/lipid-ROS accumulation and ultimately cell death, we treated H9C2 and BRL-3A cells with SMT, an iNOS inhibitor, and found that SMT attenuates SAS-induced iNOS upregulation and dimerization as well as NO accumulation, which is accompanied by reductions in SAS-induced ROS/lipid-ROS accumulation and ferroptosis. Similarly, iNOS knockdown also partially eliminates SAS cytotoxicity. Taken together, these results demonstrate that SAS-induced iNOS upregulation and dimerization critically contribute to SAS-induced ferroptosis.

In this study, we also provide evidence that PDI is a pivotal upstream mediator of SAS-induced ferroptosis by catalyzing iNOS dimerization, which then leads to NO and ROS/lipid-ROS accumulation and, ultimately, oxidative cell death. Specifically, we found that the knockdown of *PDI* with specific PDI siRNAs strongly attenuates SAS-induced NO and ROS/lipid-ROS accumulation, and protects against SAS-induced cell death.

Like *PDI* knockdown, several PDI inhibitors also have protective effects against SAS-induced ferroptosis in H9C2 and BRL-3A cells. SNAP, a thiol-nitrosylating agent [49], can enhance PDI *S*-nitrosylation [49]. In our previous work, we showed that PDI predominantly exists in the *S*-nitrosylated state in untreated HT22 cells and that exposure to glutamate induces PDI *S*-denitrosylation [49]. This shift promotes PDI oxidation and activation, enabling it to catalyze NOS dimerization. Earlier, we reported that SNAP, which has some cytotoxicity when present alone [ 21, 23, 24], partially rescued glutamate-induced oxidative cytotoxicity (oxytosis) through increased PDI *S*-nitrosylation [21]. In addition, a similar rescue effect of SNAP was observed during erastin-induced ferroptosis [ 23, 24]. In the present study, we found that SNAP also provides significant protection against SAS-induced ferroptosis, which is associated with the suppression of SAS-induced iNOS upregulation and dimerization. As expected, these effects are associated with a marked decrease in cellular NO, ROS and lipid-ROS levels.

Like SNAP, we found that cystamine, a covalent inhibitor of PDI [ 25, 61, 62], can strongly attenuate SAS-induced iNOS dimerization and cellular NO and ROS/lipid-ROS accumulation, which is accompanied by ferroptosis protection. Additionally, cystamine inhibits SAS-induced iNOS upregulation, which further contributes to its cytoprotective properties. Importantly, cystamine is not a reducing agent but a mild oxidizing agent, and it can covalently modify the free thiol groups in PDI catalytic sites, resulting in inhibition of PDI catalytic function. The strong protective effect of cystamine underscores the pivotal upstream role of PDI in SAS-induced ferroptosis.

Unlike cystamine, BAZ and 4-OH-E _1_ can inhibit PDI’s catalytic activities through noncovalent binding interactions with PDI [ 48, 53]. BAZ strongly protects against SAS-induced cell death. This cytoprotective effect is closely associated with the inhibition of PDI-mediated iNOS dimerization in SA-treated cells, along with the abrogation of cellular NO, ROS and lipid-ROS accumulation. Like BAZ, 4-OH-E _1_ also suppresses PDI-mediated iNOS dimerization in SAS-treated cells, which is associated with reduced accumulation of cellular NO, ROS and lipid-ROS. Notably, compared with BAZ, 4-OH-E _1_ has relatively weaker efficacy in abrogating SAS-induced ROS buildup and cell death because 4-OH-E _1_ itself is a catechol (which is chemically reactive) and can undergo oxidation/auto-oxidation to generate ROS under certain conditions [62]. Together, the experimental evidence discussed here with several PDI inhibitors offers additional support for the pivotal role of PDI in mediating SAS-induced ferroptosis. Additionally, these results highlight the importance of PDI as a crucial drug target for protection against oxidative ferroptosis.

Finally, regarding the potential causes of PDI activation ( *i*. *e*., oxidation) in SAS-treated cells, first, it is ruled out in this study that PDI can be directly activated by SAS (Supplementary Figure S6). As depicted in Supplementary Figure S11, PDI is favored to be in the oxidized state when cellular GSH levels are low, such as in the presence of SAS or erastin. These chemicals can slowly deplete free GSH, and because of this slow process, PDI activation and subsequent NOS dimerization will also take considerable time to occur. However, in the case of RSL3, which is an inhibitor of TrxR1 [ 24, 63], PDI activation and NOS dimerization can occur more quickly, and as a result, RSL3-induced ferroptosis also occurs much faster [24].

As summarized in Supplementary Figure S11, the present study shows that SAS is capable of inducing ferroptosis in H9C2 cardiomyocytes and BRL-3A hepatocytes in culture, and this process is associated with time dependent, sequential increases in cellular NO, ROS and lipid-ROS levels. Treatment of these cells with SAS activates PDI-mediated iNOS dimerization, which then activates the catalytic activity of iNOS for NO production. Furthermore, SAS also increases iNOS protein levels in these cells, which also contributes to elevated NO production and accumulation. Genetic manipulation of PDI expression and pharmacological inhibition of PDI catalytic activity can effectively abrogate SAS-induced iNOS dimerization and cellular NO, ROS and lipid-ROS accumulation, along with strong protection against ferroptotic cell death. Collectively, the findings of this study, along with our recent observations [15], jointly demonstrate a pivotal role of PDI in SAS-induced ferroptosis through the activation of the PDI→NOS→NO→ROS/lipid-ROS pathway and offer new strategies for sensitizing cancer cells to SAS-induced ferroptosis, such as through the use of NO-releasing agents or TrxR1 inhibitors.

## Supporting information

Supplementary_Table_S1

JYC3_Figures_Final_2025-4-10

Supplementary_Figures_Final_2025-4-10

## Supplementary Data

Supplementary data is available at *Acta Biochimica et Biophysica Sinica* online.

## References

[1] Zenlea T (2014). Immunosuppressive therapies for inflammatory bowel disease. World J Gastroenterol.

[2] Martin F (1987). Oral 5-aminosalicylic acid preparations in treatment of inflammatory bowel disease an update. Digest Dis Sci.

[3] Peppercorn MA (1984). Sulfasalazine. Pharmacology, clinical use, toxicity, and related new drug development. Ann Intern Med.

[4] Yan X, Li Q, Xiao S, Chen J, Song W (2025). Sulfasalazine-loaded nanoframes: a new frontier in bladder cancer therapy through ferroptosis induction. Colloids Surfs B-Biointerfaces.

[5] Ma M, Chen G, Wang P, Lu W, Zhu C, Song M, Yang J (2015). Xc-inhibitor sulfasalazine sensitizes colorectal cancer to cisplatin by a GSH-dependent mechanism. Cancer Lett.

[6] Sugiyama A, Ohta T, Obata M, Takahashi K, Seino M, Nagase S (2020). xCT inhibitor sulfasalazine depletes paclitaxel‑resistant tumor cells through ferroptosis in uterine serous carcinoma. Oncol Lett.

[7] Lee J, Roh JL (2022). SLC7A11 as a gateway of metabolic perturbation and ferroptosis vulnerability in cancer. Antioxidants.

[8] Wang K, Zhang X, Fan Y, Zhou L, Duan Y, Li S, Sun Z (2024). Reactivation of MAPK-SOX2 pathway confers ferroptosis sensitivity in KRASG12C inhibitor resistant tumors. Redox Biol.

[9] Subramanian C, McNamara K, Croslow SW, Tan Y, Hess D, Kiseljak-Vassiliades K, Wierman ME (2025). Novel repurposing of sulfasalazine for the treatment of adrenocortical carcinomas, probably through the SLC7A11/xCT-hsa-miR-92a-3p-OIP5-AS1 network pathway. Surgery.

[10] Usukhbayar N, Uesugi S, Kimura K (2023). 3,6-Epidioxy-1,10-bisaboladiene and sulfasalazine synergistically induce ferroptosis-like cell death in human breast cancer cell lines. Biosci Biotechnol Biochem.

[11] Yamaguchi Y, Kasukabe T, Kumakura S (2018). Piperlongumine rapidly induces the death of human pancreatic cancer cells mainly through the induction of ferroptosis. Int J Oncol.

[12] Sontheimer H, Bridges RJ (2012). Sulfasalazine for brain cancer fits. Expert Opin Investal Drugs.

[13] Sun S, Guo C, Gao T, Ma D, Su X, Pang Q, Zhang R (2022). Hypoxia enhances glioma resistance to sulfasalazine-induced ferroptosis by upregulating SLC7A11 via PI3K/AKT/HIF-1
*α* axis. Oxid Med Cell Longev.

[14] Chen X, Comish PB, Tang D, Kang R (2021). Characteristics and biomarkers of ferroptosis. Front Cell Dev Biol.

[15] Wu Y, Zhu BT (2025). Role of protein disulfide isomerase in mediating sulfasalazine-induced ferroptosis in HT22 cells: the PDI-NOS-NO-ROS/lipid-ROS cascade. Arch Biochem Biophys.

[16] Dixon SJ, Lemberg KM, Lamprecht MR, Skouta R, Zaitsev EM, Gleason CE, Patel DN (2012). Ferroptosis: an iron-dependent form of nonapoptotic cell death. Cell.

[17] Rochette L, Dogon G, Rigal E, Zeller M, Cottin Y, Vergely C (2023). Lipid peroxidation and iron metabolism: two corner stones in the homeostasis control of ferroptosis. Int J Mol Sci.

[18] Latunde-Dada GO (2017). Ferroptosis: Role of lipid peroxidation, iron and ferritinophagy. Biochim Biophys Acta Gen Subj.

[19] Freedman RB, Hirst TR, Tuite MF (1994). Protein disulphide isomerase: building bridges in protein folding. Trends Biochem Sci.

[20] Noiva R (1999). Protein disulfide isomerase: the multifunctional redox chaperone of the endoplasmic reticulum. Semin Cell Dev Biol.

[21] Okada K, Fukui M, Zhu BT (2016). Protein disulfide isomerase mediates glutathione depletion-induced cytotoxicity. Biochem Biophys Res Commun.

[22] Wang H, Wang P, Zhu BT (2022). Mechanism of erastin-induced ferroptosis in MDA-MB-231 human breast cancer cells: evidence for a critical role of protein disulfide isomerase. Mol Cell Biol.

[23] Hou MJ, Wang P, Zhu BT (2023). Biochemical mechanism of erastin-induced ferroptotic cell death in neuronal cells. Acta Biochim Biophys Sin.

[24] Hou MJ, Huang X, Zhu BT (2025). Mechanism of RSL3-induced ferroptotic cell death in HT22 cells: crucial role of protein disulfide isomerase. Acta Biochim Biophys Sin.

[25] Zhu YY, Zhang Q, Jia YC, Hou MJ, Zhu BT (2024). Protein disulfide isomerase plays a crucial role in mediating chemically-induced, glutathione depletion-associated hepatocyte injury
*in vitro* and
*in vivo*. Cell Commun Signal.

[26] Song X, Hao X, Zhu BT (2024). Role of mitochondrial reactive oxygen species in chemically-induced ferroptosis. Free Radical Biol Med.

[27] Huang X, Hou MJ, Zhu BT (2024). Protection of HT22 neuronal cells against chemically-induced ferroptosis by catechol estrogens: protein disulfide isomerase as a mechanistic target. Sci Rep.

[28] Chu J, Liu CX, Song R, Li QL (2020). Ferrostatin-1 protects HT-22 cells from oxidative toxicity. Neural Regen Res.

[29] Lin S, Gao W, Zhu C, Lou Q, Ye C, Ren Y, Mehmood R (2022). Efficiently suppress of ferroptosis using deferoxamine nanoparticles as a new method for retinal ganglion cell protection after traumatic optic neuropathy. BioMater Adv.

[30] Dayani PN, Bishop MC, Black K, Zeltzer PM (2004). Desferoxamine (DFO)-mediated iron chelation: rationale for a novel approach to therapy for brain cancer. J Neurooncol.

[31] Yan X, Yan Y, Liu J, Jing Y, Hao P, Chen X, Li X (2024). Necrostatin-1 protects corneal epithelial cells by inhibiting the RIPK1/RIPK3/MLKL cascade in a benzalkonium chloride-induced model of necroptosis. Exp Eye Res.

[32] Cao L, Mu W (2021). Necrostatin-1 and necroptosis inhibition: pathophysiology and therapeutic implications. Pharmacol Res.

[33] Van Noorden CJF (2001). The history of Z-VAD-FMK, a tool for understanding the significance of caspase inhibition. Acta Histochemica.

[34] Rajah T, Chow SC (2014). The inhibition of human T cell proliferation by the caspase inhibitor z-VAD-FMK is mediated through oxidative stress. Toxicol Appl Pharmacol.

[35] Friedmann Angeli JP, Schneider M, Proneth B, Tyurina YY, Tyurin VA, Hammond VJ, Herbach N (2014). Inactivation of the ferroptosis regulator Gpx4 triggers acute renal failure in mice. Nat Cell Biol.

[36] Firouzjaei AA, Mohammadi-Yeganeh S (2024). The intricate interplay between ferroptosis and efferocytosis in cancer: unraveling novel insights and therapeutic opportunities. Front Oncol.

[37] Goldstein S, Russo A, Samuni A (2003). Reactions of PTIO and carboxy-PTIO with ·NO, ·NO2, and

$\;O_{2}^{\bar{.}}$. J Biol Chem.

[38] Akaike T, Yoshida M, Miyamoto Y, Sato K, Kohno M, Sasamoto K, Miyazaki K (1993). Antagonistic action of imidazolineoxyl N-oxides against endothelium-derived relaxing factor/.bul.NO (nitric oxide) through a radical reaction. Biochemistry.

[39] Wu YJ, Muldoon LL, Neuwelt EA (2005). The chemoprotective agent n-acetylcysteine blocks cisplatin-induced apoptosis through caspase signaling pathway. J Pharmacol Exp Ther.

[40] Tardiolo G, Bramanti P, Mazzon E (2018). Overview on the effects of N-acetylcysteine in neurodegenerative diseases. Molecules.

[41] Vorobjeva NV, Pinegin BV (2016). Effects of the antioxidants Trolox, Tiron and Tempol on neutrophil extracellular trap formation. Immunobiology.

[42] Chang B, Su Y, Li T, Zheng Y, Yang R, Lu H, et al. Mito-TEMPO ameliorates sodium palmitate induced ferroptosis in MIN6 cells through PINK1/Parkin-mediated mitophagy.
Biomed Environ Sci 2024, 37: 1128–1141. https://doi.org/10.3967/bes2024.111.

[43] Cobb A, Thornton L (2018). Sodium nitroprusside as a hyperinflation drug and therapeutic alternatives. J Pharmacy Pract.

[44] Förstermann U, Sessa WC (2012). Nitric oxide synthases: regulation and function. Eur Heart J.

[45] Balaganur V, Pathak NN, Lingaraju MC, More AS, Latief N, Kumari RR, Kumar D (2014). Effect of S-methylisothiourea, an inducible nitric oxide synthase inhibitor, in joint pain and pathology in surgically induced model of osteoarthritis. Connective Tissue Res.

[46] Pandey A, Singh G, Pandey S, Singh VK, Prasad SM (2025). 24-Epibrassinolide effectively alleviates UV-B stress-induced damage in the cyanobacterium Anabaena sp. PCC 7120 by employing nitric oxide: improved PS II photochemistry, antioxidant system, and growth. Plant Physiol Biochem.

[47] Zheng ZL, Wang XP, Hu YF, Li WG, Zhou Q, Xu F (2024). Propofol suppresses ferroptosis via modulating eNOS/NO signaling pathway to improve traumatic brain injury. Brain Behav.

[48] Wang H, Hou MJ, Liao L, Li P, Chen T, Wang P, Zhu BT (2024). Strong protection by 4-hydroxyestrone against erastin-induced ferroptotic cell death in estrogen receptor-negative human breast cancer cells: evidence for protein disulfide isomerase as a mechanistic target for protection. Biochemistry.

[49] Mallis RJ, Thomas JA (2000). Effect of S-nitrosothiols on cellular glutathione and reactive protein sulfhydryls. Arch Biochem Biophys.

[50] Hoffstrom BG, Kaplan A, Letso R, Schmid RS, Turmel GJ, Lo DC, Stockwell BR (2010). Inhibitors of protein disulfide isomerase suppress apoptosis induced by misfolded proteins. Nat Chem Biol.

[51] Chakravarthi S, Jessop CE, Bulleid NJ (2006). The role of glutathione in disulphide bond formation and endoplasmic-reticulum-generated oxidative stress. EMBO Rep.

[52] Jover-Mengual T, Castelló-Ruiz M, Burguete MC, Jorques M, López-Morales MA, Aliena-Valero A, Jurado-Rodríguez A (2017). Molecular mechanisms mediating the neuroprotective role of the selective estrogen receptor modulator, bazedoxifene, in acute ischemic stroke: a comparative study with 17β-estradiol. J Steroid Biochem Mol Biol.

[53] Hao X, Wang Y, Hou MJ, Yang YX, Liao L, Chen T, Wang P (2025). Strong protection by bazedoxifene against chemically-induced ferroptotic neuronal death
*in vitro* and
*in vivo*. Cell Commun Signal.

[54] Choi HJ, Lee AJ, Kang KS, Song JH, Zhu BT (2020). 4-Hydroxyestrone, an endogenous estrogen metabolite, can strongly protect neuronal cells against oxidative damage. Sci Rep.

[55] Tang D, Chen X, Kang R, Kroemer G (2021). Ferroptosis: molecular mechanisms and health implications. Cell Res.

[56] Li J, Cao F, Yin H, Huang Z, Lin Z, Mao N, Sun B (2020). Ferroptosis: past, present and future. Cell Death Dis.

[57] Stuehr DJ (2004). Enzymes of the l-arginine to nitric oxide pathway. J Nutr.

[58] Marletta MA, Hurshman AR, Rusche KM (1998). Catalysis by nitric oxide synthase. Curr Opin Chem Biol.

[59] MacMicking J, Xie Q, Nathan C (1997). Nitric oxide and macrophage function. Annu Rev Immunol.

[60] Kolodziejski PJ, Koo JS, Eissa NT (2004). Regulation of inducible nitric oxide synthase by rapid cellular turnover and cotranslational down-regulation by dimerization inhibitors. Proc Natl Acad Sci USA.

[61] Fujita I, Nobunaga M, Seki T, Kurauchi Y, Hisatsune A, Katsuki H (2017). Cystamine-mediated inhibition of protein disulfide isomerase triggers aggregation of misfolded orexin-A in the Golgi apparatus and prevents extracellular secretion of orexin-A. Biochem Biophys Res Commun.

[62] Zhu B (1998). Functional role of estrogen metabolism in target cells: review and perspectives. Carcinogenesis.

[63] Cheff DM, Huang C, Scholzen KC, Gencheva R, Ronzetti MH, Cheng Q, Hall MD (2023). The ferroptosis inducing compounds RSL3 and ML162 are not direct inhibitors of GPX4 but of TXNRD1. Redox Biol.

