# Supplementary material for: Characterization of the mechanisms underlying sulfasalazine-induced ferroptotic cell death: role of protein disulfide isomerase-mediated NOS activation and NO accumulation: Mechanism of SAS-induced cell death
Source: Acta Biochim Biophys Sin (Shanghai). 2025 Aug 21;57(12):2074–93. doi: 10.3724/abbs.2025100 (PMC12747976; doi:10.3724/abbs.2025100)
Supplement: Supplementary_Table_S1 [file Supplementary_Table_S1.pdf]

**Supplementary Table S1. The sequences of the siRNAs used in this study.**

| **Name** | **Sequence** |
|---|---|
| **Control siRNAs (siCon)** | 5′-AUCCGCGCGAUAGUACGUAtt-3′ **(#1)** |
| | 5′-UUACGCGUAGCGUAAUACGtt **(#2)** |
| | 5′-UAUUCGCGCGUAUAGCGGUtt-3′ **(#3)** |
| **PDI siRNAs (siPDI)** | 5′-CCAAGTACCAGCTGGACAA-3′ (#1) |
| | 5′-GAACGGTCATTGATTACAA-3′ (#3) |
| | 5′-TGCTAAGATGGACTCAACA-3′ (#2) |
| **iNOS siRNAs (siiNOS)** | 5′-UUCAAGUAGAUUAGCCAAGtt-3′ (#1) |
| | 5′-UACGUUAUGAACAGCUUCCtt (#2) |
| | 5′-UUACGUUAUGAACAGCUUCtt-3′ (#3) |
| | 5′-UUCAUGAUAACGUUUCUGGtt (#2) |
| | UAGUAGUCCACAAUAGUACtt-3′ (#3) |
