# Supplementary figures and images for: Characterization of the mechanisms underlying sulfasalazine-induced ferroptotic cell death: role of protein disulfide isomerase-mediated NOS activation and NO accumulation: Mechanism of SAS-induced cell death
Source: Acta Biochim Biophys Sin (Shanghai). 2025 Aug 21;57(12):2074–93. doi: 10.3724/abbs.2025100 (PMC12747976; doi:10.3724/abbs.2025100)

Figure 1

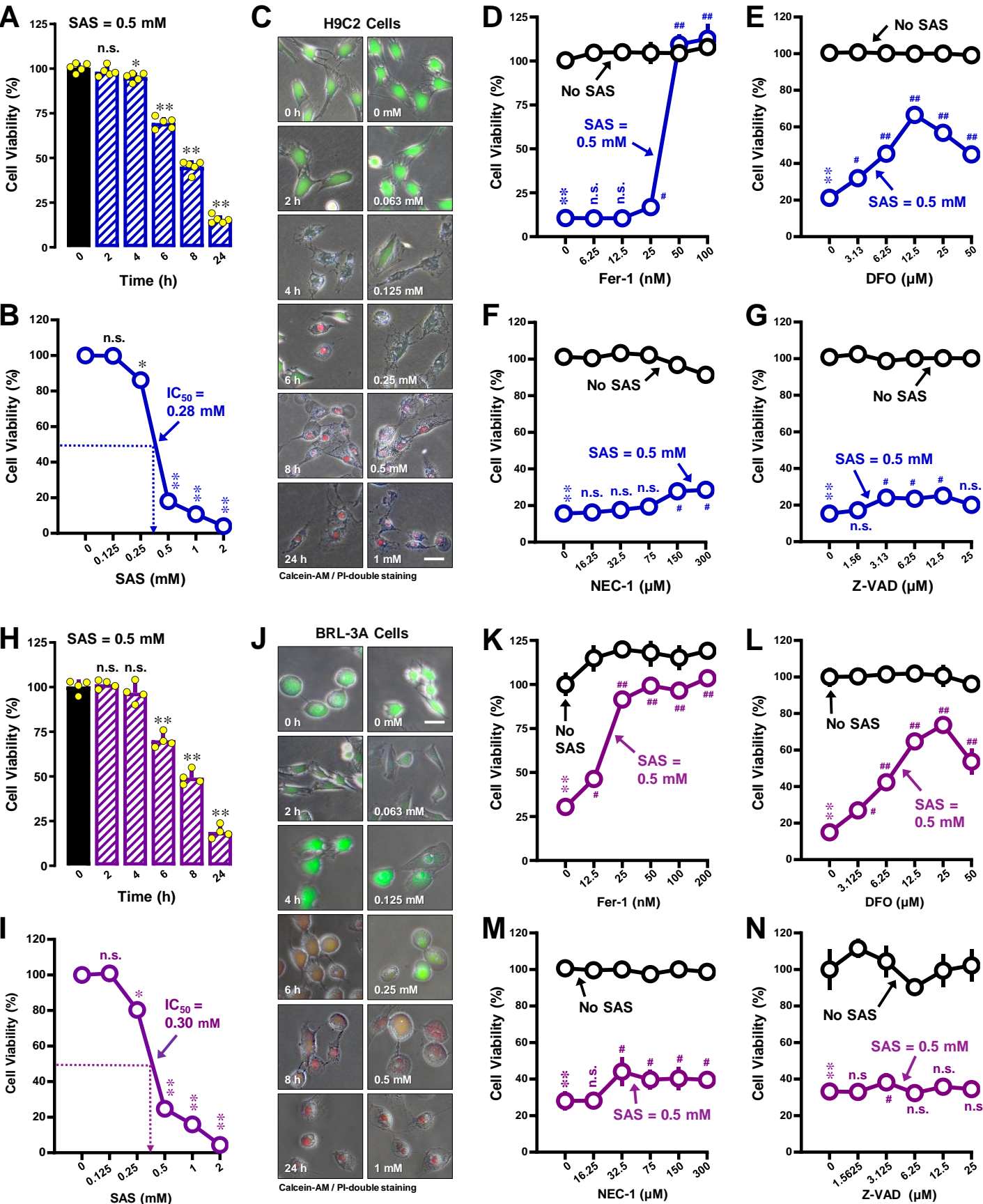

Figure 2

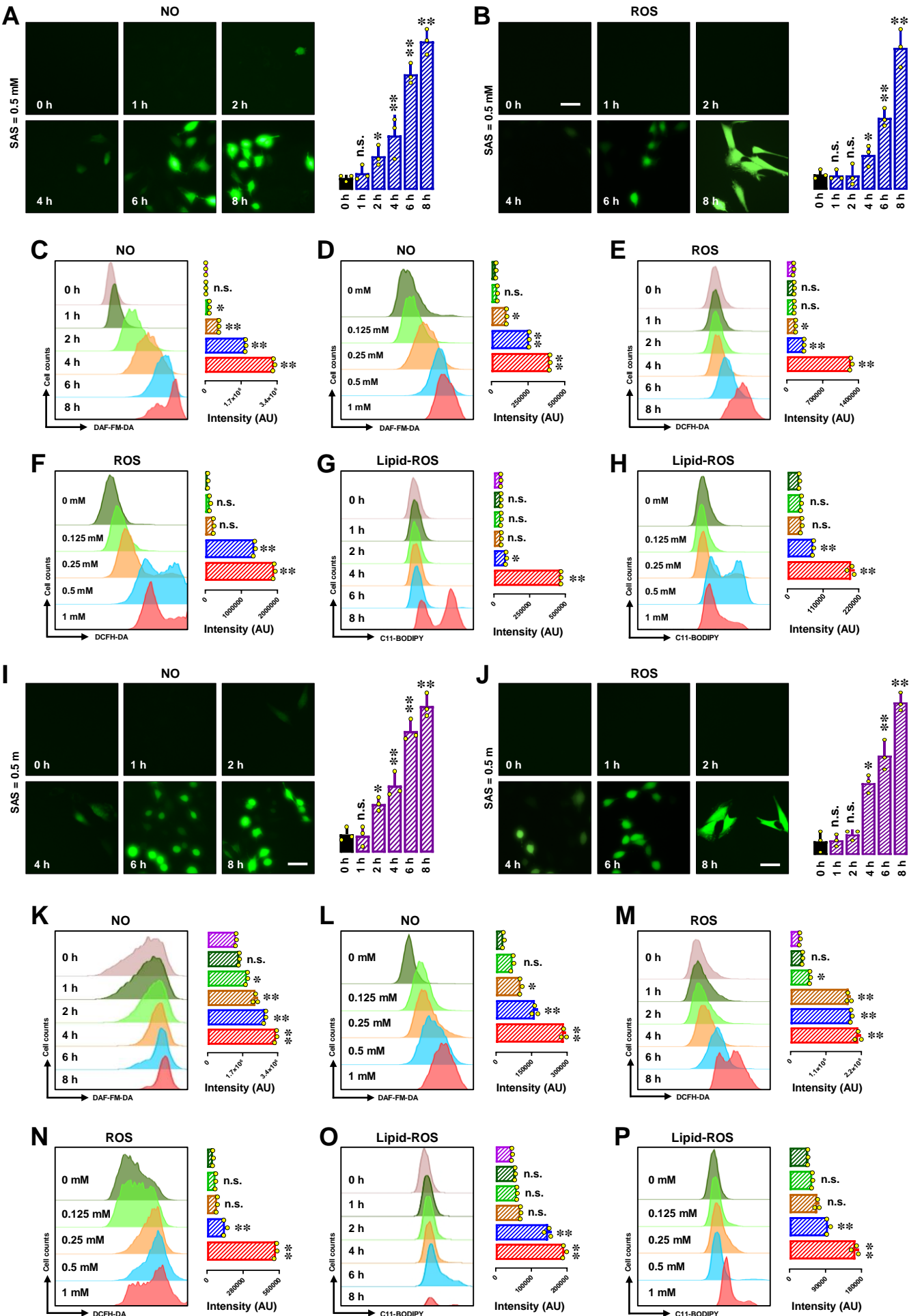

Figure 3

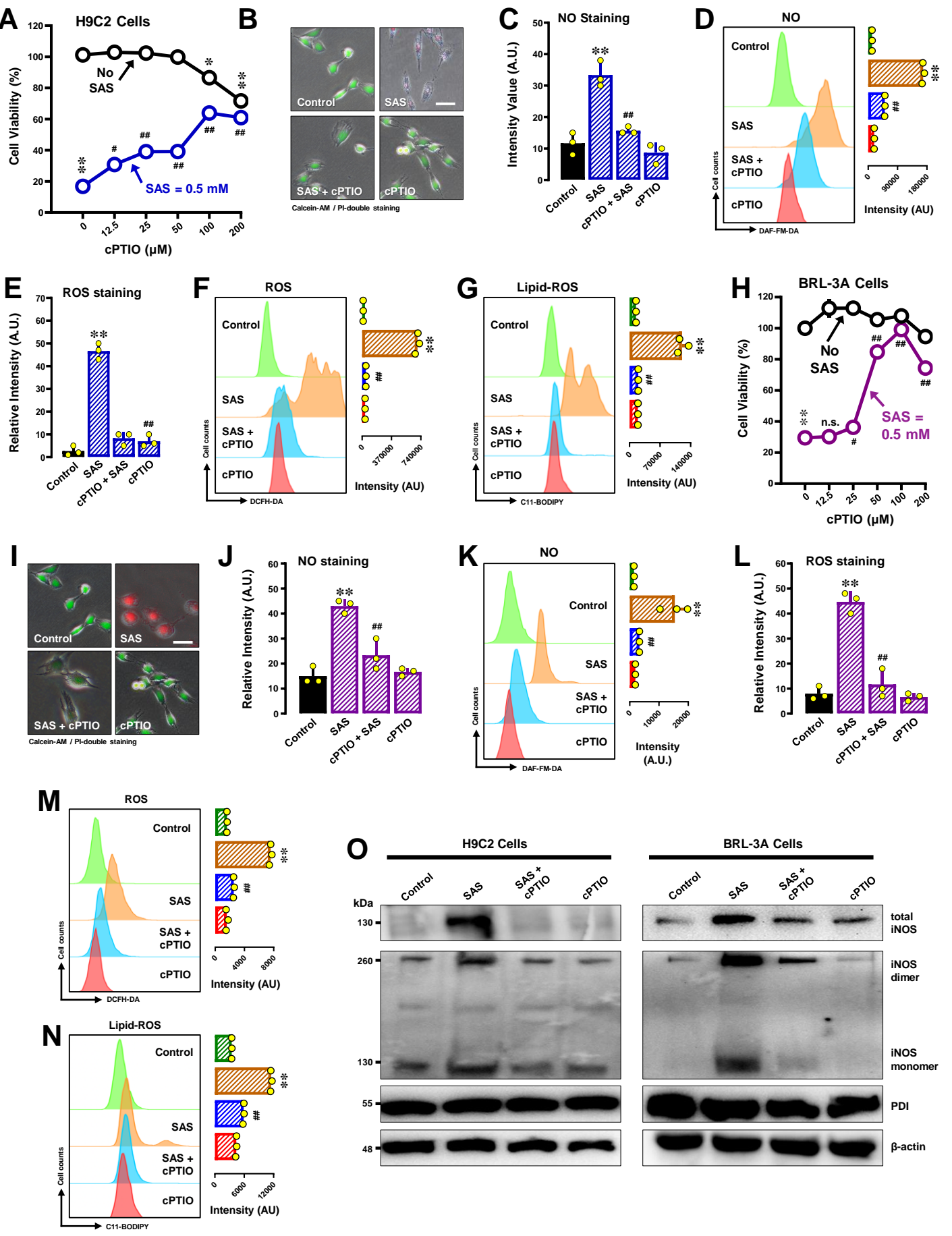

Figure 4

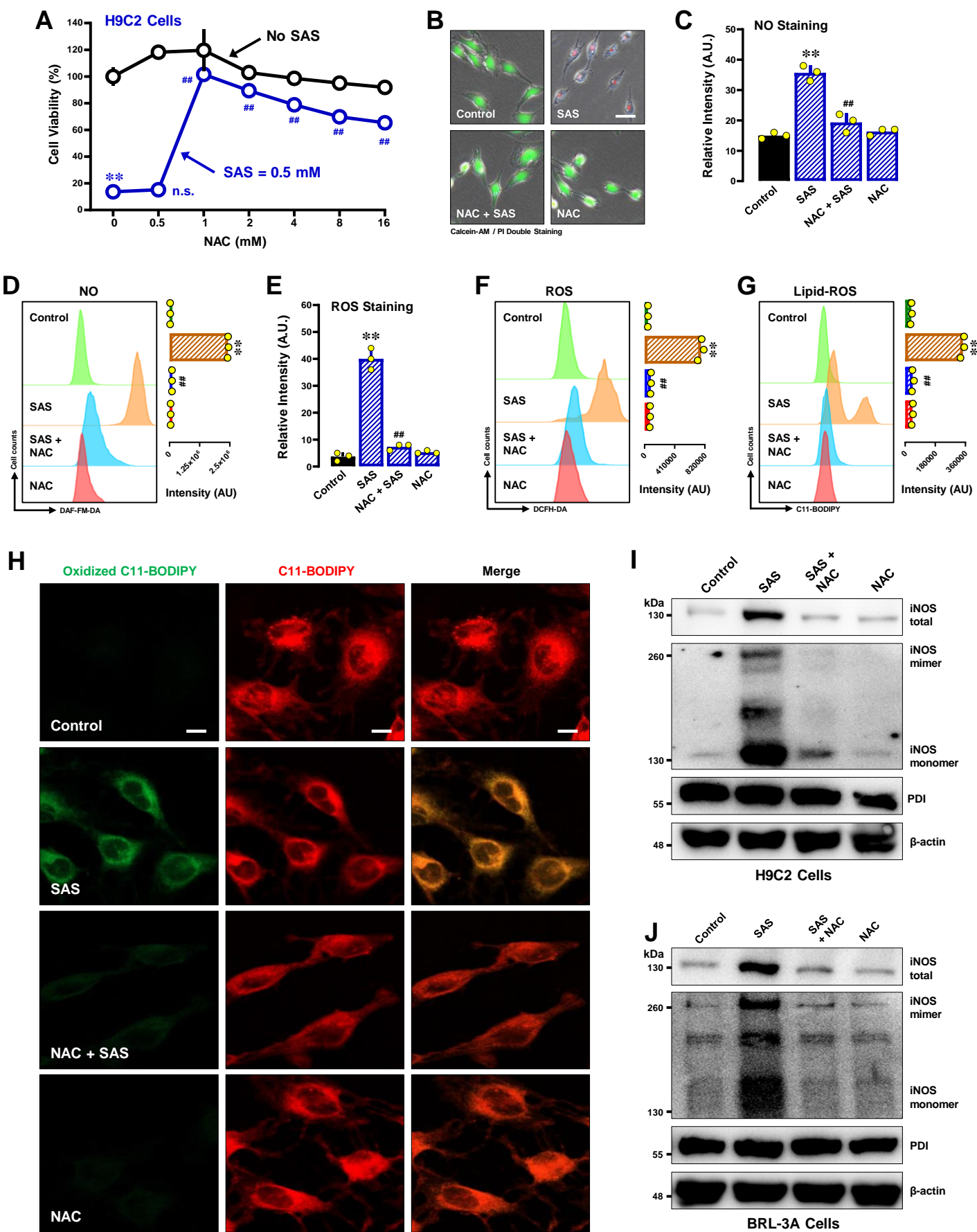

Figure 5

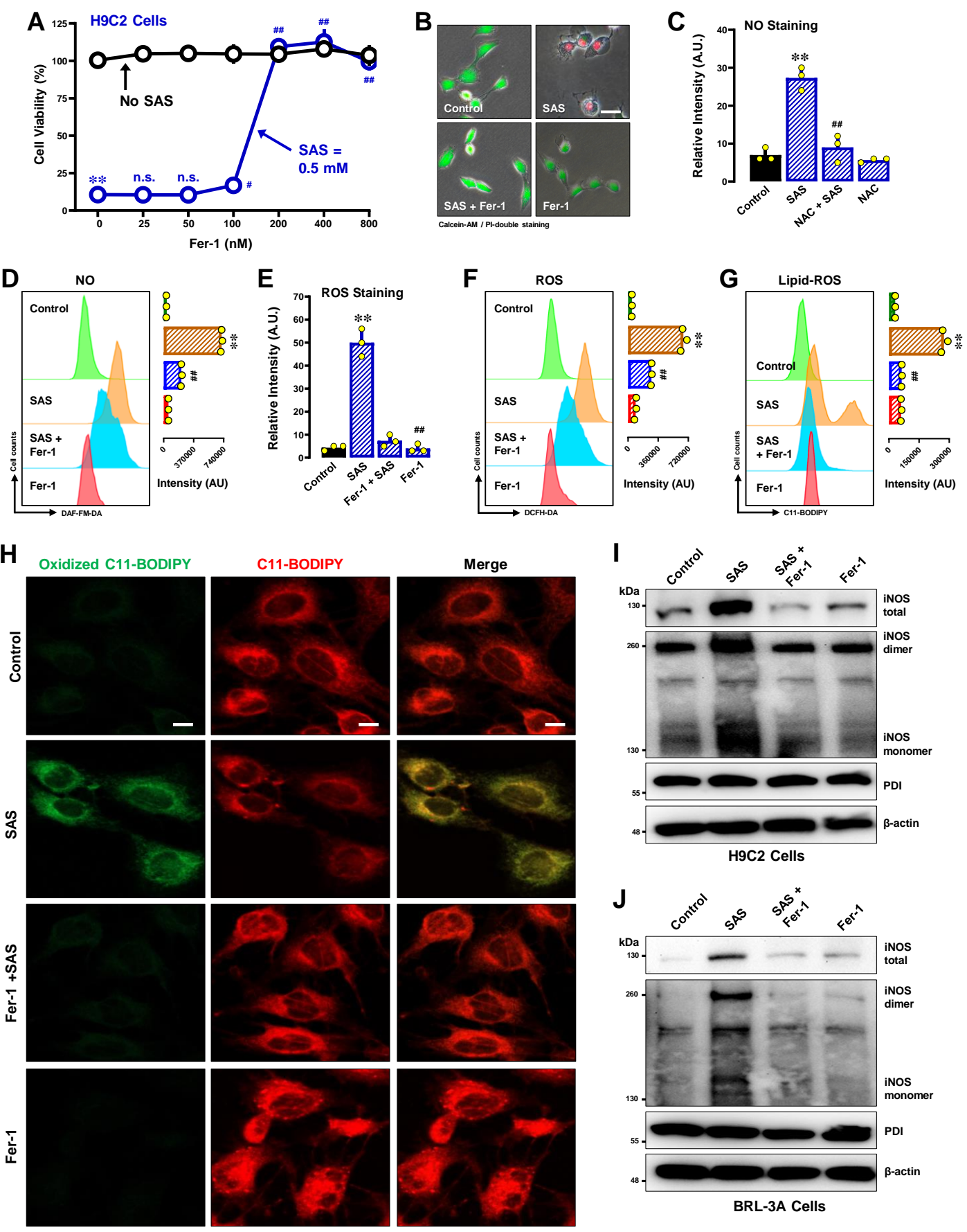

Figure 6

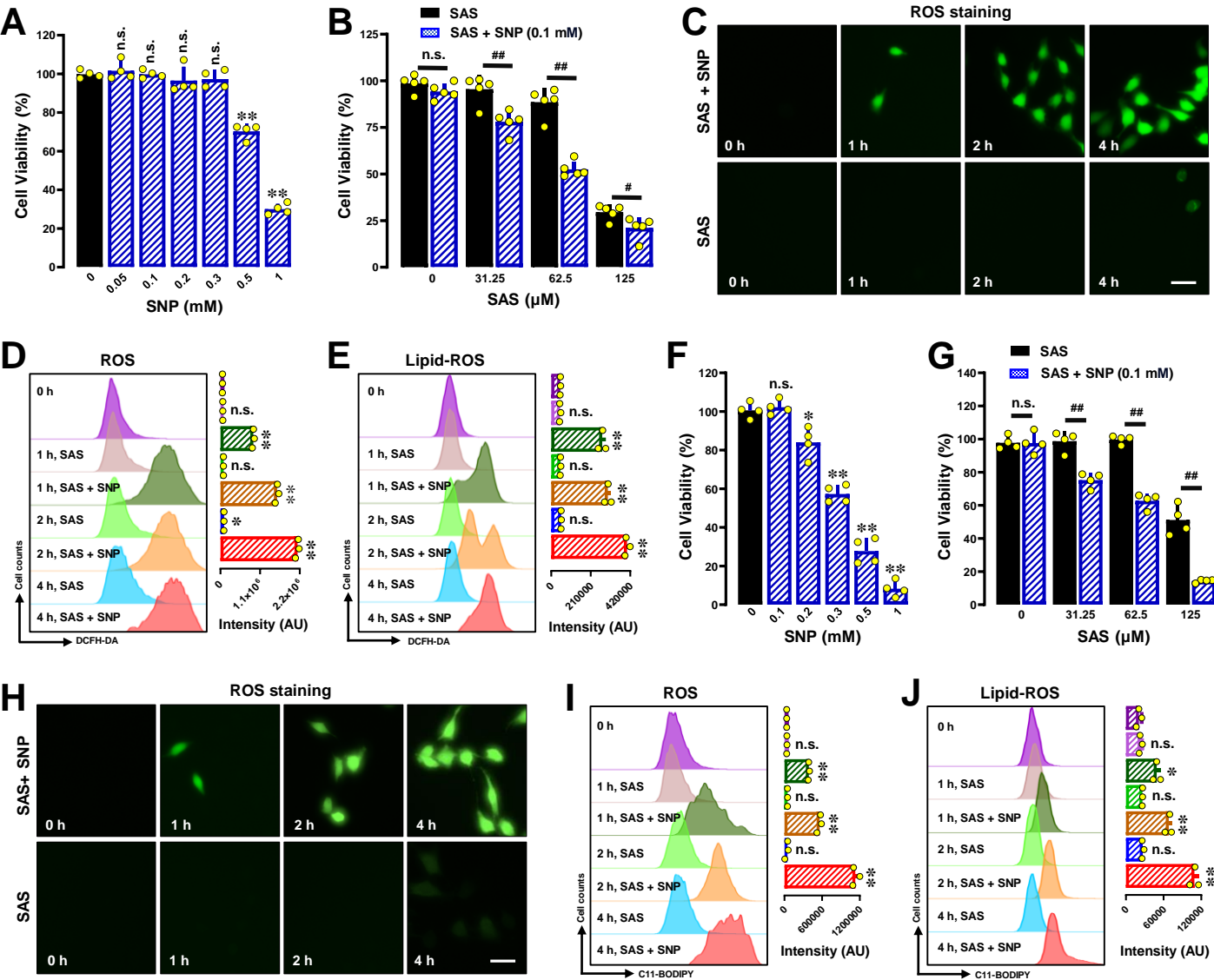

Figure 7

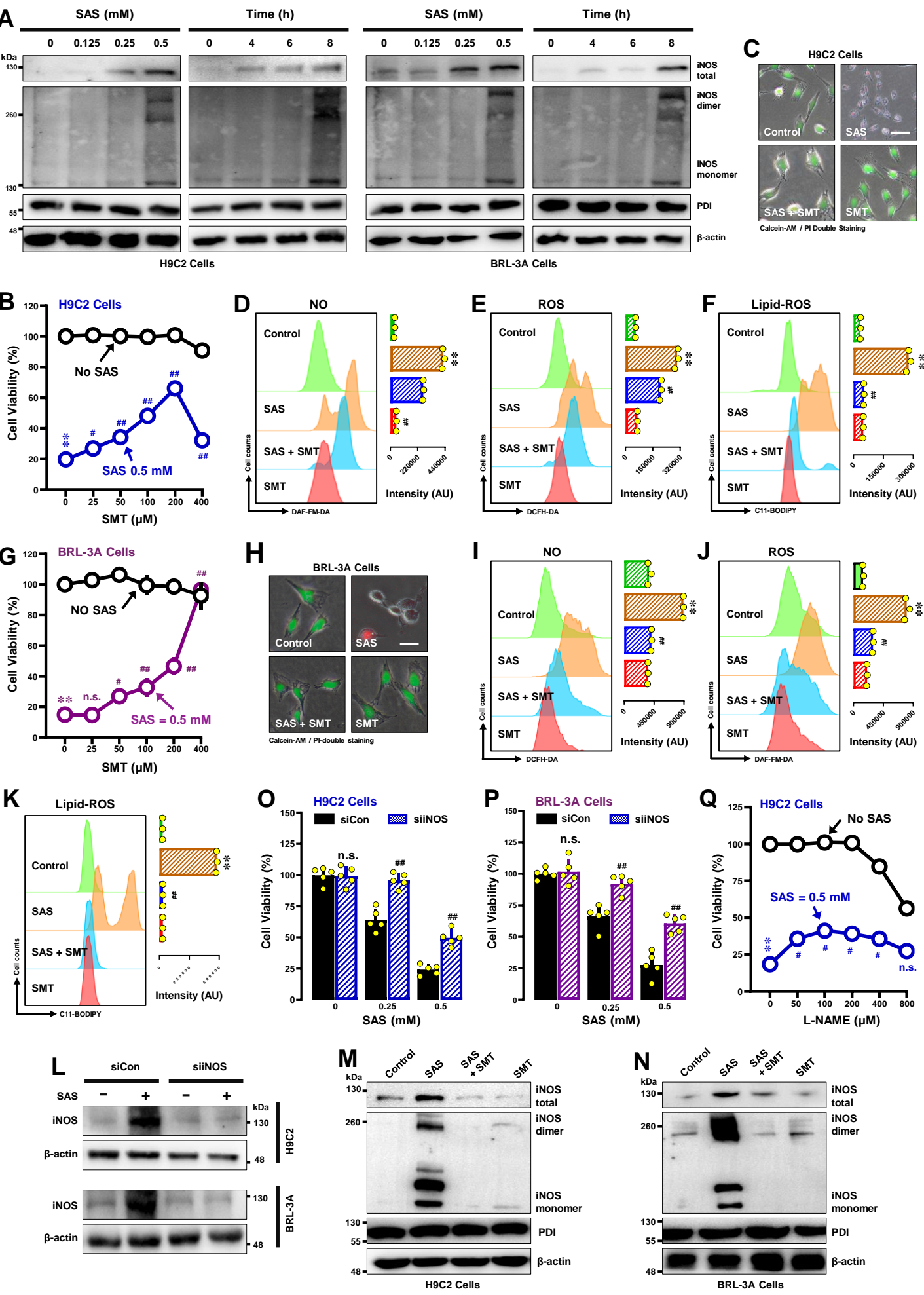

Figure 8

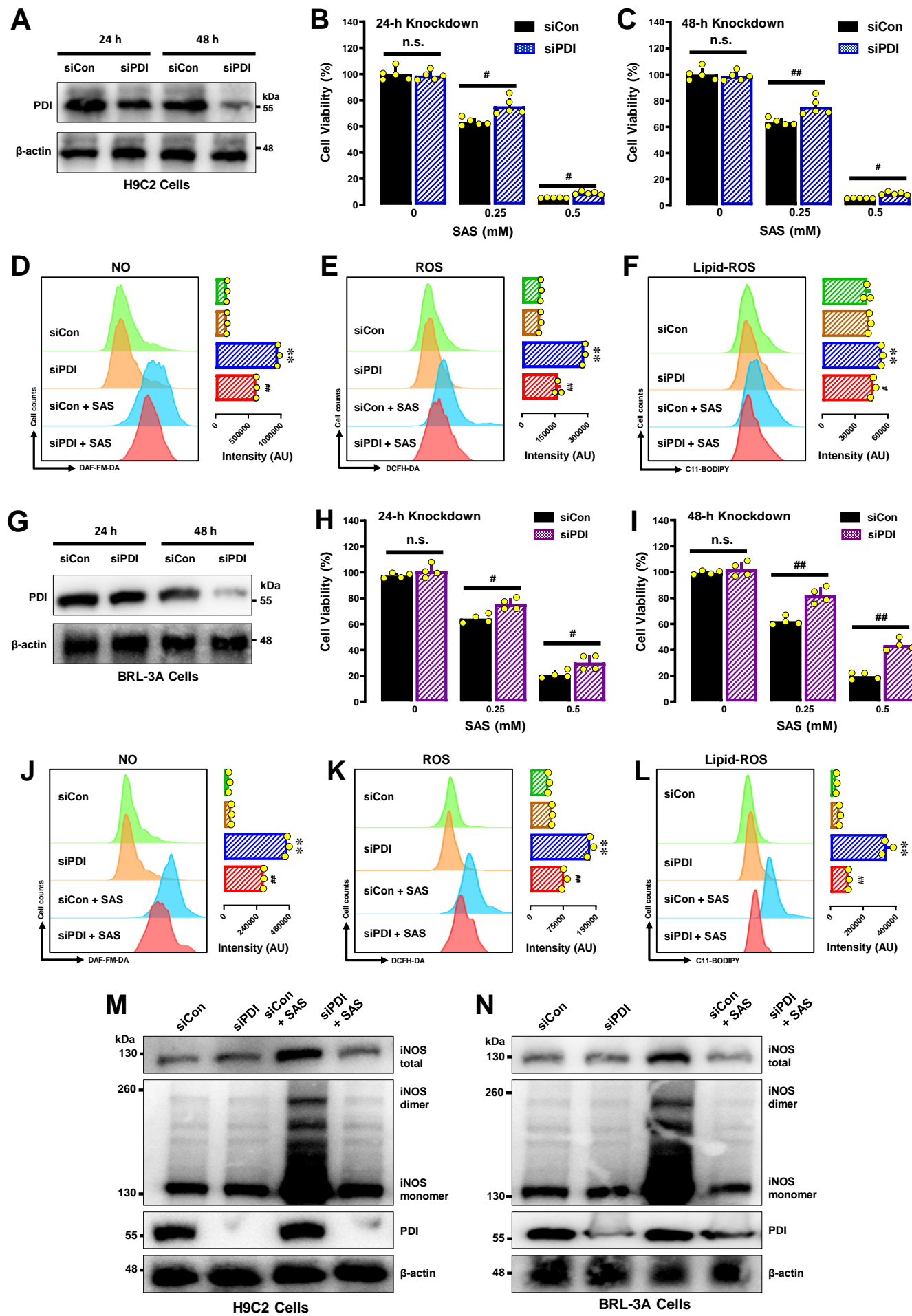

Figure 9

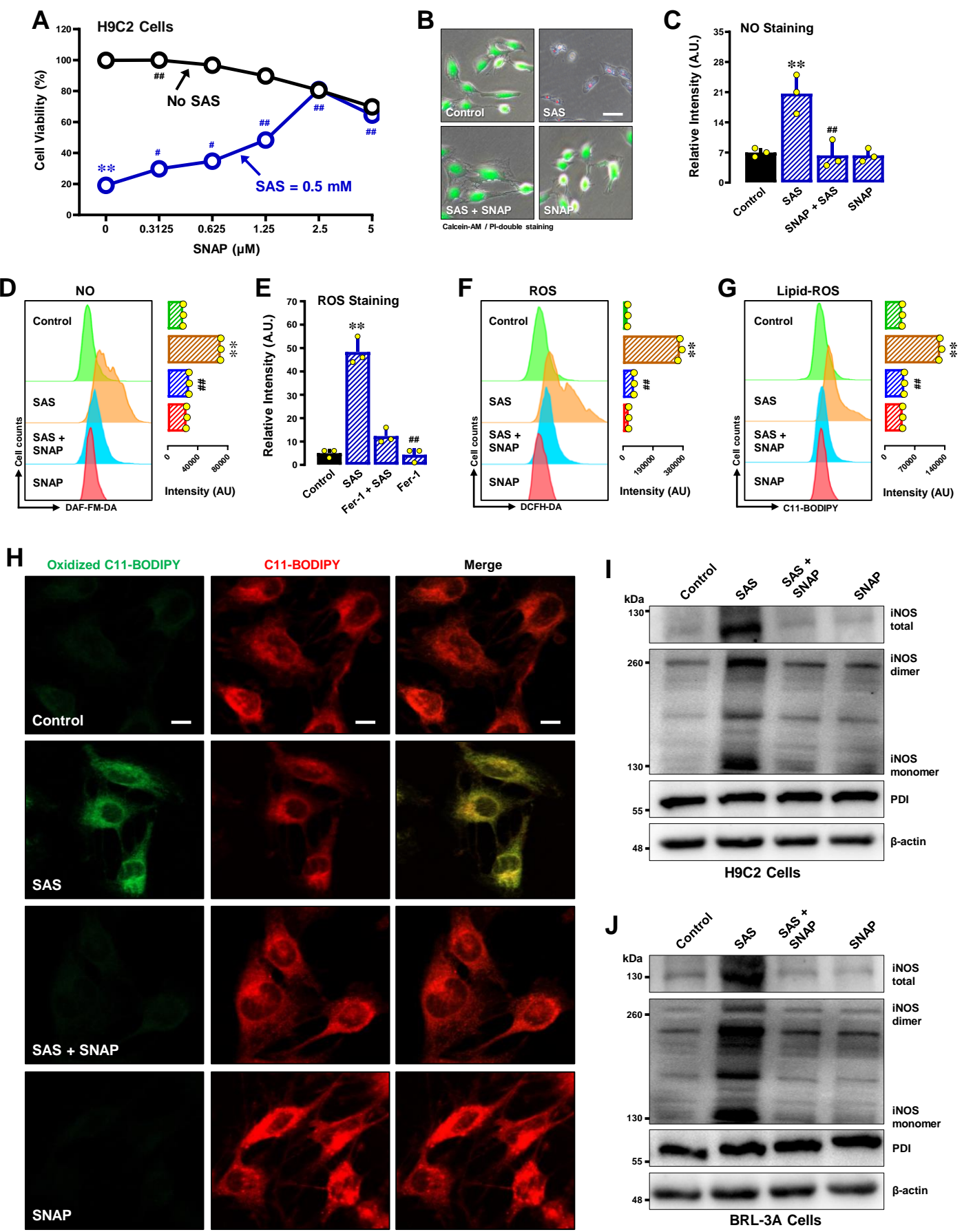

Figure 10

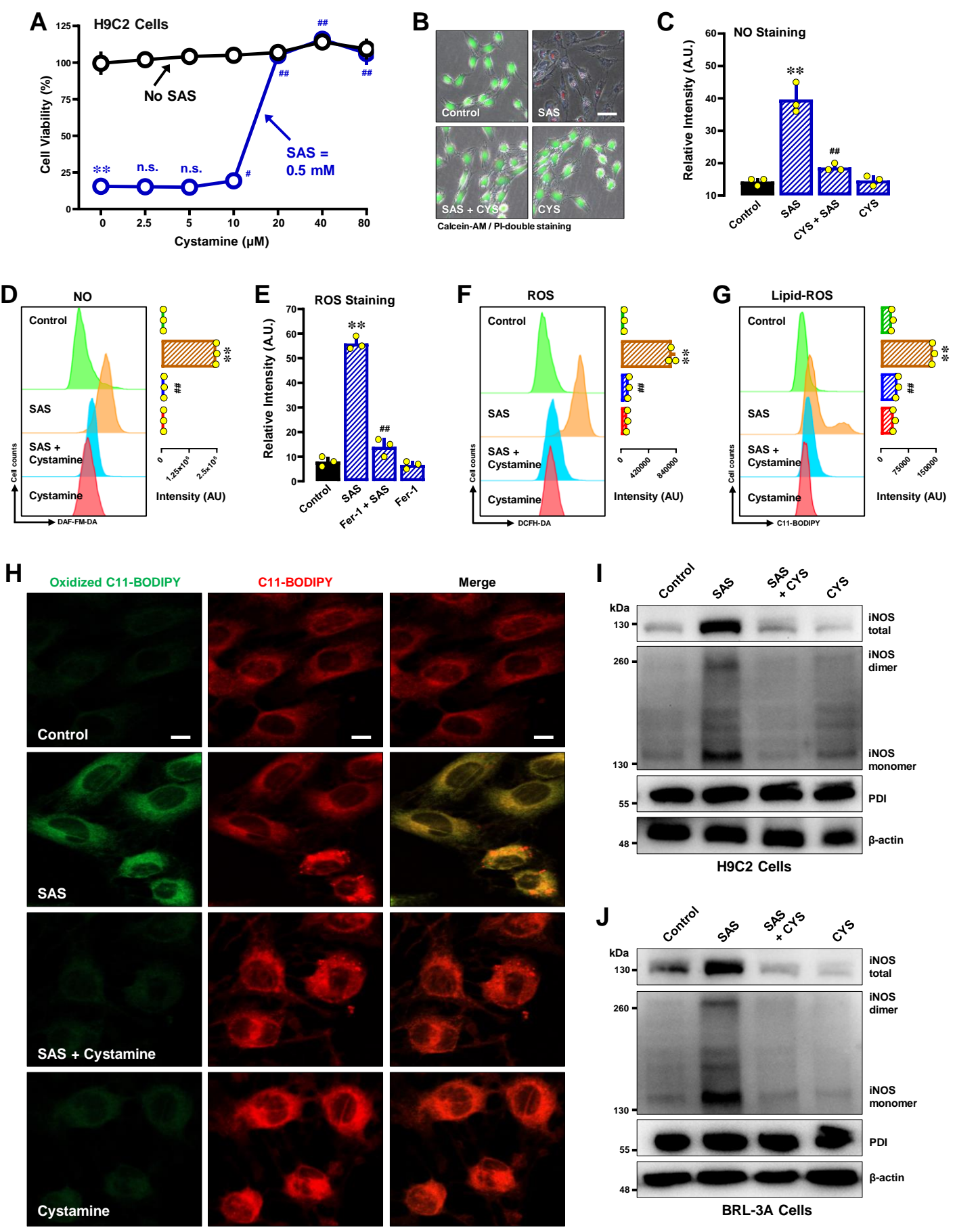

Figure 11

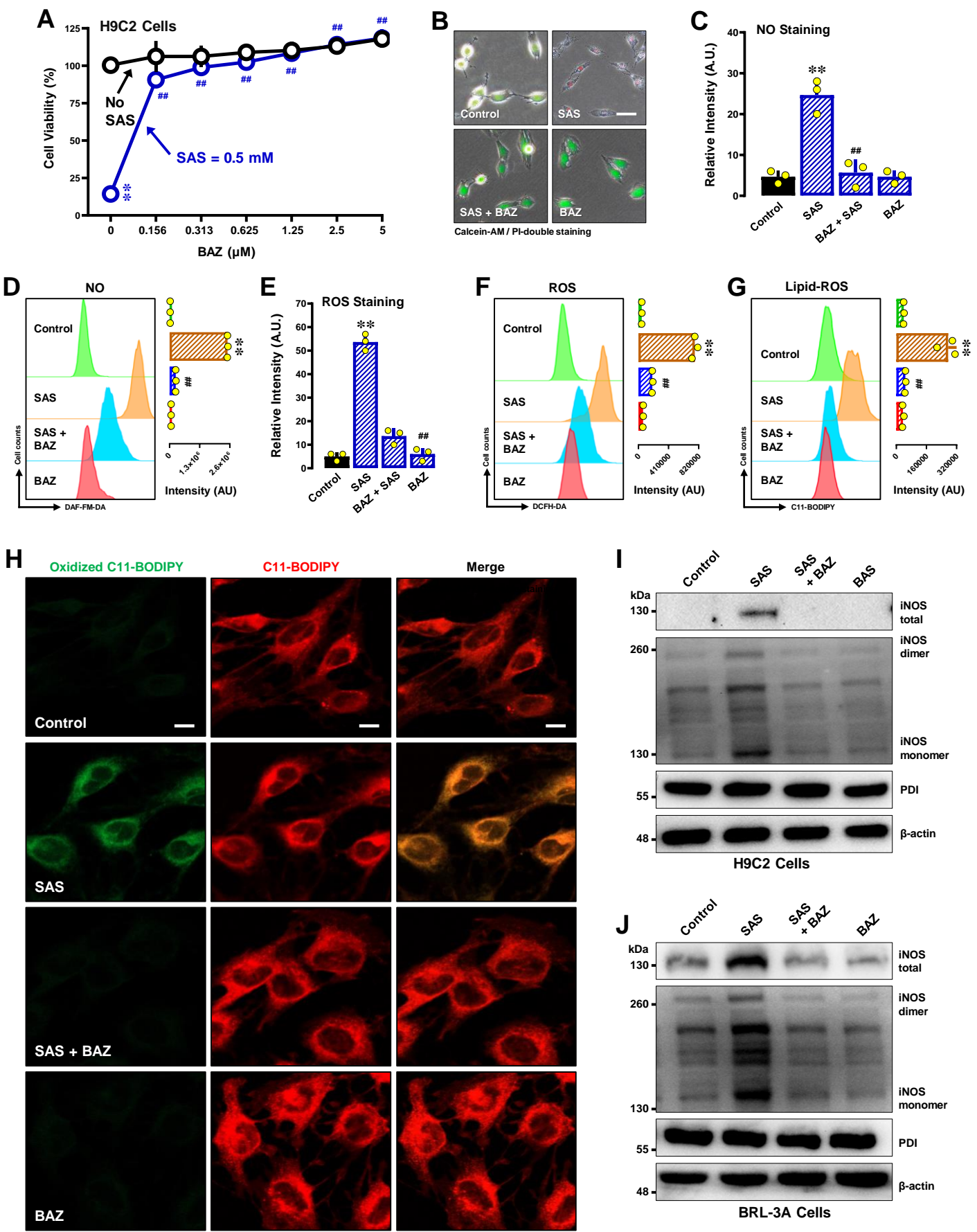

Figure 12

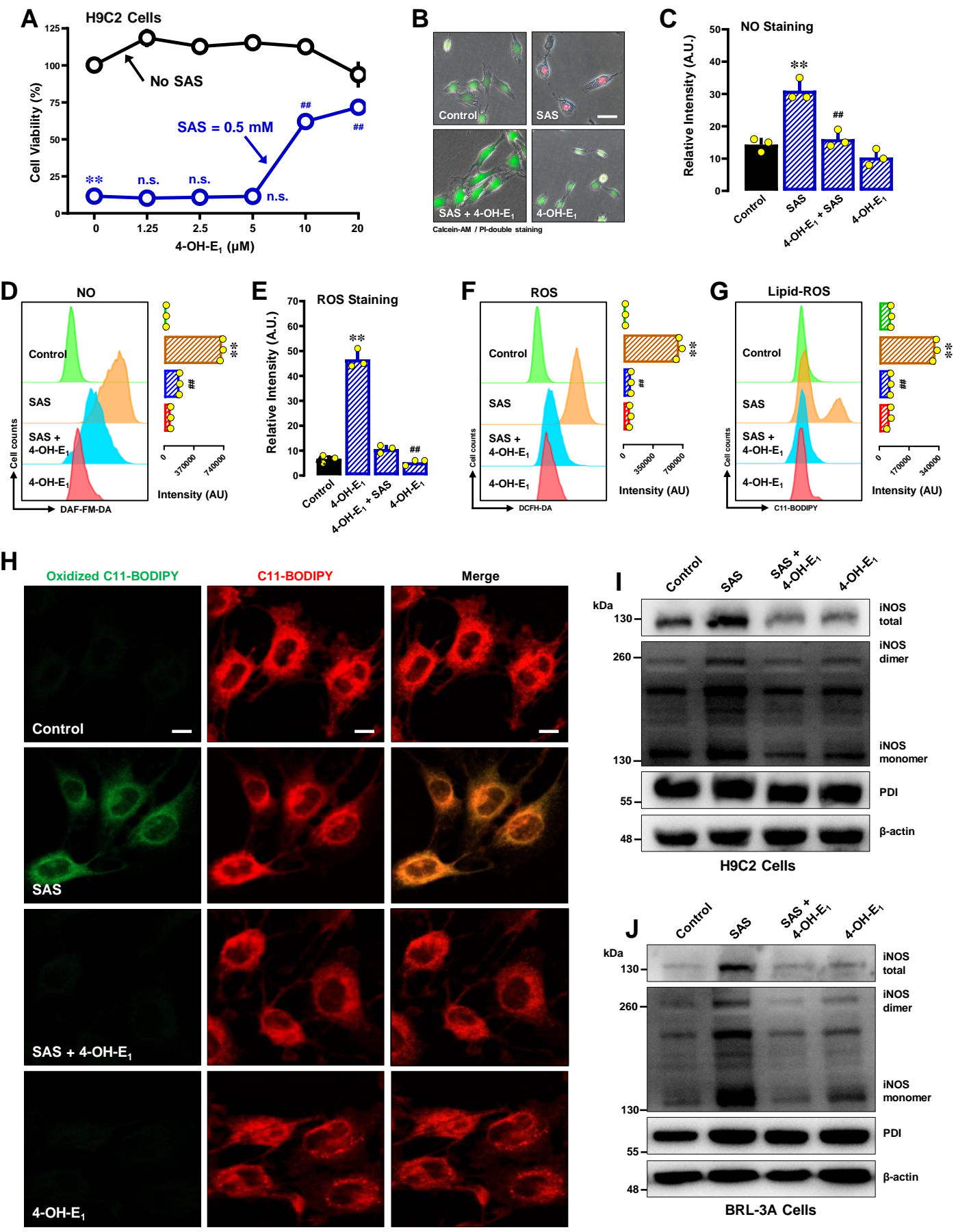

Supplement: JYC3_Figures_Final_2025-4-10 [file JYC3_Figures_Final_2025-4-10.pdf]
