# Supplementary material for: Characterization of the mechanisms underlying sulfasalazine-induced ferroptotic cell death: role of protein disulfide isomerase-mediated NOS activation and NO accumulation: Mechanism of SAS-induced cell death
Source: Acta Biochim Biophys Sin (Shanghai). 2025 Aug 21;57(12):2074–93. doi: 10.3724/abbs.2025100 (PMC12747976; doi:10.3724/abbs.2025100)
Supplement: Supplementary_Figures_Final_2025-4-10 [file Supplementary_Figures_Final_2025-4-10.pdf]

Supplementary Figure S1

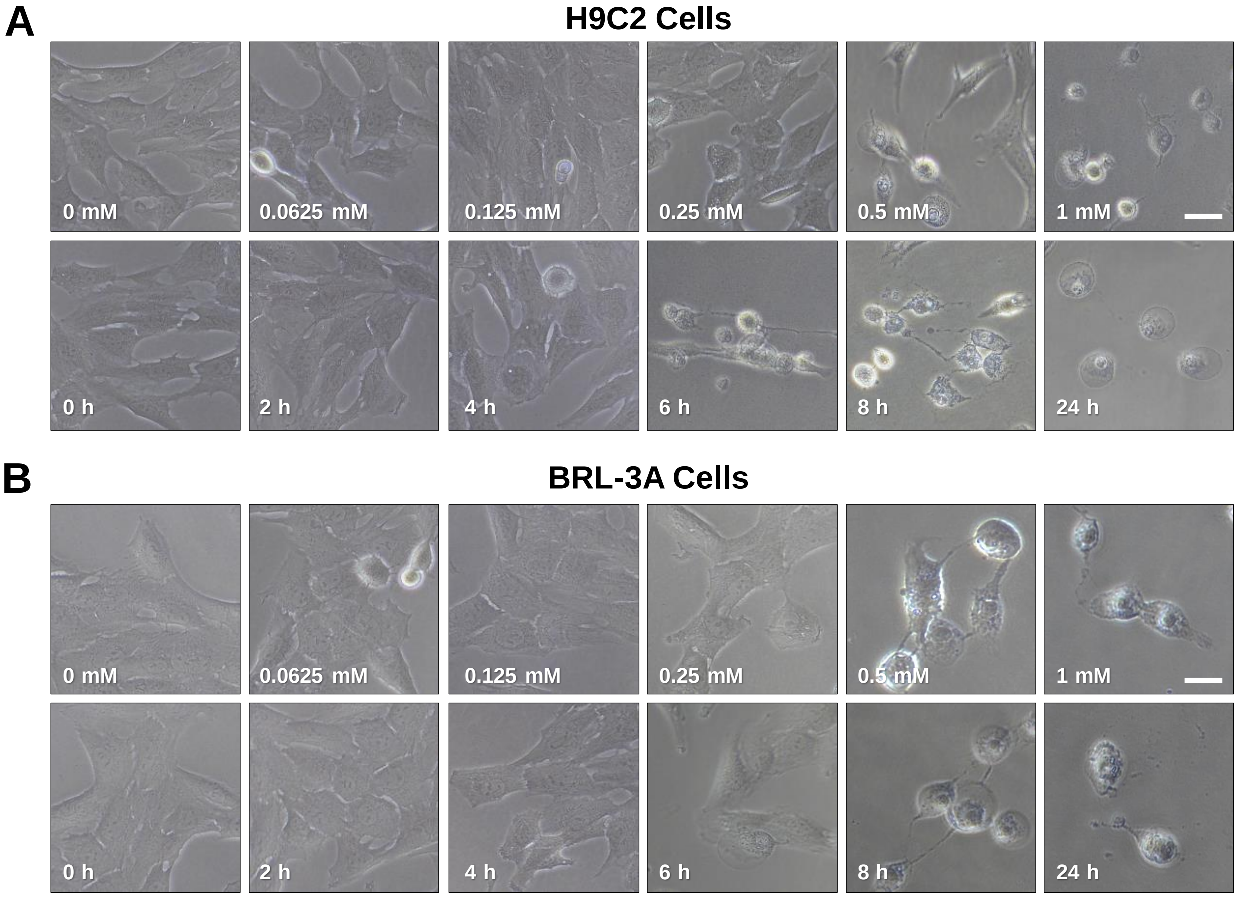

Supplementary Fig. S1. Concentration- and time-dependent induction of ferroptotic cell death by SAS in H9C2 and BRL-3A cells.

A. Concentration- and time-dependent changes in gross morphology of H9C2 cells after exposure to increasing concentrations of SAS for 24 h (**upper panel**) or 0.5 mM SAS for varying durations (**lower panel**). The cellular images were captured with a light microscope (40×, scale bar = 100 μm).

B. Concentration- and time-dependent changes in gross morphology of BRL-3A cells after exposure to increasing concentrations of SAS for 24 h (**upper panel**) or 0.5 mM SAS for varying durations (**lower panel**). The cellular images were captured with a light microscope (40×, scale bar = 100 μm).

Supplementary Figure S2

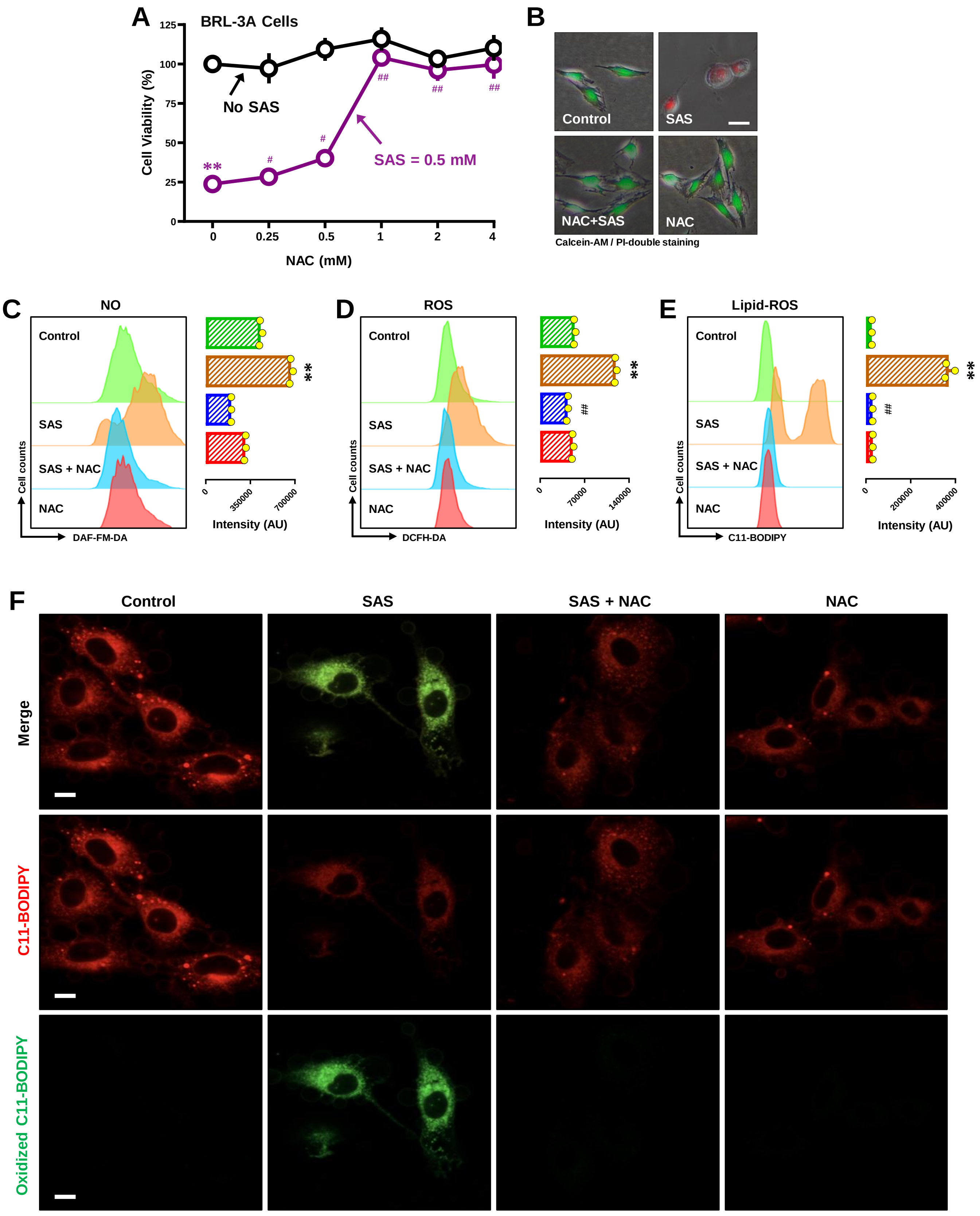

Supplementary Fig. S2. Effect of NAC on SAS-induced ferroptosis and accumulation of NO, ROS and lipid-ROS in BRL-3A cells.

A, B. Protective effect of NAC against SAS-induced cytotoxicity. In **A**, cells were treated with SAS (0.5 mM) ± NAC (0.125, 0.25, 0.5, 2, 2 and 4 mM) for 24 h, and then subjected to MTT assay (n = 4). In **B**, cells were treated with SAS (0.5 mM) ± NAC (0.5 mM) for 24 h, and then fluorescent images of Calcein-AM/PI-stained cells were captured (green for live cells, and red for dead cells; scale bar = 100 μm).

C, D, E. Abrogation by NAC of SAS-induced accumulation of cellular NO (**C**), ROS (**D**) and lipid-ROS (**E**). Cells were treated with SAS (0.5 mM) ± NAC (0.5 mM) for 8 h, and then subjected to analytical flow cytometry. The left panels are the histograms, and the right panels are the quantitative values (n = 3).

F. Abrogation by SNAP of SAS-induced accumulation of cellular lipid-ROS (confocal microscopy, scale bar = 100 μm).

Quantitative data are presented as mean ± SD. * or $^{\#}$ $P < 0.05$; ** or $^{\#\#}$ $P < 0.01$; n.s., not significant.

Supplementary Figure S3

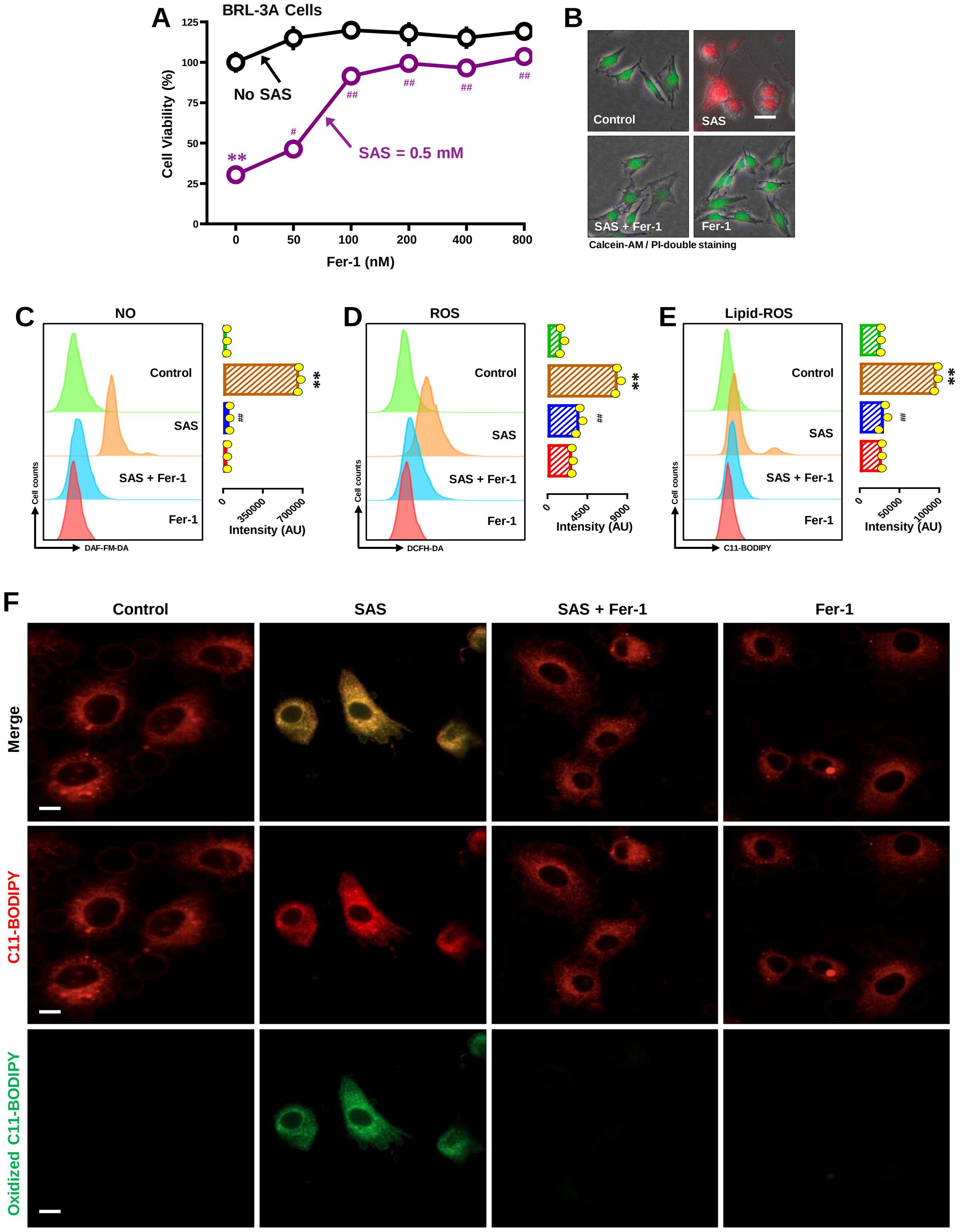

Supplementary Fig. S3. Effect of Fer-1 on SAS-induced ferroptosis and accumulation of NO, ROS and lipid-ROS in BRL-3A cells.

A, B. Protective effect of Fer-1 against SAS-induced cytotoxicity. In **A**, cells were treated with SAS (0.5 mM) ± Fer-1 (25, 50, 100, 200, 400 and 800 nM) for 24 h, and then cell viability was determined by MTT assay (n = 4). In **B**, cells were treated with SAS (0.5 mM) ± Fer-1 (200 nM) for 24 h, and then fluorescent images of Calcein-AM/PI-stained cells were captured (green for live cells, and red for dead cells; scale bar = 100 μm).

C, D, E. Abrogation by Fer-1 of SAS-induced accumulation of cellular NO (**C, D**), ROS (**E, F**) and lipid-ROS (**G**). Cells were treated with SAS (0.5 mM) ± Fer-1 (200 nM) for 8 h, and then subjected to analytical flow cytometry. The left panels are the histograms, and the right panels are the quantitative values (n = 3).

F. Abrogation by SNAP of SAS-induced accumulation of cellular lipid-ROS (confocal microscopy, scale bar = 100 μm).

Quantitative data are presented as mean ± SD. * or $^{\#}$ $P < 0.05$; ** or $^{\#\#}$ $P < 0.01$; n.s., not significant.

Supplementary Figure S4

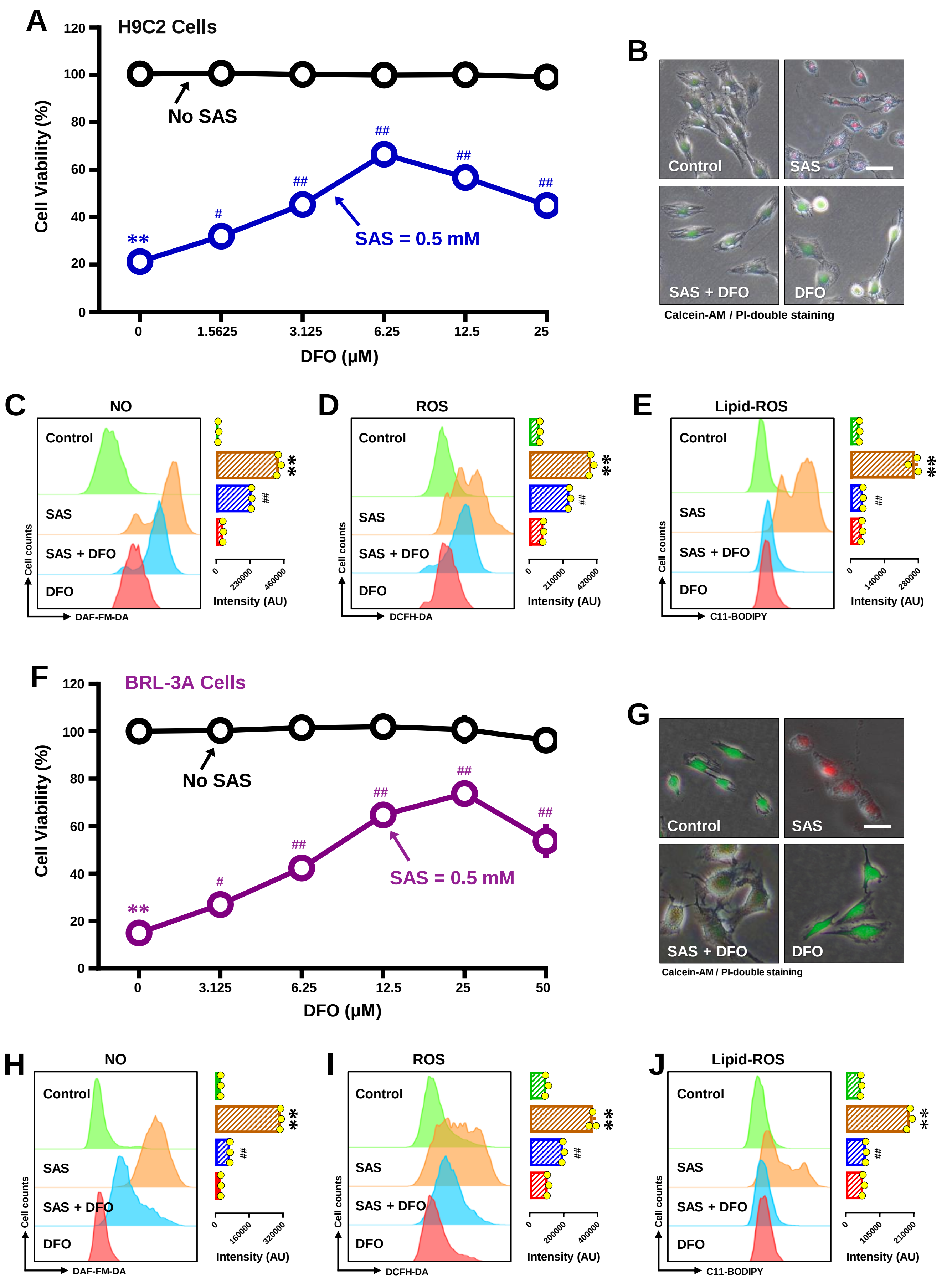

Supplementary Fig. S4. Effect of DFO on SAS-induced ferroptosis and accumulation of NO, ROS and lipid-ROS in H9C2 and BRL-3A cells.

A, B. Effect of DFO against SAS-induced cytotoxicity in H9C2 cells. In **A**, cells were treated with SAS (0.5 mM) $\pm$ DFO (1.563, 3.125, 6.25, 12.5 and 25 μM) for 24 h, and then cell viability was determined by MTT assay (n = 5). In **B**, cells were treated with SAS (0.5 mM) $\pm$ DFO (6.25 μM) for 24 h, and then fluorescent images of Calcein-AM/PI-stained cells were captured (green for live cells, and red for dead cells; scale bar = 100 μm).

C, D, E. Partial abrogation by DFO of SAS-induced accumulation of cellular NO (**C**), ROS (**D**) and lipid-ROS (**E**) in H9C2 cells. Cells were treated with SAS (0.5 mM) $\pm$ DFO (6.25 μM) for 8 h, and then subjected to flow cytometry. The left panels are the histograms, and the right panels are the quantitative values (n = 3).

F, G. Effect of DFO against SAS-induced cytotoxicity in BRL-3A cells. In **F**, cells were treated with SAS (0.5 mM) in the presence or absence of varying concentrations of DFO (3.125, 6.25, 12.5, 25, and 50 μM) for 24 h, and cell viability was assessed using the MTT assay (n = 5). In **G**, cells were treated with SAS (0.5 mM) $\pm$ DFO (25 μM) for 24 h, followed by fluorescence imaging of Calcein-AM/PI-stained cells, where green indicates live cells and red indicates dead cells (scale bar = 100 μm).

H, I, J. DFO suppresses SAS-induced accumulation of cellular NO (**H**), ROS (**I**), and lipid-ROS (**J**) in BRL-3A cells. Cells were treated with SAS (0.5 mM) $\pm$ DFO (25 μM) for 8 h, followed by flow cytometry analysis. The left panels are the histograms, and the right panels are the quantitative values (n = 3).

Quantitative data are presented as mean $\pm$ SD. * or $^{\#}$ $P < 0.05$; ** or $^{\#\#}$ $P < 0.01$; n.s., not significant.

Supplementary Figure S5

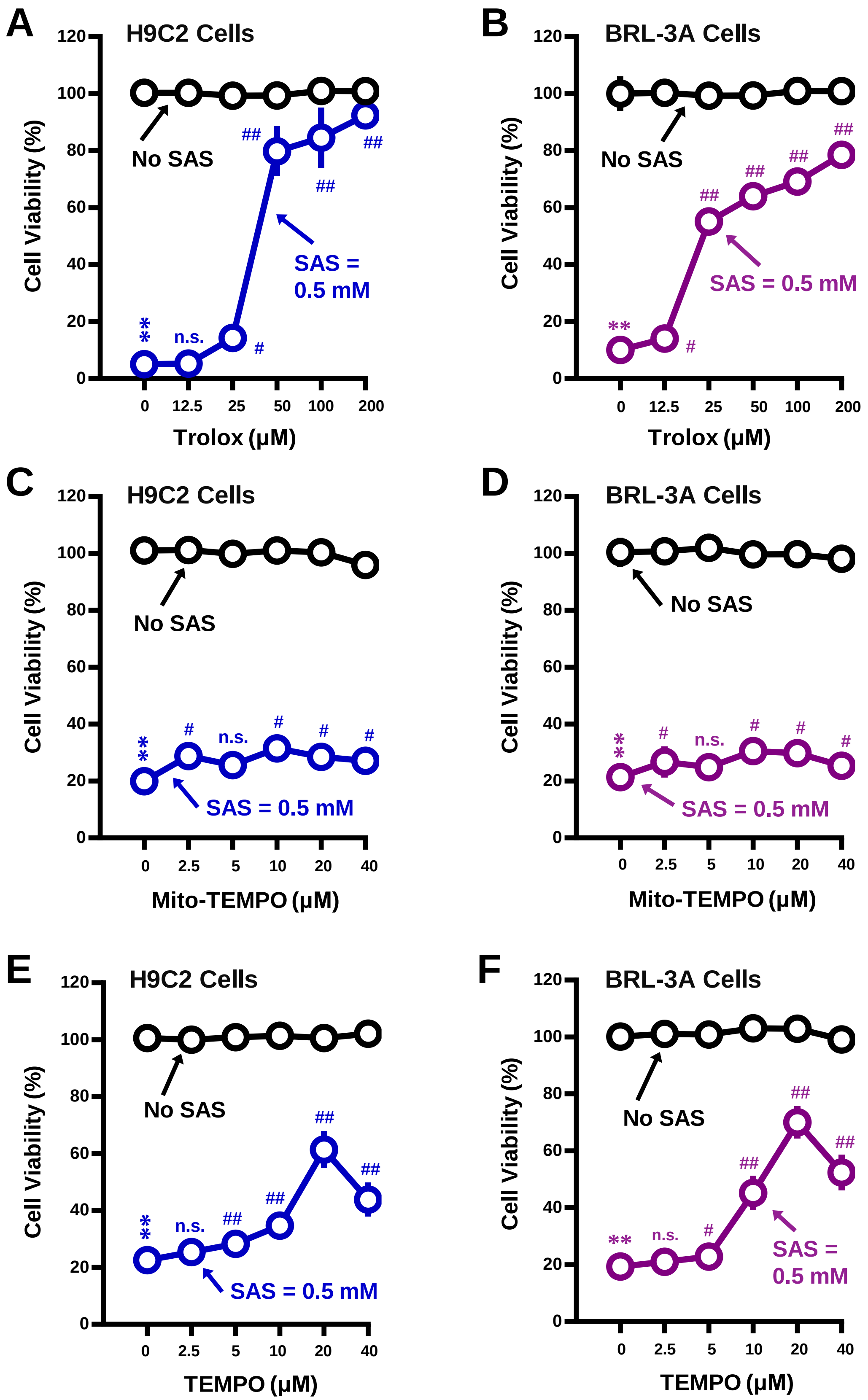

Supplementary Fig. S5. Effect of Trolox, MitoTEMPO and TEMPO on SAS-induced death in H9C2 and BRL-3A cells.

A, B. Protective effect of Trolox against SAS-induced cytotoxicity following treatment of H9C2 (**A**) and BRL-3A (**B**) cells with SAS (0.5 mM) ± Trolox (12.5, 25, 50, 100 and 200 μM) for 24 h (MTT assay, n = 5).

C, D. Lack of a protective effect of MitoTEMPO against SAS cytotoxicity. Cells were treated with SAS (0.5 mM) ± MitoTEMPO (2.5, 5 10, 20 and 40 μM) for 24 h, and cell viability was determined by MTT assay (n = 5).

E, F. Partial protective effect of TEMPO against SAS cytotoxicity. Cells were treated with SAS (0.5 mM) ± TEMPO (2.5, 5 10, 20 and 40 μM) for 24 h, and cell viability was determined by MTT assay (n = 5).

Quantitative data are presented as mean ± SD. * or $^{\#}$ $P < 0.05$; ** or $^{\#\#}$ $P < 0.01$; n.s., not significant.

Supplementary Figure S6

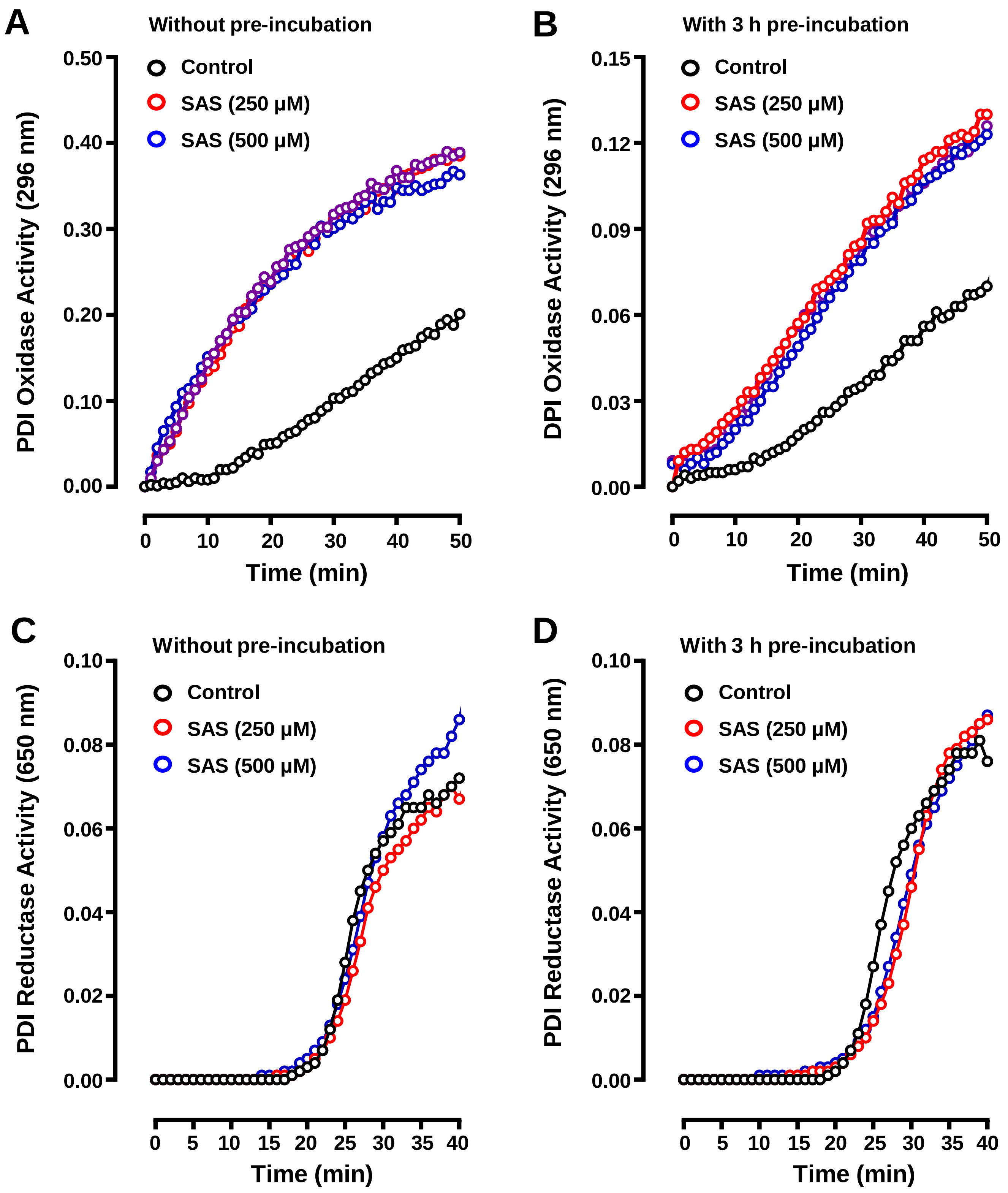

Supplementary Fig. S6. Effect of SAS on the oxidase and reductase activities of PDI in the *in-vitro* enzymatic assays.

A, B. Effect of SAS (at 250 and 500 µM) on the oxidase activity of PDI (**A, B**). The oxidase activity of PDI was assayed by measuring PDI-mediated RNase A refolding *in vitro*. The assay was repeated to confirm the observations. The data from one representative assay is shown.

A, B. Effect of SAS (at 250 and 500 µM) of the reductase activity of PDI. The reductase activity of PDI was assayed by measuring PDI-mediated insulin aggregation *in vitro*. The assay was repeated to confirm the observations. The data from one representative assay is shown.

Supplementary Figure S7

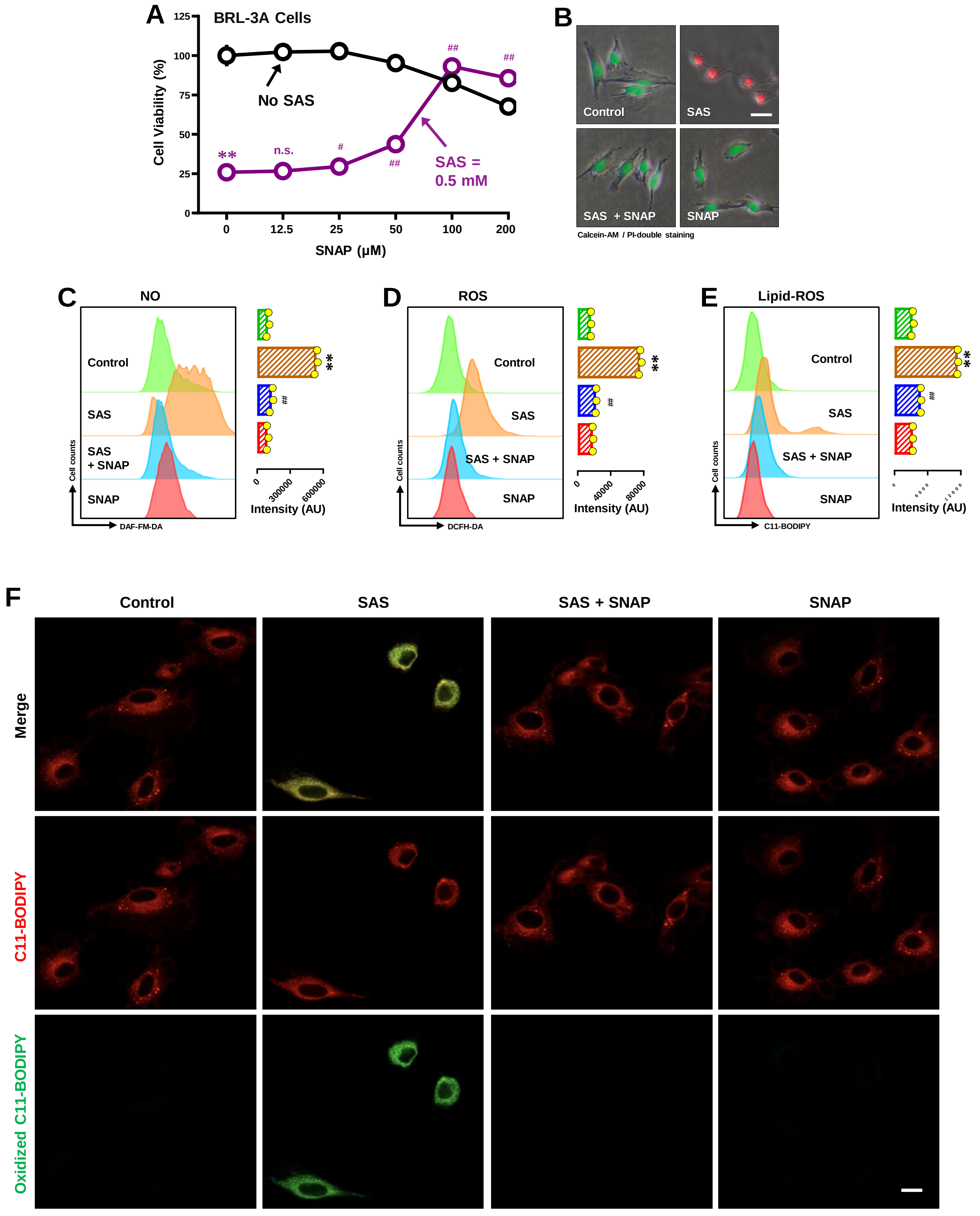

Supplementary Fig. S7. Effect of SNAP on SAS-induced ferroptosis and accumulation of NO, ROS and lipid-ROS in BRL-3A cells.

A, B. Protective effect of SNAP against SAS-induced cytotoxicity. In **A**, cells were treated with SAS (0.5 mM) ± SNAP (12.5, 25, 50, 100 and 200 μM) for 24 h, and cell viability was determined by MTT assay (n = 5). In **B**, cells were treated with SAS (0.5 mM) ± SNAP (100 μM) for 24 h, and then fluorescent images of Calcein-AM/PI-stained cells were captured (green for live cells, and red for dead cells; scale bar = 100 μm).

C, D, E. Abrogation by SNAP of SAS-induced accumulation of cellular NO (**C**), ROS (**D**) and lipid-ROS (**E**). Cells were treated with SAS (0.5 mM) ± SNAP (100 μM) for 8 h, and then subjected to analytical flow cytometry. The left panels are the histograms, and the right panels are the quantitative values (n = 3).

F. Abrogation by SNAP of SAS-induced accumulation of cellular lipid-ROS (confocal microscopy, scale bar = 100 μm).

Quantitative data are presented as mean ± SD. * or $^{\#}$ $P < 0.05$; ** or $^{\#\#}$ $P < 0.01$; n.s., not significant.

Supplementary Figure S8

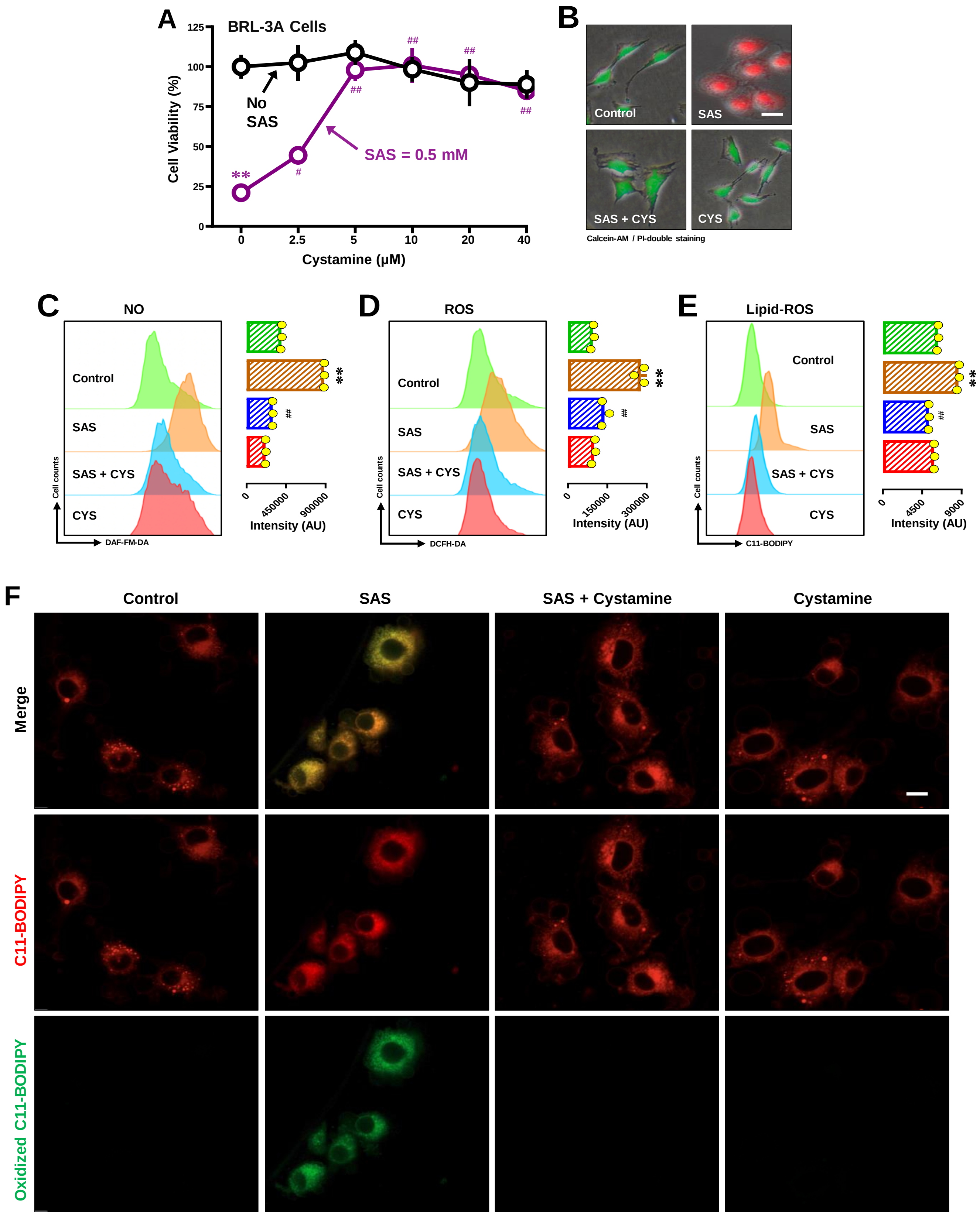

Supplementary Fig. S8. Effect of cystamine on SAS-induced ferroptosis and accumulation of NO, ROS and lipid-ROS in BRL-3A cells.

A, B. Protective effect of cystamine against SAS-induced cytotoxicity. In **A**, cells were treated with SAS (0.5 mM) ± cystamine (2.5, 5, 10, 20 and 40 μM) for 24 h, and cell viability was determined by MTT assay (n = 5). In **B**, cells were treated with SAS (0.5 mM) ± cystamine (10 μM) for 24 h, and then fluorescence images of Calcein-AM/PI-stained cells were captured (green for live cells, and red for dead cells; scale bar = 100 μm).

C, D, E. Abrogation by cystamine of SAS-induced accumulation of cellular NO (**C,**), ROS (**D**) and lipid-ROS (**E**). Cells were treated with SAS (0.5 mM) ± cystamine (10 μM) for 8 h, and then subjected to analytical flow cytometry. The left panels are the histograms, and the right panels are the quantitative values (n = 3).

F. Abrogation by cystamine of SAS-induced accumulation of cellular lipid-ROS (confocal microscopy, scale bar = 100 μm).

Quantitative data are presented as mean ± SD. ** or ## $P < 0.01$; n.s., not significant.

Supplementary Figure S9

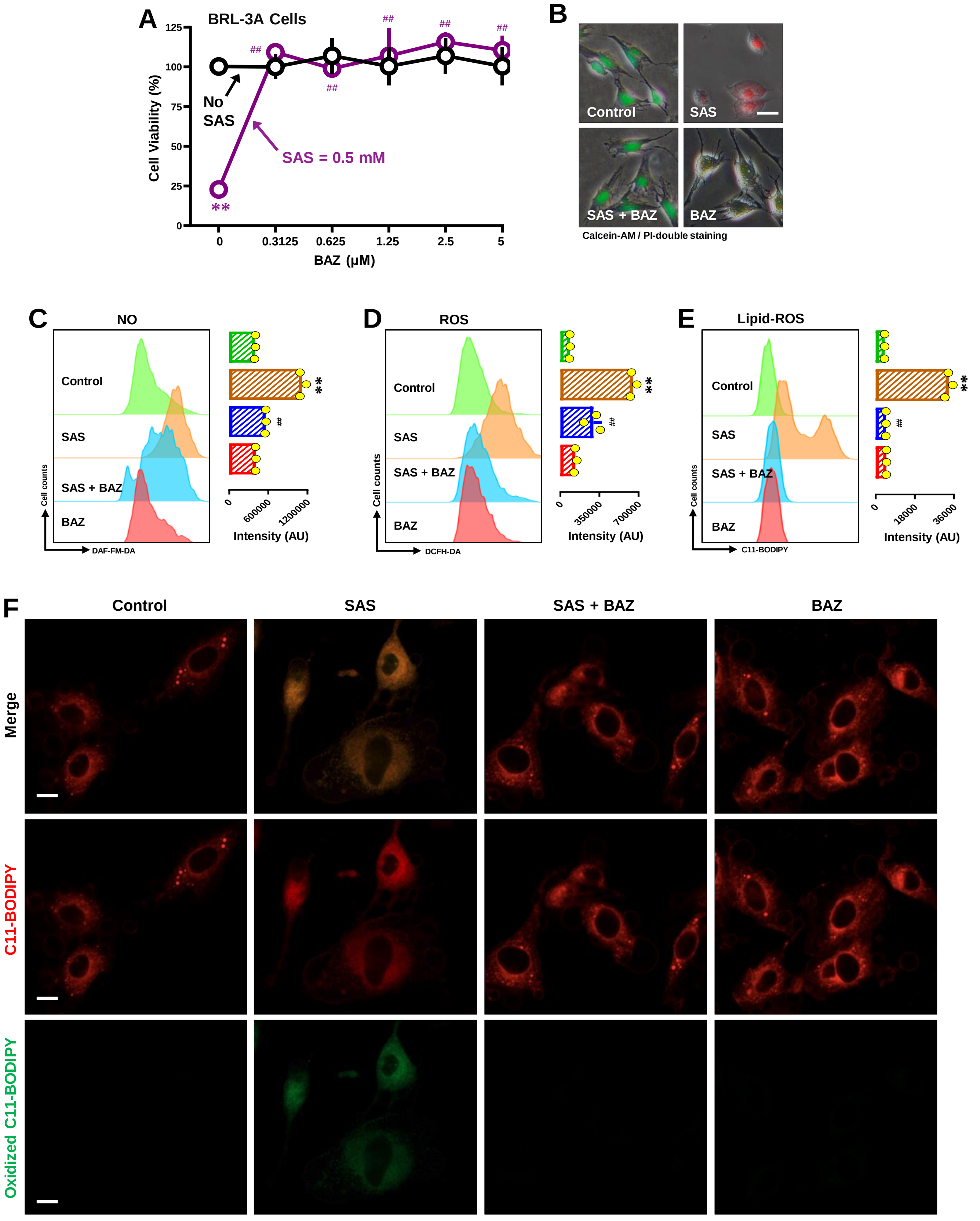

Supplementary Fig. S9. Effect of BAZ on SAS-induced ferroptosis and accumulation of NO, ROS and lipid-ROS in BRL-3A cells.

A, B. Protective effect of BAZ against SAS-induced cytotoxicity. In **A**, cells were treated with SAS (0.5 mM) ± BAZ (0.15625, 0.3125, 0.625, 1.25, 2.5 and 5 μM) for 24 h, and cell viability was determined by MTT assay (n = 5). In **B**, cells were treated with SAS (0.5 mM) ± BAZ (2.5 μM) for 24 h, and then fluorescent images of Calcein-AM/PI-stained cells were captured (green for live cells, and red for dead cells; scale bar = 100 μm).

C, D, E. Abrogation by BAZ of SAS-induced accumulation of cellular NO (**C**), ROS (**D**) and lipid-ROS (**E**). Cells were treated with SAS (0.5 mM) ± BAZ (2.5 μM) for 8 h, and then subjected to analytical flow cytometry. The left panels are the histograms, and the right panels are the quantitative values (n = 3).

F. Abrogation by BAZ of SAS-induced accumulation of cellular lipid-ROS (confocal microscopy, scale bar = 100 μm).

Quantitative data are presented as mean ± SD. ** or $^{\#\#}$ $P < 0.01$; n.s., not significant.

Supplementary Figure S10

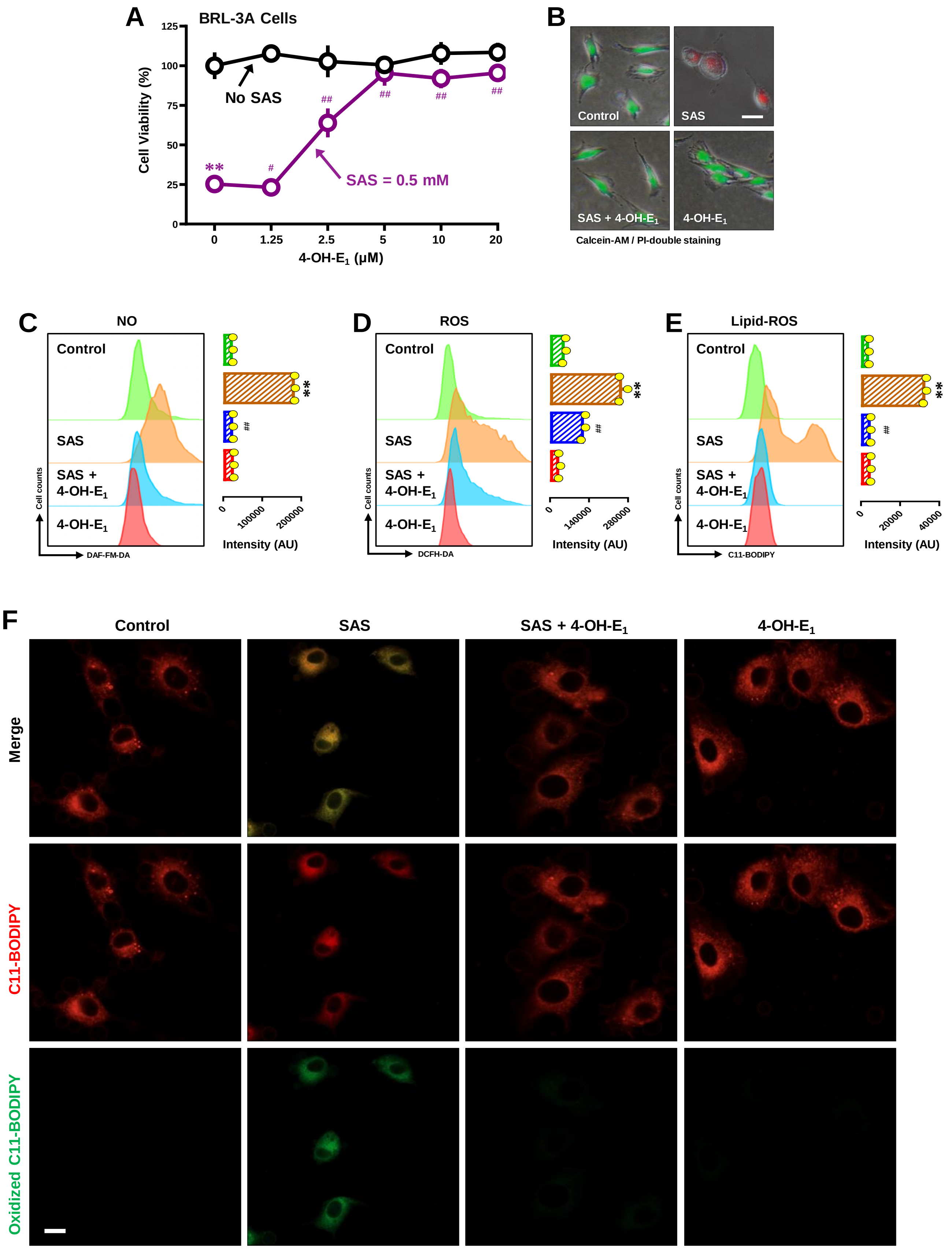

Supplementary Fig. S10. Effect of 4-OH-E_1 on SAS-induced ferroptosis and accumulation of NO, ROS and lipid-ROS in BRL-3A cells.

A, B. Protective effect of 4-OH-E_1 against SAS-induced cytotoxicity. In **A**, cells were treated with SAS (0.5 mM) ± 4-OH-E_1 (1.25, 2.5, 5, 10 and 20 μM) for 24 h, and cell viability was determined by MTT assay (n = 5). In **B**, cells were treated with SAS (0.5 mM) ± 4-OH-E_1 (5 μM) for 24 h, and then fluorescent images of Calcein-AM/PI-stained cells were captured (green for live cells, and red for dead cells; scale bar = 100 μm).

C, D, E. Abrogation by 4-OH-E_1 of SAS-induced accumulation of cellular NO (**C**), ROS (**D**) and lipid-ROS (**E**). Cells were treated with SAS (0.5 mM) ± 4-OH-E_1 (5 μM) for 8 h, and then subjected to analytical flow cytometry. The left panels are the histograms, and the right panels are the quantitative values (n = 3).

F. Abrogation by 4-OH-E_1 of SAS-induced accumulation of cellular lipid-ROS (confocal microscopy, scale bar = 100 μm) in BRL-3A cells.

Quantitative data are presented as mean ± SD. ** or ## $P < 0.01$; n.s., not significant.

Supplementary Figure S11

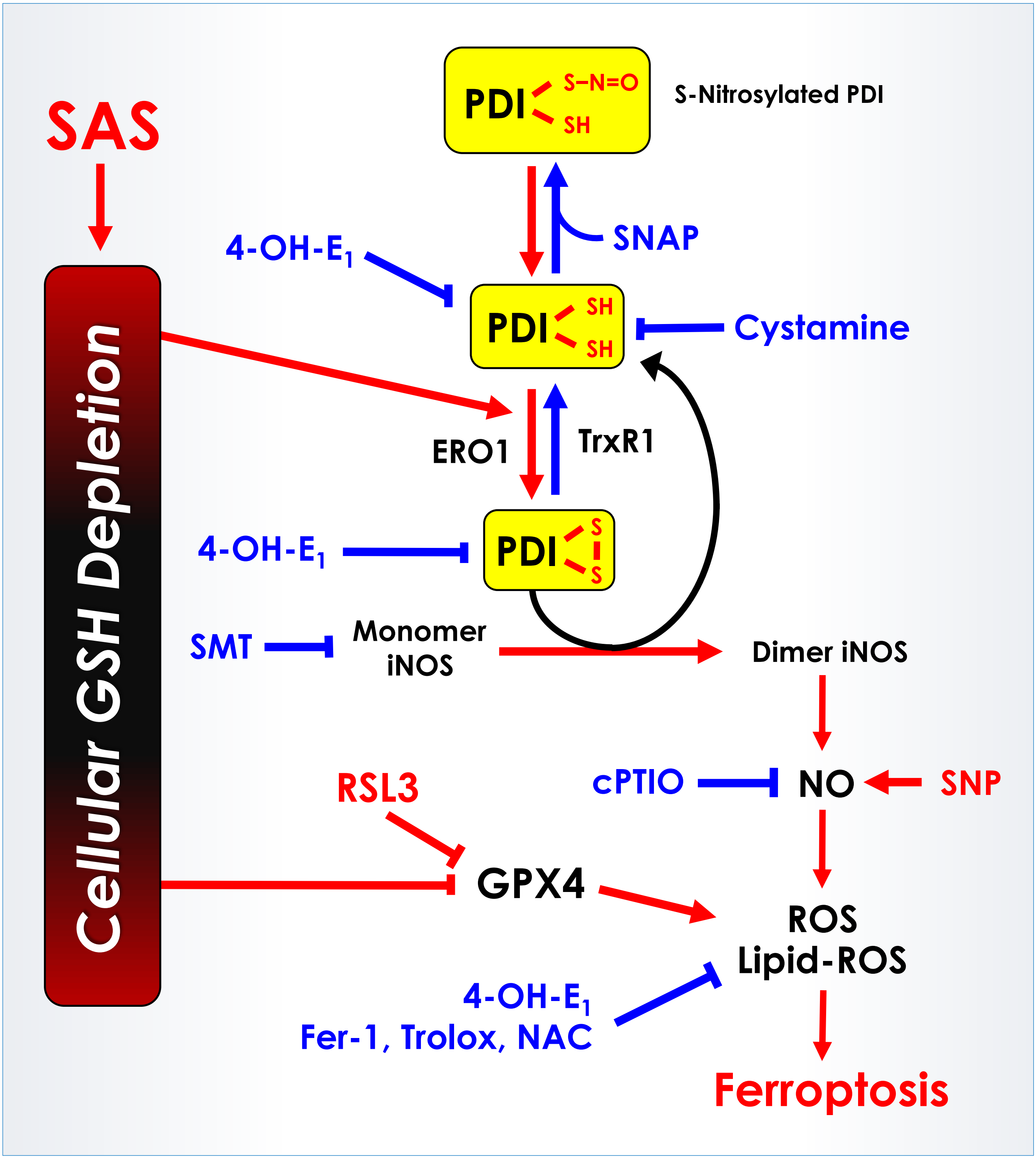

Supplementary Fig. S11. Schematic depiction of the role of PDI–iNOS/NO pathway in mediating SAS-induced ferroptosis. For details, please refer to the **DISCUSSION** section.
